# Targeting SKA3 suppresses the proliferation and chemoresistance of laryngeal squamous cell carcinoma via impairing PLK1–AKT axis-mediated glycolysis

**DOI:** 10.1038/s41419-020-03104-6

**Published:** 2020-10-26

**Authors:** Wei Gao, Yuliang Zhang, Hongjie Luo, Min Niu, Xiwang Zheng, Wanglai Hu, Jiajia Cui, Xuting Xue, Yunfeng Bo, Fengsheng Dai, Yan Lu, Dongli Yang, Yujia Guo, Huina Guo, Huizheng Li, Yu Zhang, Tao Yang, Li Li, Linshi Zhang, Rui Hou, Shuxin Wen, Changming An, Teng Ma, Lei Jin, Wei Xu, Yongyan Wu

**Affiliations:** 1 Shanxi Key Laboratory of Otorhinolaryngology Head and Neck Cancer, First Hospital of Shanxi Medical University, 030001 Taiyuan, Shanxi P.R. China; 2 Shanxi Province Clinical Medical Research Center for Precision Medicine of Head and Neck Cancer, First Hospital of Shanxi Medical University, 030001 Taiyuan, Shanxi P.R. China; 3 Department of Otolaryngology Head & Neck Surgery, First Hospital of Shanxi Medical University, 030001 Taiyuan, Shanxi P.R. China; 4 Key Laboratory of Cellular Physiology, Ministry of Education, Shanxi Medical University, 030001 Taiyuan, Shanxi P.R. China; 5 Department of Cell Biology and Genetics, Shanxi Medical University, 030001 Taiyuan, Shanxi P.R. China; 6 School of Basic Medical Science, Anhui Medical University, 230032 Hefei, Anhui P.R. China; 7 Department of Pathology, Shanxi Cancer Hospital, 030013 Taiyuan, Shanxi P.R. China; 8 Department of Otolaryngology Head & Neck Surgery, First Affiliated Hospital of Jinzhou Medical University, 121001 Jinzhou, Liaoning P.R. China; 9 Department of Otolaryngology Head & Neck Surgery, Dalian Municipal Friendship Hospital, 116100 Dalian, Liaoning P.R. China; 10 Department of Physiology, Shanxi Medical University, 030001 Taiyuan, Shanxi P.R. China; 11 Department of Biochemistry & Molecular Biology, Shanxi Medical University, 030001 Taiyuan, Shanxi P.R. China; 12 Department of Thyroid Surgery, Zhejiang University School of Medicine Second Affiliated Hospital, 310009 Hangzhou, Zhejiang P.R. China; 13 Harry Perkins Institute of Medical Research, QEII Medical Centre and Centre for Medical Research, University of Western Australia, Perth, WA 6009 Australia; 14 Department of Otolaryngology Head & Neck Surgery, Shanxi Bethune Hospital, 030032 Taiyuan, Shanxi P.R. China; 15 Department of Head and Neck Surgery, Chinese Academy of Medical Sciences Cancer Institute and Hospital, 100021 Beijing, P.R. China; 16 Department of Cellular and Molecular Biology, Beijing Tuberculosis and Thoracic Tumor Research Institute, 101149 Beijing, P.R. China; 17 School of Medicine and Public Health, The University of Newcastle, Callaghan, NSW 2308 Australia; 18 Department of Head and Neck Surgery, Shandong Provincial ENT Hospital Affiliated to Shandong University, 250022 Jinan, Shandong P.R. China; 19 Shandong Provincial Institute of Otolaryngology, 250022 Jinan, Shandong P.R. China; 20 Key Laboratory of Otolaryngology, Ministry of Health, Shandong University, 250022 Jinan, Shandong P.R. China

**Keywords:** Cancer metabolism, Tumour biomarkers, Head and neck cancer

## Abstract

Spindle and kinetochore-associated complex subunit 3 (SKA3) is a well-known regulator of chromosome separation and cell division, which plays an important role in cell proliferation. However, the mechanism of SKA3 regulating tumor proliferation via reprogramming metabolism is unknown. Here, SKA3 is identified as an oncogene in laryngeal squamous cell carcinoma (LSCC), and high levels of SKA3 are closely associated with malignant progression and poor prognosis. In vitro and in vivo experiments demonstrate that SKA3 promotes LSCC cell proliferation and chemoresistance through a novel role of reprogramming glycolytic metabolism. Further studies reveal the downstream mechanisms of SKA3, which can bind and stabilize polo-like kinase 1 (PLK1) protein via suppressing ubiquitin-mediated degradation. The accumulation of PLK1 activates AKT and thus upregulates glycolytic enzymes HK2, PFKFB3, and PDK1, resulting in enhancement of glycolysis. Furthermore, our data reveal that phosphorylation at Thr360 of SKA3 is critical for its binding to PLK1 and the increase in glycolysis. Collectively, the novel oncogenic signal axis “SKA3-PLK1-AKT” plays a critical role in the glycolysis of LSCC. SKA3 may serve as a prognostic biomarker and therapeutic target, providing a potential strategy for proliferation inhibition and chemosensitization in tumors, especially for LSCC patients with PLK1 inhibitor resistance.

## Introduction

Laryngeal squamous cell carcinoma (LSCC) is the second most frequent tumor of the respiratory tract or head and neck carcinoma^1,2^. Approximately 177,422 new cases of LSCC and 95,000 LSCC-related deaths have occurred worldwide in 2018^3^. Patients with LSCC showed severe impairments of important physiological functions such as vocalization, breathing, and swallowing, which diminish the quality of life. Though radiotherapy and chemotherapy have rapidly developed for numerous types of cancer^4^, the 5-year survival rate of patients with LSCC has decreased from 66% to 63% during the past 40 years^5^.

The onset of LSCC is occult, accounting for 60% incidence of patients with advanced stages (clinical stages 3 and 4) upon diagnosis^1,6^. Furthermore, those with the highest risk of recurrence are usually diagnosed in the first 2–3 years after surgery^7^. Moreover, secondary primary tumors are often diagnosed in patients whose initial lesion is controlled^8^. These major adverse factors significantly decrease the efficacy of long-term treatment and the survival rates of patients^9^. Thus, it is necessary to identify the molecular regulatory mechanisms underlying the progression of LSCC and the development of chemoresistance, by which to identify novel therapeutic targets, and developing new therapeutic strategies for LSCC patients.

Spindle and kinetochore-associated complex subunit 3 (SKA3) is a component of the SKA complex, which functions to stabilize the kinetochore–microtubule interaction in mitosis^10,11^. Knockdown of SKA3 expression activates the spindle assembly checkpoint with loss of sister chromatid cohesion, leading to mitotic arrest during metaphase^12,13^. SKA3 is implicated in the development and progress of lung adenocarcinoma, prostate cancer, cervical cancer, and breast cancer^14–17^. Here, we show that SKA3 is also highly expressed in LSCC, and higher levels of SKA3 are closely associated with poorer clinicopathological characteristics.

Polo-like kinase 1 (PLK1) is a serine/threonine-protein kinase, which is associated with mitotic spindle poles. As an early trigger that activates G2/M transition, PLK1 supports the functional maturation of the centrosome in late G2/early prophase and the establishment of the bipolar spindle^18^. PLK1 is considered a pro-oncogene as its role in driving cell-cycle progression and frequent overexpression in various types of cancer^19^. Moreover, it has been found that PLK1 was associated with drug resistance of several cancer chemotherapy drugs. Therefore, PLK1 inhibition has been considered as a potential strategy to develop novel drugs for the treatment of various types of cancer. However, PLK1 inhibitors, which initially succeeded in preclinical, have not been translated into clinical practice^20^. Hence, further elucidating the detailed regulatory mechanism(s) of PLK1 is required to pave a way for PLK1-targeted cancer treatment.

In this study, we have found that SKA3 binds and stabilizes PLK1 via inhibiting its ubiquitination and proteasome degradation, which in turn leads to activation of AKT. Moreover, activated SKA3–PLK1–AKT signaling enhances glycolysis, thus resulting in an increase of LSCC cell proliferation and resistance to chemotherapeutic drug treatment. Collectively, the data indicate a novel oncogenic pathway SKA3–PLK1–AKT in promoting LSCC cell proliferation and drug resistance via the enhancement of glycolysis.

## Materials and methods

### Ethics approval and consent to participate

The clinical sample study was approved by The Medical Ethics Committee of Shanxi Medical University (Approval number: 2015LL030). Informed consent as per institutional guidelines was obtained from all patients who agreed to participate in this study. Animal care, experimental procedures, and euthanasia followed the Health Guide for the Care and Use of Laboratory Animals approved by the Institutional Animal Care and Use Committee of Shanxi Medical University (Approval number: 2019SD136).

### Patients and tissue samples

We conducted transcriptome analyses of 53 primary LSCCs and matched adjacent normal mucosa tissues (ANM) (Supplementary Table S5). The tissues were acquired from patients undergoing surgery at the Department of Otolaryngology Head and Neck Surgery of The First Hospital Affiliated with Shanxi Medical University. LSCC was diagnosed using histology, and patients did not receive radiotherapy or chemotherapy before surgery. The histological types of LSCC were determined according to the World Health Organization (WHO) system. The tumor stage was defined according to the tumor site (T), lymph-node involvement (N), and distant metastatic (M) spread staging system (TNM) of the American Joint Committee on Cancer (AJCC, 8th edition).

### Plasmid construction and transfection

The wild-type and phosphorylation-site mutant SKA3 lentiviral expression plasmids were generated by inserting wild type or mutant SKA3 CDS fusion with a Flag tag sequence into the BamHI and XhoI sites of the lentiviral vector pLenti-puro (a gift from Ie-Ming Shih, Addgene plasmid # 39481). For SKA3-knockdown stable cell generation, three sgRNAs targeting *SKA3* exon 1 were synthesized and inserted into the pSpCas9(BB)-2A-Puro vector (Addgene plasmid # 62988). shRNA constructs targeting the top 50 upregulated genes used for high-content screening and the negative-control construct were purchased from Sigma-Aldrich (Munich, Germany). Wild-type and phosphorylation-site mutant SKA3 transient expression plasmids were constructed by inserting the corresponding expression frame into p3×FLAG-CMV-10 vector (Sigma-Aldrich). PLK1, PTEN, and Ubiquitin (Ub) expression plasmids were generated by inserting coding sequence into pCMV-HA vector (Clontech). Luciferase reporter plasmid pGL4.10-SKA3 was generated by inserting the promoter sequence (+100 to −1000 relative to transcription start site) into pGL4.10 vector. Transfection was performed using Lipofectamine 3000 (Invitrogen, Carlsbad, CA, USA) according to the manufacturer’s protocol.

### siRNA-mediated knockdown

For in vitro cell experiments, siRNAs targeting *SKA3*, *MYC*, *PLK1*, *PTEN*, *HK2*, *PFKFB3*, and *PDK1* were synthesized by Genepharma (Shanghai, China) and were transfected into cells using Lipofectamine 3000 reagent (ThermoFisher Scientific) according to the manufacturer’s instruction. The siRNA sequences used in this study were shown in Supplementary Table S6.

### High-content screening (HCS)

shRNA lentiviruses for the top 50 upregulated genes in LSCC tissues were produced in HEK293T cells. FD-LSC-1 cells stably expressing green fluorescence protein (GFP) were infected with viruses supernatant with 8 μg/ml polybrene. After 48 h of incubation, 2 μg/ml puromycin (Santa Cruz) was added for 2 days, then the equal number of cells were seeded into 96-well plates, and cell proliferation was measured on ImageXpress Micro Widefield High Content Screening System (Molecular Devices, Sunnyvale, CA) for 5 days. Sequences for shRNA constructs are listed in Supplementary Table S7.

### Co-immunoprecipitation

Co-immunoprecipitation (CoIP) was performed using a Co-Immunoprecipitation kit (ThermoFisher Scientific) following the manufacturer’s instructions. Briefly, cells were cultured in a 100-mm dish and collected at 90% confluence using IP lysis buffer with Protease Inhibitor Cocktail (ThermoFisher Scientific). After centrifugation, the supernatant was used for CoIP. Protein samples from the CoIP experiments were analyzed by western blotting or subjected to mass spectrometric analysis.

### Mass spectrometric analysis

CoIP was conducted with the Flag antibody. Protein samples were separated by 4–20% gradient SDS-PAGE (Genscript, Nanjing, China), then stained with Coomassie Brilliant Blue staining solution (Bio-Rad, Hercules, CA), and protein bands excised from the gel lanes were digested with trypsin and subjected to mass spectrometric analysis (MS) on a Q Exactive™ Hybrid Quadrupole-Orbitrap™ Mass Spectrometer (ThermoFisher scientific) by ProteinT (Tianjin) Biotech Co., Ltd. (Tianjin, China). Proteins were identified using Mascot software (version 2.3) with the Swissprot Human database (20207 sequences).

### Luciferase reporter assay

Cells were cultured in 48-well plates and cotransfected with SKA3 promoter luciferase reporter plasmid, *Renilla* luciferase plasmid pGL4.73 (Promega, Madison, WI), and siRNA targeting *MYC*. After 48-h transfection, luciferase activity was determined using the Dual-Luciferase Reporter Assay System (Promega) on SpectraMax i3x Multi-mode detection platform (Molecular Devices).

### Glycolysis-capacity assay

Extracellular acidification rate (ECAR) was assayed on a Seahorse XFp instrument (Agilent Technologies, Inc., Santa Clara, CA) according to the manufacturer’s instructions. Briefly, cells were seeded into the Seahorse eight-well plate at a density of 5000 cells/well, followed by culturing for 12 h. In all, 10 mM glucose, 2 μM oligomycin, and 2-deoxy-d-glucose were added in order, and ECAR was measured.

### Construction of the preclinical model and chemotherapy sensitivity analysis in vivo

SPF-grade male BALB/C nude mice (aged 7 weeks) were purchased from Beijing Vital River Laboratory Animal Technology Co., Ltd (Beijing, China) and maintained under SPF condition (TECNIPLAST S.p.A., Italy). The nude mice were assigned to each group randomly. For xenograft tumor formation, mice were subcutaneously injected with SKA3 overexpression or knockdown cells, and with NC or Vector cells as control (six mice per group, 4 × 10^6^ NC and SKA3-KD cells were inoculated, and 2 × 10^6^ vector and SKA3-OE cells were inoculated for each mouse). For chemotherapy drug treatment, mice were randomly divided into the control- and cisplatin-treatment group (seven mice per group, 4 × 10^6^ cells were inoculated for each mouse). Cisplatin was administered by intraperitoneal (i.p.) injection at 60 μg/mouse per week for 3 weeks. The tumor sizes were measured every 2 days with calipers, and the volume was determined by long diameter × short diameter^2^/2. Tumors were harvested, weighed, and photographed at the end of treatment.

### SKA3-knockout mice

SKA3-knockout mice were generated by CRISPR/Cas9 method (Cyagen Biosciences (Suzhou) Inc., Jiangsu, China). The paired-guide RNA targeting mouse Ska3 gene (exons 2–6) was designed. The guide RNA sequences were gRNA1: GAGCUGUGCGACUGCUAGUAAGG; gRNA2: GGCCACUCUUAGGGAUGCGUGGG. Cas9 mRNA and gRNA generated by in vitro transcription were co-injected into fertilized eggs, and the transgenic embryos were planted into pseudopregnant mice. Successful mutation of the SKA3 gene was identified through PCR genotyping using tail DNA. PCR products were further verified by DNA sequencing. The genotyping primers were as follows: forward primer, 5′-TCACCAAGTGTTTGTTAGTCTGTAGTCTCAC-3′; reverse primer, 5′-ATCTGAATAGTGGCTGCCATACCAAAT-3′. The positive female founder mice and wild-type male mice were bred to obtain F1 SKA3 heterozygote mice. Male and female SKA3 heterozygote mice were then crossed to obtain SKA3 homozygote mice. A total of 137 heterozygote mice were produced, but there was no mouse with a homozygous deletion of SKA3. According to the probability of genetics reported previously, we proposed that the homozygous deletion of SKA3 is embryonically lethal. Moreover, the embryos from 5.5 days post cohabitation (dpc) to 16.5 dpc were genotyped by PCR, and no homozygous embryo was detected. For mouse-embryo genotyping, the tail tips were isolated, and the SKA3 mutation was identified by PCR amplification using the TransDirect Mouse Genotyping Kit (TransGen Biotech Co., Ltd., Beijing, China). Therefore, wild-type and SKA3 heterozygote mice were used in this study.

### TCGA data analysis

Expression data, clinical features, and overall survival probability of head and neck squamous cell carcinoma (HNSCC) were analyzed with GEPIA2 (http://gepia2.cancer-pku.cn/)^21^. Fragments per kilobase million (FPKM) for SKA3 and PLK1 in LSCC were obtained from The Cancer Genome Atlas (TCGA) HNSCC cohort (https://portal.gdc.cancer.gov/projects/TCGA-HNSC), followed by expression correlation analysis.

### Statistical analysis

Data were analyzed using the IBM-SPSS Statistics software package (version 20, IBM-SPSS Statistics, Armonk, NY, USA). All data represent the results of at least three independent experiments, and are expressed as the mean ± standard deviation (SD), unless stated. The sample sizes were sufficient for statistical analysis. The independent *t* test was used to compare baseline variables, and Fisher’s exact test was used to analyze numerical data. Overall survival was defined as the time from surgery to the date of death from laryngeal carcinoma or the date of the last follow-up. Survival analysis was performed using the Kaplan–Meier method with a log-rank test. *P* < 0.05 was considered to indicate a significant difference. All tests were two-tailed.

The following experiments are presented in the Supplemental Materials and Methods: transcriptome- sequencing analysis, cell culture, generation of stably overexpressing and knockdown cells, immunofluorescence staining, cell proliferation analysis, colony-formation assay, RNA extraction, reverse transcription, real-time quantitative PCR (qPCR), western blotting, immunohistochemical staining and analysis, and chromatin immunoprecipitation (ChIP). Antibodies and reagent information is shown in Supplemental Materials and Methods.

## Results

### SKA3 is highly expressed in LSCC and associated with poor prognosis

**Fig. 1:**
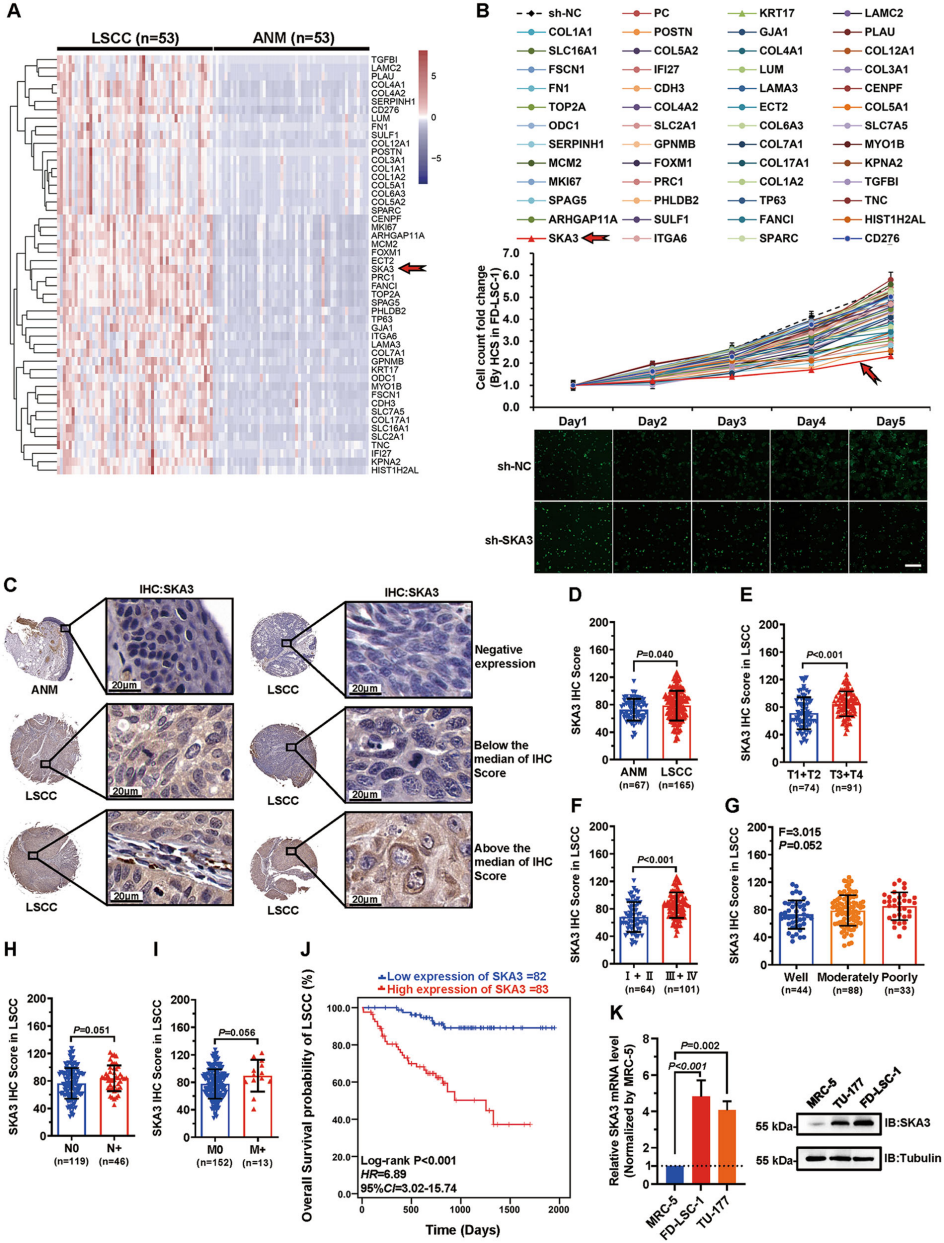
SKA3 is highly expressed in laryngeal squamous cell carcinoma (LSCC) with a poor prognosis. **A** Transcriptome sequencing of 53 pairs of LSCC and matched adjacent normal mucosal (ANM) tissues was performed to identify the top 50 upregulated genes in LSCC for cluster analysis. **B** The top 50 upregulated genes were individually knocked down via infection with shRNA viruses in the LSCC cell line FD-LSC-1; cell proliferation was measured using a high-content screening system for 5 days. Top: fold change of the cell proliferation of each group normalized to day 1. Bottom: representative cell images from high-content screening. NC negative control, PC positive control. Scale bar, 200 μm. **C** SKA3 expression in paraffin sections of an LSCC tissue array (*n* = 165) was detected using IHC. Representative images of IHC and standard of IHC scoring are shown. Scale bar, 20 μm. **D** The SKA3 protein level in LSCC and ANM tissues was determined using IHC. **E**–**I** Association analysis of the SKA3 protein levels with T stage (**E**), clinical stage (**F**), degree of pathological differentiation (**G**), N stage (**H**), and M stage (**I**) of LSCC. **J** Kaplan–Meier analysis of the association of the SKA3 levels with the overall survival of patients with LSCC. The SKA3 expression levels were divided into low or high groups according to the median IHC score (*n* = 165). **K** RT–qPCR and western blot analysis of endogenous SKA3 in MRC-5, TU-177, and FD-LSC-1 cells. The data are expressed as means ± SD of three independent experiments in (**B**) and (**K**).

To identify novel therapeutic targets of LSCC, RNA sequencing was performed to compare gene expression profiles between human LSCC tissues and paired adjacent normal mucosal tissues (ANM) (*n* = 53). Furthermore, the role of the top 50 upregulated genes (Fig. 1A; Supplementary Table S1) in LSCC proliferation was investigated by shRNA-mediated knockdown and high-content screening. Among the top 50 upregulated genes, knockdown of SKA3 most significantly reduced LSCC cell proliferation (Fig. 1B). Thus, we focused on the function and mechanism of SKA3 in this study. High SKA3 protein expression was further confirmed in an enlarged cohort of 165 LSCC samples using immunohistochemical staining (IHC) analysis (Fig. 1C, D).

To identify the clinical significance of SKA3 upregulation in LSCC, the correlation between SKA3 upregulation and LSCC patient clinical features was analyzed according to the IHC score (Supplementary Table S2). The SKA3 expression was positively correlated with T and clinical stages (*P* < 0.001) (Fig. 1E, F). The upregulation of SKA3 was mildly correlated with poor differentiation (*P* = 0.052), cervical lymph-node metastasis (*P* = 0.051), and distant metastasis (*P* = 0.056) (Fig. 1G–I). Of note, the higher expression of SKA3 protein was significantly associated with poorer survival of LSCC patients (Fig. 1J). Consistent with these results, we found that SKA3 expressed at higher levels in the LSCC cell lines TU-177 and FD-LSC-1 compared with the human normal lung fibroblast cell line MRC-5 (Fig. 1K). Collectively, these data indicated that the upregulation of SKA3 is crucial in LSCC development and progression.

### SKA3 promotes LSCC cell proliferation and xenograft tumor growth

**Fig. 2:**
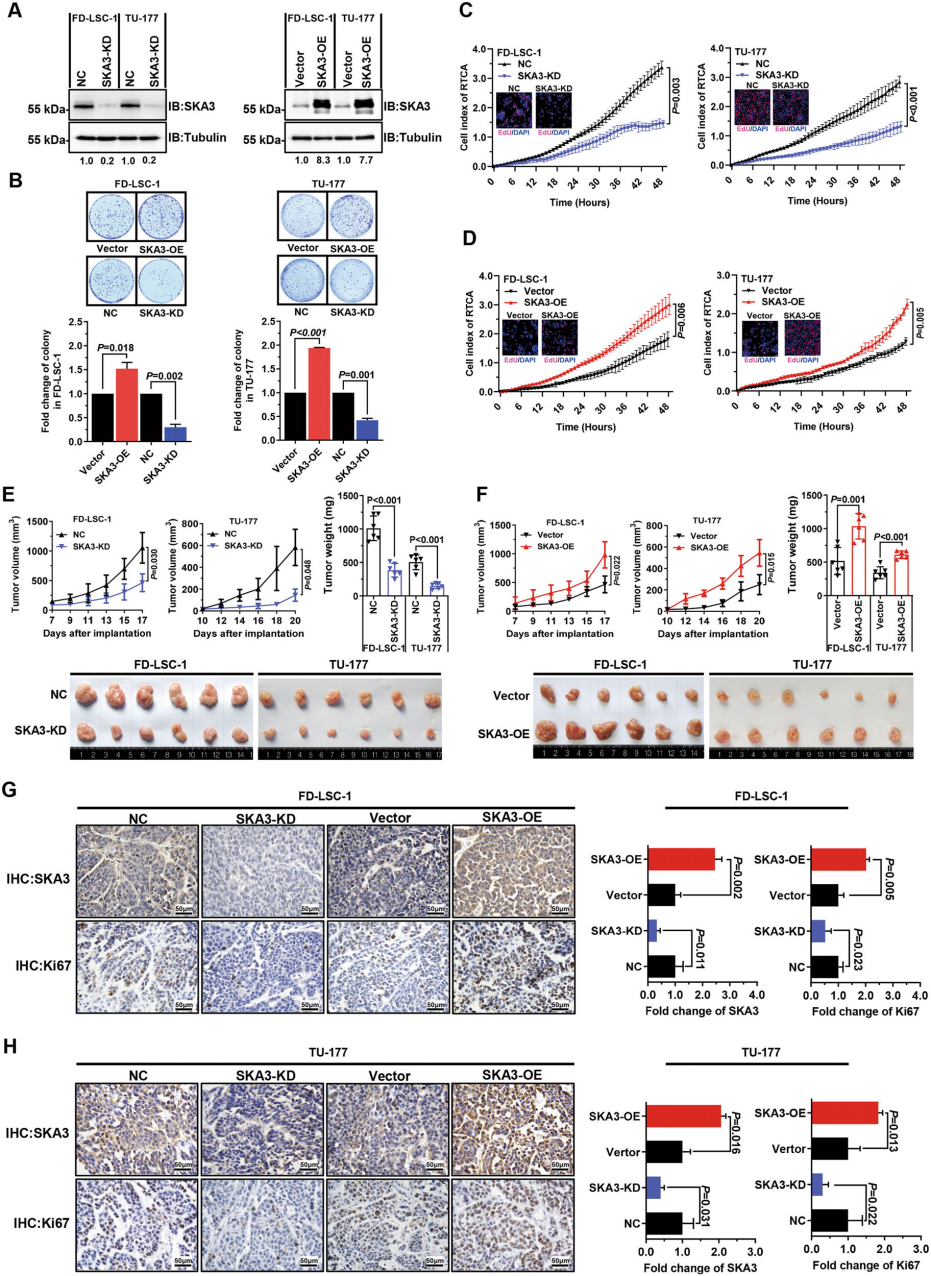
SKA3 promotes laryngeal squamous cell carcinoma (LSCC) proliferation and growth of LSCC xenograft tumors. **A** Western blot analysis of SKA3 expression in SKA3-knockdown (left) or SKA3-overexpressing (right) FD-LSC-1 and TU-177 cells. **B** Overexpression of SKA3 promoted colony formation by the FD-LSC-1 and TU-177 cell lines (upper), while silencing of SKA3 expression suppressed colony formation (lower). **C**, **D** FD-LSC-1 and TU-177 cells were transfected with siRNAs targeting SKA3 (SKA3-KD), negative-control siRNAs (NC) (**C**), SKA3-overexpression plasmid (SKA3-OE), or empty vector (vector) (**D**). Twenty-four hours after transfection, the cells were reseeded in real-time cell analysis (RTCA) plates or 96-well plates, and proliferation was measured using the RTCA system and EdU staining (merged images are superimposed on the RTCA curve). **E**, **F** Silencing (**E**) or overexpression (**F**) of SKA3 inhibited or promoted the growth, respectively, of tumors generated by xenografted FD-LSC-1 and TU-177 LSCC cell lines in nude mice. Tumor growth curves and end-point weights are shown (upper). Representative images (lower) are displayed (six mice per group). **G**, **H** IHC analysis of the expression of SKA3 and the marker of proliferation Ki67 in sections of tumors generated by xenografted FD-LSC-1 (**G**) and TU-177 (**H**) cells. Representative IHC images (left) and semiquantitative analysis of IHC images using QuPath software (right) are displayed. Scale bar, 50 µm. The data are expressed as means ± SD of three independent experiments.

To identify the functional significance of SKA3 upregulation in LSCC, we generated LSCC cell sublines stably overexpressing SKA3 (SKA3-OE) or knocking down SKA3 (SKA3-KD) (Fig. 2A). While SKA3 knockdown inhibited, SKA3 overexpression promoted the clonogenic capability of LSCC cells (Fig. 2B). Real-time cell analysis (RTCA) and EdU staining assays confirmed these findings (Fig. 2C, D). To identify the function of SKA3 in vivo, the SKA3-overexpressing and -knockdown LSCC cell lines were subcutaneously injected into nude mice. While SKA3 silencing retarded, SKA3 overexpression accelerated tumor growth (Fig. 2E, F). As expected, these changes were associated with alterations in the proportion of Ki67-positive cells (Fig. 2G, H), indicating that SKA3 sustained cancer cell proliferation in vivo. Together, these findings reveal that SKA3 stimulated LSCC cell proliferation in vitro and heightened the tumorigenicity in vivo.

### SKA3 enhances glycolysis of LSCC cells

**Fig. 3:**
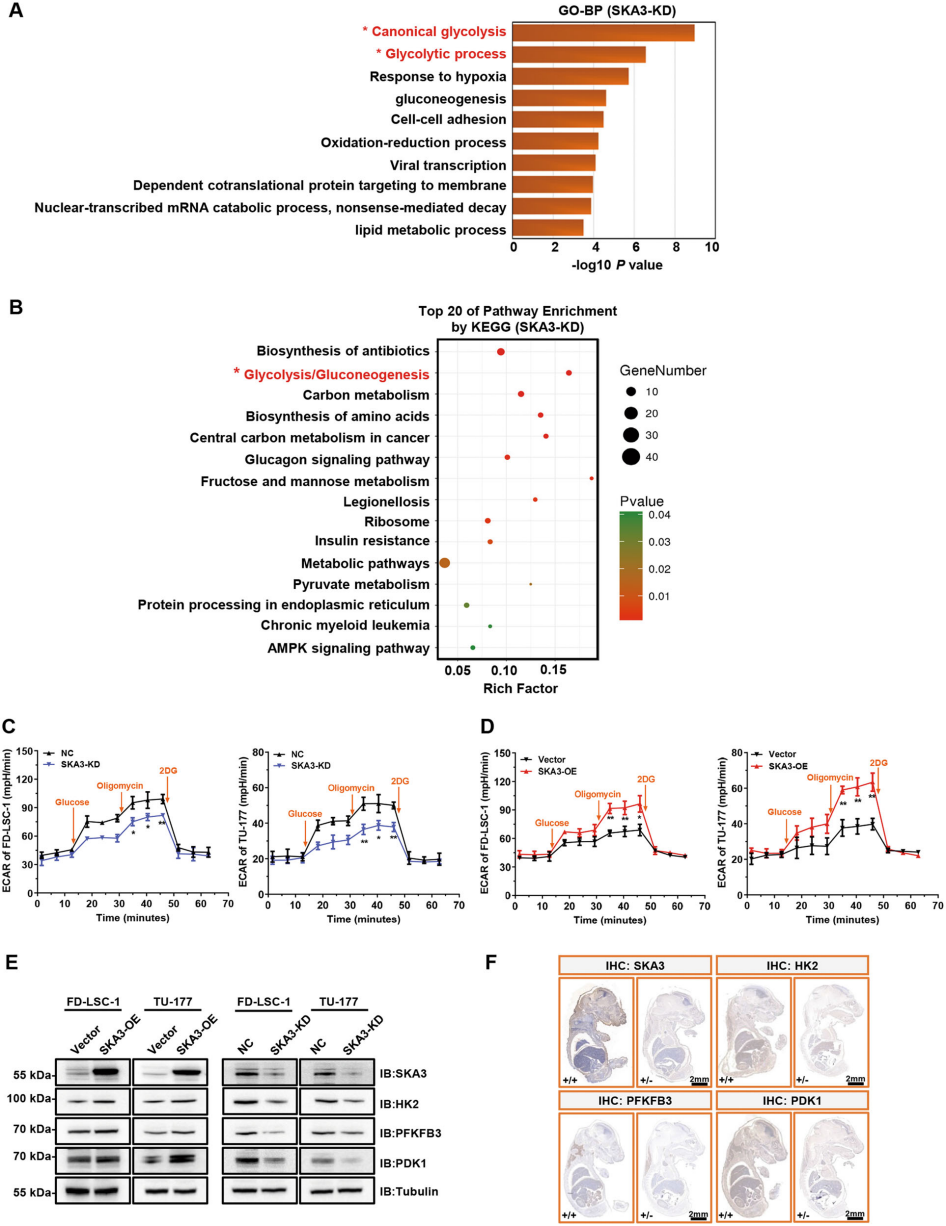
SKA3 promotes glycolysis in laryngeal squamous cell carcinoma (LSCC) cells. **A** RNA-seq was performed on SKA3-knockdown (SKA3-KD) TU-177 cells. GO analysis of downregulated genes in SKA3-KD TU-177 cells; the glycolytic process is indicated with an asterisk. **B** KEGG pathway analysis of downregulated genes in SKA3-KD TU-177 cells. **C**, **D** Glycolysis capacity in SKA3-knockdown or SKA3-overexpressing FD-LSC-1 (**C**) and TU-177 cells (**D**) was measured using a Seahorse energy metabolism instrument (Seahorse XFP). The ECAR value indicates the rate of lactic acid production during glycolysis. **E** Knockdown or overexpression of SKA3 in FD-LSC-1 and TU-177 LSCC cells. The expression levels of HK2, PFKFB3, and PDK1 were determined using western blot analysis. **F** Representative whole-mount IHC staining images of SKA3, HK2, PFKFB3, and PDK1 in the embryos of Ska3-WT (+/+) and KO (+/−) mice on day 16.5 of gestation. Scale bar, 2 mm. The data are expressed as means ± SD of three independent experiments in (**C**) and (**D**). *P < 0.05; **P < 0.001.

To identify the molecular mechanism(s) underlying SKA3 function in the regulation of LSCC cell proliferation, we conducted transcriptome sequencing to identify gene expression alterations caused by SKA3 knockdown (Supplementary Table S3). Strikingly, glycolysis-related pathways, canonical glycolysis, and glycolytic processes were identified as the most significantly enriched pathways by Gene Ontology (GO) and Kyoto Encyclopedia of Genes and Genomes (KEGG) analyses (Fig. 3A, B).

Experimentally, we measured the extracellular acidification rate (ECAR) using the Seahorse XF Glycolytic Rate Assay in cells with or without SKA3 knockdown or overexpression. Strikingly, SKA3 silencing decreased, whereas SKA3 overexpression increased the rates of extracellular acidification in LSCC cells (Fig. 3C, D). Consistently, the culture medium colors of FD-LSC-1 and TU-177 cells with SKA3 overexpression turned to yellow (Supplementary Fig. S1), indicating acidification of the culture medium in cells with SKA3 overexpression. These results indeed revealed that SKA3 promotes glycolysis in LSCC cells. In support, silencing of SKA3 specifically downregulated the expression of three glycolytic enzymes HK2, PFKFB3, and PDK1, but not other glycolysis-related proteins in LSCC cell lines (Fig. 3E and Supplementary Fig. S2). To further confirm the regulatory role of SKA3 in glycolysis in vivo, SKA3-hemizygous knockout (+/−) mice were generated using CRISPR/Cas9 technology (Supplementary Fig. S3a, b). Consistently, the expression levels of HK2, PFKFB3, and PDK1 were significantly decreased in SKA3-hemizygous knockout (+/−) mouse embryos in comparison with wild-type (+/+) mouse embryos (Fig. 3F).

### SKA3 binds PLK1, thus promoting glycolysis via AKT activation

**Fig. 4:**
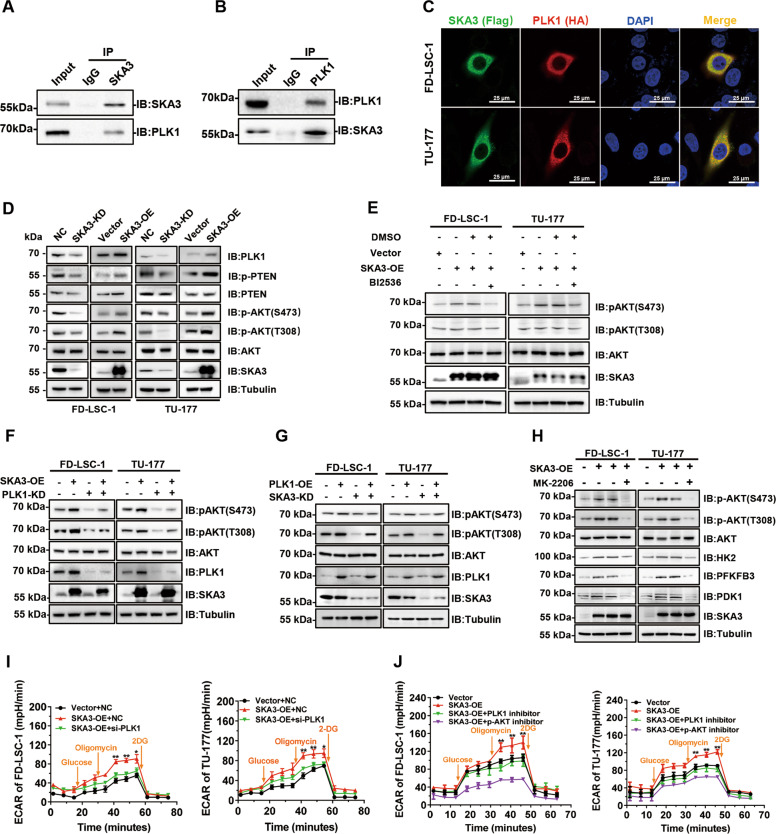
SKA3 binds PLK1, thus promoting glycolysis via AKT activation. **A**, **B** CoIP-western blot analysis of TU-177 cells. The lysates were incubated with anti-SKA3 (**A**) and anti-PLK1 (**B**) antibodies, and the fractionated immunoprecipitates were probed on western blots using antibodies against SKA3 and PLK1. **C** FD-LSC-1 and TU-177 cells were cotransfected with a Flag-tagged SKA3-overexpression plasmid and a HA-tagged PLK1-overexpression plasmid for 48 h. Immunofluorescence staining was performed using anti-FLAG and anti-HA antibodies, and cell nuclei were stained using DAPI (blue). Subcellular localization of SKA3 (green) and PLK1 (red) was observed using a laser confocal microscope. **D** Western blot analysis of the expression of PLK1, phosphorylated PTEN, and phosphorylated AKT (Ser473 and Thr308) in SKA3-knockdown or SKA3-overexpressing FD-LSC-1 and TU-177 cells, respectively. **E** FD-LSC-1 and TU-177 cells were transfected with the SKA3-overexpression plasmid for 36 h and then were treated with the PLK1 inhibitor BI2536 (30 nM) or an equal volume of DMSO for 12 h. AKT and phosphorylated AKT (Ser473 and Thr308) levels were determined by western blot analysis. **F** Knockdown of PLK1 and overexpression of SKA3 in FD-LSC-1 and TU-177 cells. The levels of total and phosphorylated AKT (Ser473 and Thr308) were determined by western blot analysis. **G** Effect of SKA3 knockdown and PLK1 overexpression on the levels of phosphorylated AKT (Ser473 and Thr308) determined by western blot analysis. **H** FD-LSC-1 and TU-177 cells were transfected with the SKA3-overexpression plasmid for 36 h, and then treated with the AKT inhibitor MK-2206 (20 µM) for 12 h. Expression of phosphorylated AKT (Ser473 and Thr308), HK2, PFKFB3, and PDK1 was determined by western blot analysis. **I** LSCC cells were cotransfected with siRNAs targeting PLK1 and an SKA3-overexpression plasmid for 48 h, at which time glycolysis was measured using the Seahorse energy metabolism instrument. **J** LSCC cells were transfected with the SKA3-overexpression plasmid for 36 h, and then treated with the PLK1 inhibitor BI2536 (30 nM) or AKT inhibitor MK-2206 (20 µM) for 12 h. Glycolysis was measured using a Seahorse energy metabolism instrument. The data are expressed as means ± SD of three independent experiments in (**I**) and (**J**). *P < 0.05; **P < 0.001.

To uncover the mechanism(s) of SKA3 in regulating glycolysis, co-immunoprecipitation (CoIP) plus mass spectrometry were performed, and PLK1 was identified as a novel SKA3-binding protein among sixty-eight identified candidates (Supplementary Fig. S4 and Supplementary Table S4), including SKA-complex components SKA1 and SKA2 reported previously^10,13,23^. Indeed, SKA3 bound and was colocalized with PLK1 in the cytoplasm of LSCC cells (Fig. 4A–C). It has been reported that the phosphorylation of PTEN (p-PTEN) driven by PLK1 led to its inactivation, thus enhancing the phosphorylation of AKT (p-AKT), the active form of AKT. Notably, high levels of p-PTEN resulted in the increased p-AKT levels and enhanced glycolysis^24,25^. Therefore, we hypothesized that SKA3 promoted glycolysis via its binding to PLK1. Strikingly, while overexpression of SKA3 caused the increase in PLK1 and phosphorylated AKT (p-AKT) levels and the increase in phosphorylated PTEN (p-PTEN) levels, SKA3 knockdown resulted in the decrease in PLK1 and p-AKT levels and the decrease in p-PTEN levels (Fig. 4D). Of note, the upregulation of p-AKT levels caused by SKA3 overexpression was reversed by treatment with the PLK1 inhibitor BI2536 or siRNA knockdown of PLK1 (Fig. 4E, F), indicative of PLK1 dependence of SKA3-mediated AKT activation. In contrast, the decrease in p-AKT levels driven by SKA3 silencing was rescued by overexpression of PLK1 (Fig. 4G). Taken together, the results strongly suggest that SKA3 regulates AKT activation through PLK1.

Next, we investigated whether SKA3-regulated glycolysis was dependent on PLK1–AKT signaling. Strikingly, upregulation of glycolytic enzymes HK2, PFKFB3, and PDK1 caused by SKA3 overexpression was reversed by the AKT inhibitor MK-2206 treatment (Fig. 4H). Moreover, either the siRNA knockdown of PLK1 or inhibition of AKT also diminished the glycolysis activation derived from SKA3 overexpression (Fig. 4I, J). Accordingly, knockdown of HK2, PFKFB3, or PDK1 partially reversed the increase in glycolytic activities caused by SKA3 overexpression (Supplementary Fig. S5a, b). Collectively, SKA3-mediated glycolysis activation relies on the PLK1–AKT–HK2/PFKFB3/PDK1 signaling axis.

### SKA3 stabilizes PLK1 protein through blocking its ubiquitination

**Fig. 5:**
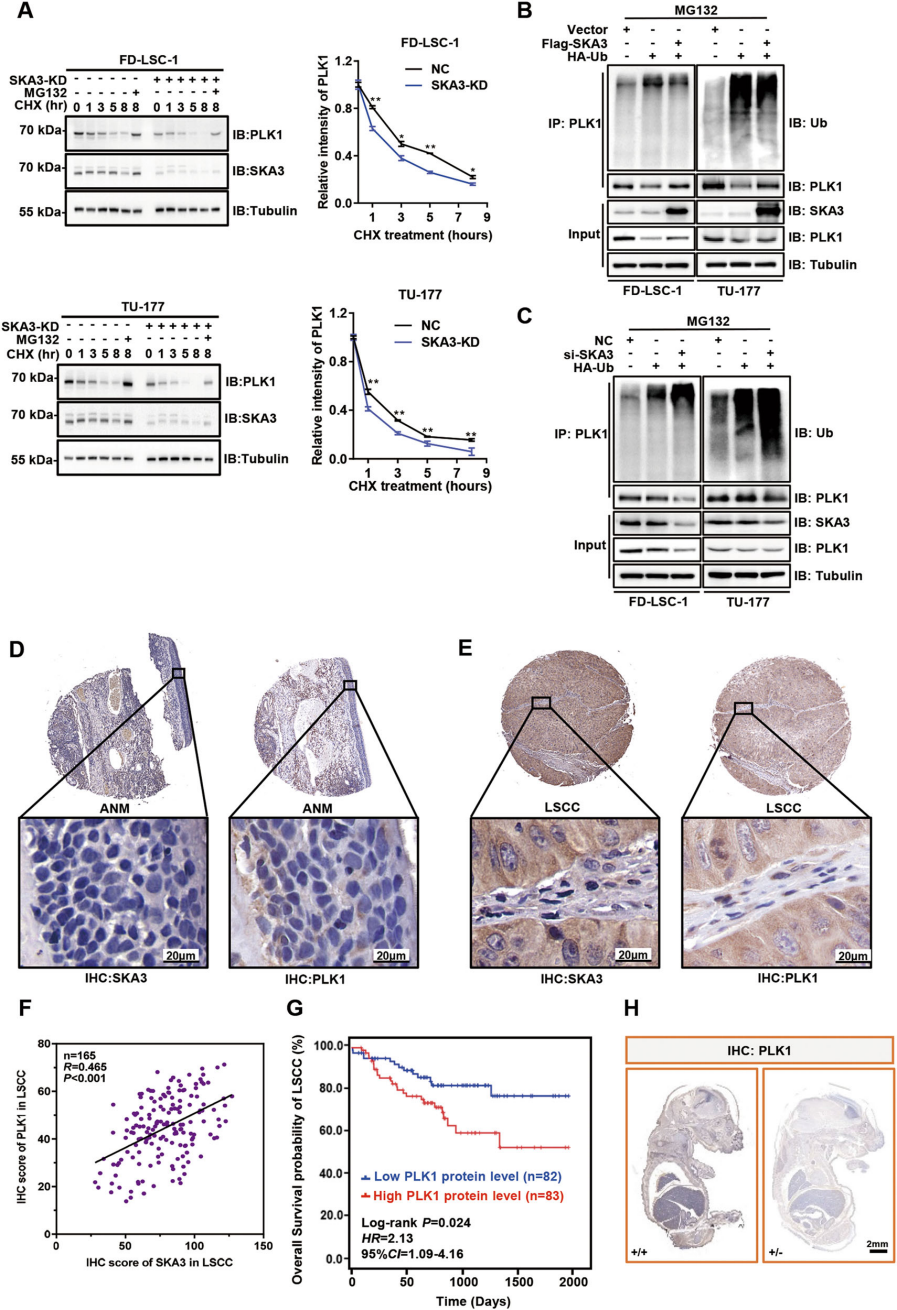
SKA3 enhances PLK1 protein stability by blocking its ubiquitylation. **A** FD-LSC-1 and TU-177 cells were transfected with siRNAs targeting SKA3 or negative-control siRNAs for 48 h, and then treated with the protein synthesis inhibitor cycloheximide (CHX, 40 µg/ml) for 0, 1, 3, 5, and 8 h. One group of cells was simultaneously treated with CHX (40 µg/ml) and the proteasome inhibitor MG132 (20 µM) for 8 h. Expression of SKA3 and PLK1 was measured by western blot analysis. The protein bands were quantified by ImageJ program. The data are expressed as means ± SD of three independent experiments. *P < 0.05; **P < 0.001. **B**, **C** Effect of SKA3 overexpression or knockdown on the ubiquitylation of PLK1. Cells were cotransfected with the SKA3-overexpression plasmid or SKA3-specific siRNA and HA-tagged ubiquitin expression plasmid for 48 h. CoIP was performed using an anti-PLK1 antibody followed by western blot analysis to assess the effects of SKA3 overexpression (**B**) or knockdown (**C**) on the ubiquitylation of PLK1 protein. **D**, **E** Representative images of IHC of SKA3 and PLK1 expression in 165 ANM (**D**) and LSCC (**E**) tissues. **F** Pearson correlation analysis of PLK1 with the SKA3 protein level in 165 LSCC samples using the IHC score. **G** Kaplan–Meier survival analysis of the association of PLK1 levels with the overall survival of LSCC patients. The PLK1-expression levels were divided into low or high groups according to the median IHC score (*n* = 165). **H** Representative whole-mount IHC staining images of PLK1 in the embryos of WT (+/+) and KO (+/−) mice on day 16.5 of gestation.

Cycloheximide (CHX)-chase assays were performed to identify the functional consequence of the binding between SKA3 and PLK1. Interestingly, SKA3 knockdown accelerated PLK1 protein turnover rates in the presence of the protein synthesis inhibitor CHX. However, SKA3 silencing-mediated PLK1 protein degradation was diminished by the proteasome inhibitor MG132 (Fig. 5A), indicating that SKA3 inhibits PLK1 degradation from the ubiquitin–proteasome pathway (UPP). In support, overexpression of SKA3 decreased the polyubiquitination of PLK1, whereas SKA3 knockdown increased the polyubiquitination of PLK1 in FD-LSC-1 and TU-177 cells (Fig. 5B, C). In accordance with SKA3, PLK1 was upregulated and positively correlated with SKA3 in 165 LSCC tissues (Fig. 5D–F). Analysis of single-cell transcriptomic data also confirmed that PLK1 expression was positively correlated with SKA3 levels in head and neck cancer (Supplementary Fig. S6) (GEO accession number: GSE103322). Moreover, higher expression levels of PLK1 protein were also significantly associated with poorer overall survival of LSCC patients (Fig. 5G). Supportively, the expression of PLK1 protein was also dramatically suppressed in SKA3-hemizygous knockout (+/−) mouse embryos in comparison with wild-type (+/+) mouse embryos (Fig. 5H).

### Phosphorylation of SKA3 at Thr360 is essential for its binding to PLK1

**Fig. 6:**
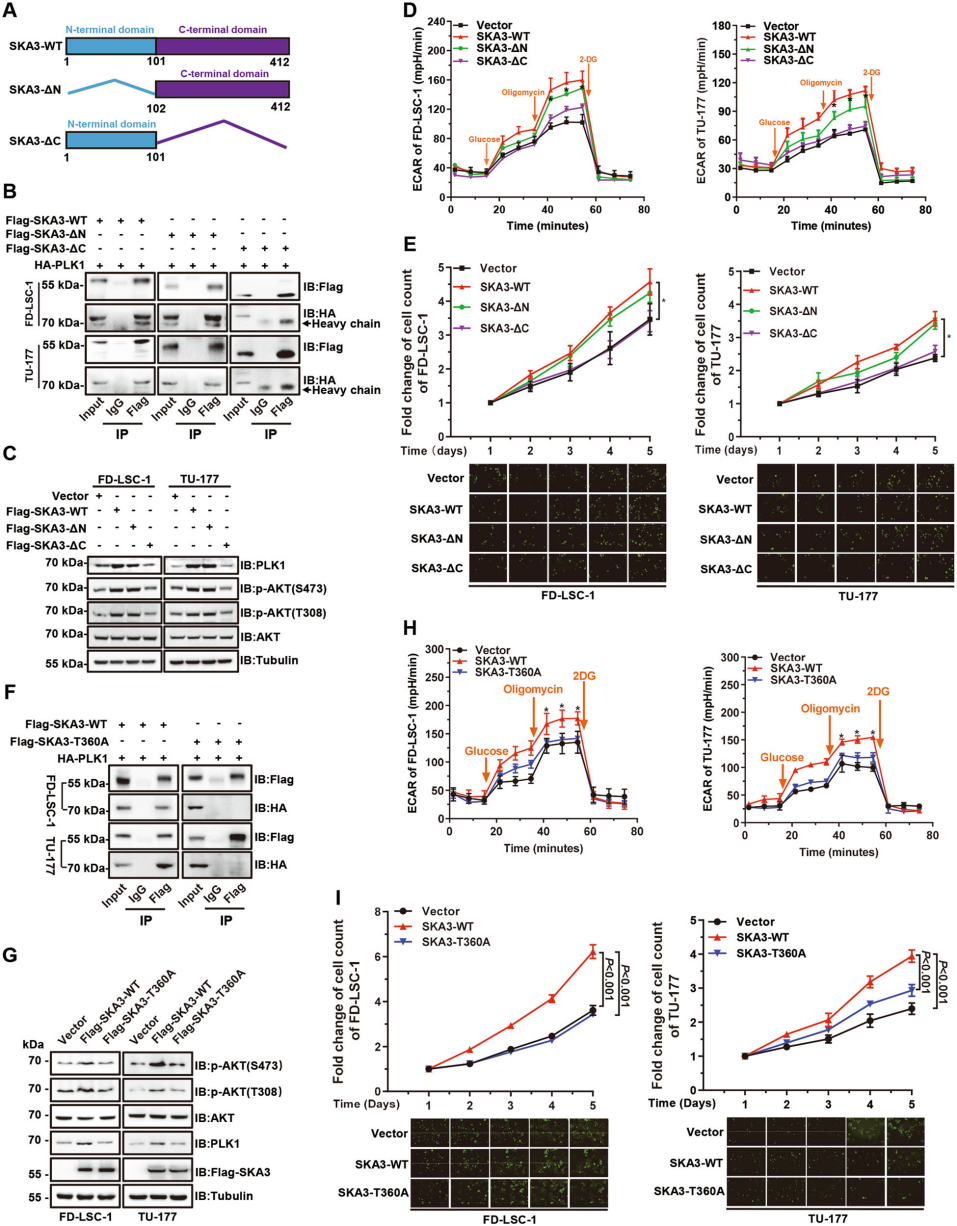
Threonine 360 of SKA3 protein is essential for PLK1 binding and subsequent biological functions. **A** Schematic diagram of wild-type SKA3 protein (SKA3-WT), SKA3 N-terminal domain deletion (SKA3-ΔN), and SKA3 C-terminal domain deletion (SKA3-ΔC). Numbers indicate amino acid positions. **B** LSCC cells were cotransfected with HA-tagged PLK1-expression plasmid and FLAG-tagged SKA3-WT-, SKA3-ΔN-, and SKA3-ΔC-expressing plasmids for 48 h; CoIP was performed using a FLAG antibody, and PLK1 was detected by western blotting. **C** LSCC cells were transfected with SKA3-WT, SKA3-ΔN, and SKA3-ΔC-expression plasmid or an empty vector for 48 h, and the expression levels of PLK1 and phosphorylated AKT (Ser473 and Thr308) were determined by western blotting. **D** FD-LSC-1 and TU-177 cells were transfected with SKA3-WT-, SKA3-ΔN-, and SKA3-ΔC-expression plasmid, or an empty vector for 48 h, and glycolysis was measured. **E** FD-LSC-1 and TU-177 cells stably expressing the Flag-tagged wild-type SKA3 or domain deletion, or the empty vector, were seeded in 96-well plates. Cell proliferation was measured using a high-content screening system for 5 days. Top: fold changes in cell proliferation relative to day 1; bottom: representative cell images. Scale bar, 200 μm. **F** FD-LSC-1 and TU-177 cells were cotransfected with HA-tagged PLK1-expression and the SKA3-WT plasmid (Flag-SKA3-WT) or a phosphorylation-site mutant (T360A) expression plasmid (Flag-SKA3–T360A) for 48 h. The cell lysates were immunoprecipitated with an anti-Flag antibody, and the precipitates were analyzed using western blot analysis with the indicated antibodies. **G** FD-LSC-1 and TU-177 cells were transfected with the Flag-SKA3-WT or Flag-SKA3–T360A-expression plasmid for 48 h. The levels of PLK1 and phosphorylated AKT (Ser473 and Thr308) were determined using western blot analysis. **H** FD-LSC-1 and TU-177 cells were transfected with the Flag-SKA3-WT or Flag-SKA3–T360A-expression plasmid, or the empty vector for 48 h, and then glycolytic capacity was measured. **I** FD-LSC-1 and TU-177 cells stably expressing the wild-type SKA3 or SKA3–T360A were seeded in 96-well plates. Cell proliferation was measured using a high-content screening system for 5 days. Top: fold changes in cell proliferation relative to day 1; bottom: representative cell images. Scale bar, 200 μm. The data are expressed as means ± SD of three independent experiments. *P < 0.05.

To further study the regulatory mechanism(s) of SKA3 on PLK1 and glycolysis, wild-type SKA3 (SKA3-WT), N-terminal domain deletion mutant (SKA3-ΔN), and C-terminal domain deletion mutant (SKA3-ΔC) plasmids were constructed (Fig. 6A and Supplementary Fig. S7). Deletion mapping experiments with SKA3 mutants showed that deleting the SKA3 C-terminal domain but not other regions abolished its association with PLK1 (Fig. 6B). Accordingly, overexpression of SKA3-WT and SKA3-ΔN, but not SKA3-ΔC, upregulated PLK1 and p-AKT levels, thus increasing glycolytic rates (Fig. 6C, D). Consequently, overexpression of SKA3-WT and SKA3-ΔN, but not SKA3-ΔC, significantly promoted LSCC cell proliferation (Fig. 6E).

SKA3 protein contains multiple phosphorylation sites, and phosphorylation at Thr360 is critical for its binding to Ndc80 protein^26^. We tested whether phosphorylation at Thr360 affects the interaction of SKA3 with PLK1. We found that the SKA3–T360A mutant (Thr360 was substituted with alanine) abolished the binding of SKA3 to PLK1, which decreased PLK1 and phosphorylated AKT levels compared with those of wild-type SKA3 (Fig. 6F, G). Further, the glycolysis and proliferation of LSCC cells were reduced in SKA3–T360A transfectants compared with wild-type SKA3 (Fig. 6H, I). Together, these data suggest that the phosphorylation site Thr360 of SKA3 was essential for the interaction between SKA3 with PLK1 that mediated the glycolytic regulatory activity of SKA3.

### SKA3 protects LSCC cells against cisplatin treatment

**Fig. 7:**
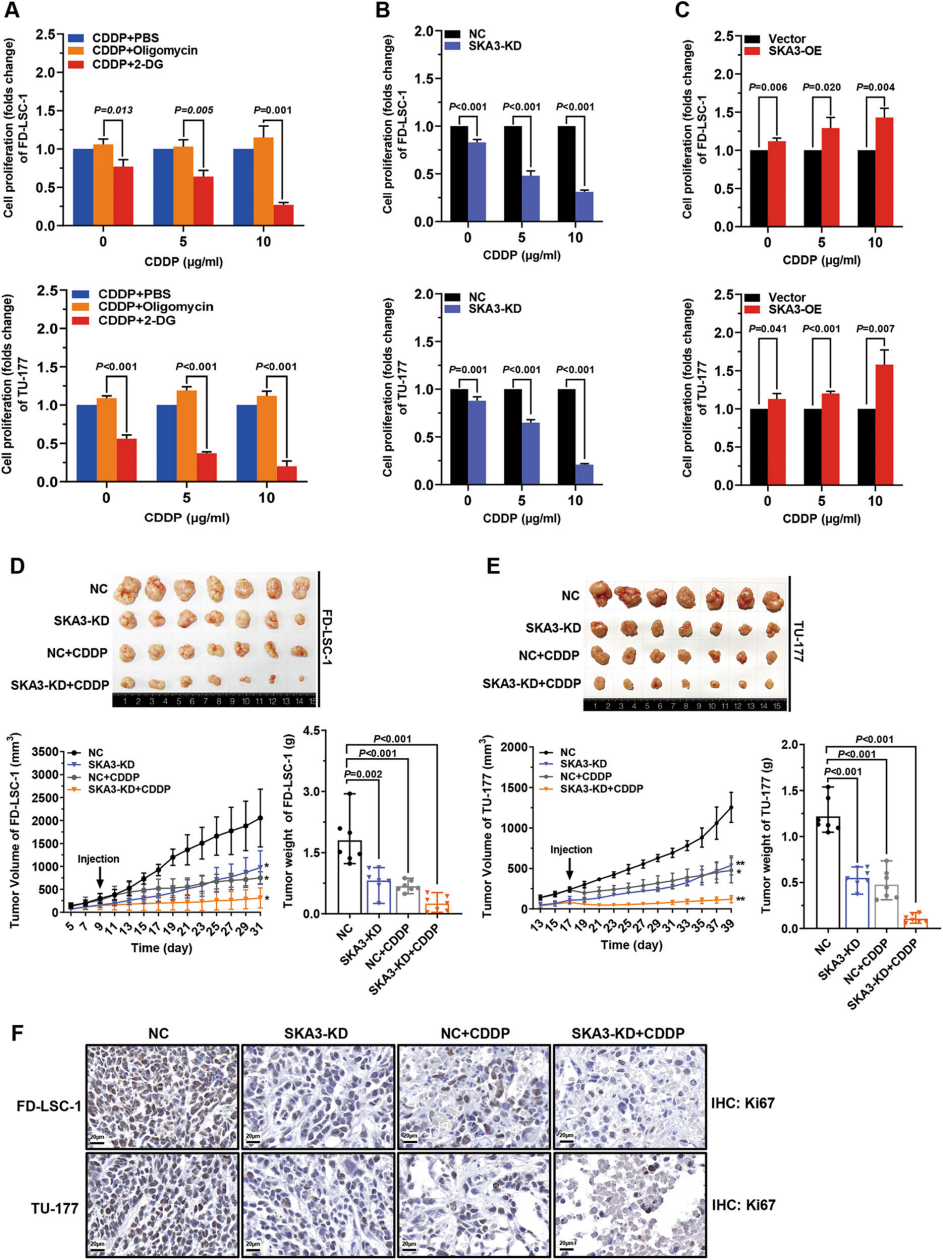
SKA3 protects laryngeal squamous cell carcinoma (LSCC) cells against the cytotoxic effects of cisplatin. **A** FD-LSC-1 and TU-177 cells were treated with different concentrations of CDDP, CDDP combined with oligomycin (CDDP + oligomycin), or CDDP combined with 2-DG (CDDP + 2-DG), respectively. Cell viability was determined using the CCK8 reagent after treatment for 24 h. **B**, **C** SKA3-knockdown (**B**) or SKA3-overexpressing (**C**) LSCC cells were treated with different concentrations of CDDP. Cell viability was detected using the CCK8 reagent after treatment for 24 h. **D**, **E** FD-LSC-1 (**D**) or TU-177 (**E**) cells with stable knockdown of SKA3 expression were subcutaneously injected into nude mice to establish a mouse xenograft model of LSCC. Tumor-bearing mice were divided into the following groups (*n* = 7 per group): negative-control group (NC), SKA3-knockdown group (SKA3-KD), negative control + CDDP-treatment group (NC + CDDP), and SKA3 knockdown + CDDP-treatment group (SKA3-KD + CDDP). CDDP (60 µg per mouse) was administered twice each week (intraperitoneal injection). The NC group received an intraperitoneal injection of an equal volume of normal saline twice per week. The mice were killed after 21 days of continuous treatment. The growth curves (left) and end-point weights (right) of tumors were plotted. **F** IHC analysis of the expression of Ki67 in tumor sections of FD-LSC-1 and TU-177 xenografts. Scale bar, 20 µm. The data are expressed as means ± SD of three independent experiments. *P < 0.05, **P < 0.001.

Insufficient efficacy of chemotherapy of the LSCC is the main cause of relapse and mortality, and high glycolytic activity contributes to the chemoresistance in malignant tumors^27,28^. To understand whether glycolysis contributed to the chemoresistance of LSCC cells, cells were treated with incremental doses of cisplatin (CDDP) alone or in combination with the glycolysis activator oligomycin or inhibitor 2-DG. As expected, oligomycin increased, whereas 2-DG reduced LSCC cell proliferation following CDDP treatment (Fig. 7A), demonstrating that accelerated glycolysis confers LSCC cell chemoresistance.

We then asked whether SKA3 regulated the sensitivity of LSCC cells to chemotherapy. The sensitivity of SKA3-knockdown and SKA3-overexpressing LSCC cells to CDDP was determined. CDDP treatment significantly decreased the proliferation of SKA3-knockdown FD-LSC-1 and TU-177 cells, indicating that SKA3 knockdown enhanced the sensitivity of LSCC cells to CDDP (Fig. 7B). By contrast, the proliferation of SKA3-overexpressing FD-LSC-1 and TU-177 cells was significantly higher than that of the vector group (Fig. 7C). Furthermore, we established a preclinical model of LSCC employing SKA3-knockdown cells and nude mice to evaluate the effect of SKA3 on the sensitivity to chemotherapy. Importantly, SKA3 knockdown sensitized xenografted tumors to CDDP treatment (Fig. 7D, E). This phenotype was closely associated with the relevant alterations in the proportion of Ki67-positive cells (Fig. 7F). Collectively, SKA3 confers LSCC resistance to chemotherapy via accelerating glycolysis in vitro and in vivo.

### c-Myc transcriptionally upregulates SKA3 in LSCC

**Fig. 8:**
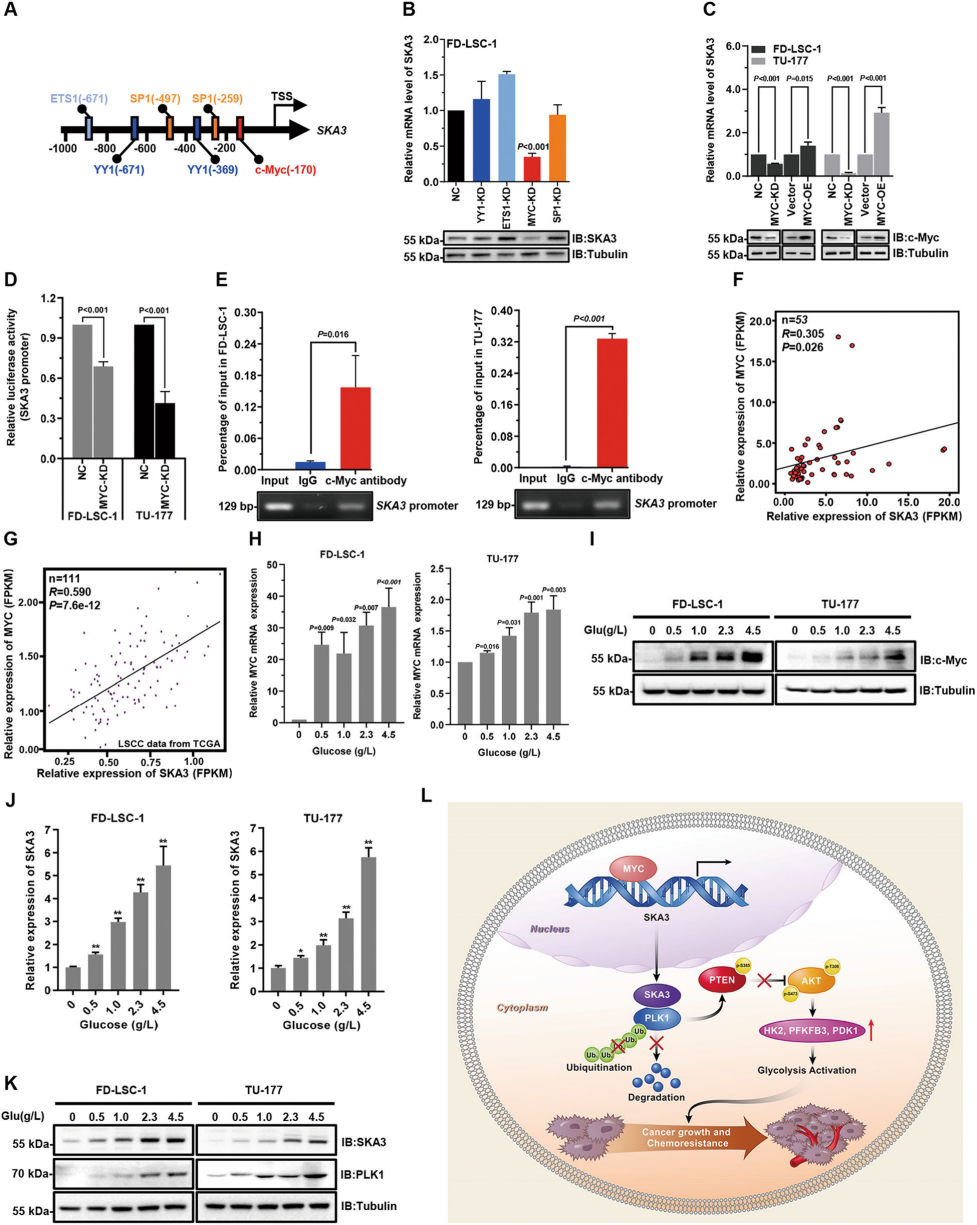
c-Myc transcriptionally regulates SKA3 expression, thus upregulating the PLK1 level in laryngeal squamous cell carcinoma (LSCC). **A** Diagram of transcription-factor-binding sites 1000 bp upstream of the SKA3 transcription start site predicted using EPD (https://epd.vital-it.ch/). **B** FD-LSC-1 cells were transfected with siRNAs targeting YY1, ETS1, MYC, SP1, or negative control for 48 h, and the expression levels of *SKA3* mRNA (upper) and protein (lower) were determined using qPCR and western blot analysis. **C** FD-LSC-1 and TU-177 cells were transfected with siRNA or a MYC-overexpression plasmid for 48 h; then the *SKA3* mRNA levels (upper) and protein (lower) were determined using qPCR and western blot analysis. **D** FD-LSC-1 and TU-177 cells were cotransfected with the SKA3-promoter reporter plasmid pGL4.10-SKA3 and an siRNA- targeting MYC, or with a negative control. Luciferase reporter assays were performed 48 h after transfection. **E** LSCC cells were subjected to chromatin immunoprecipitation using a c-Myc antibody. The enrichment level of SKA3-promoter sequences was analyzed using qPCR. qPCR products were verified using 2% agarose electrophoresis. **F** Expression correlation analysis of *SKA3* and *MYC* mRNAs using the transcriptome-sequencing data of 53 LSCC samples. Workflow type: HTSeq–FPKM. Significance was determined using Pearson’s rank-correlation test (*n* = 53, expression level represented by FPKM). **G** Expression correlation analysis of *SKA3* with *MYC* mRNA using the transcriptome-sequencing data of LSCC samples from the TCGA database (*n* = 111, expression level represented by FPKM). **H** qPCR analysis of *MYC* in FD-LSC-1 and TU-177 cells cultured in media containing 0, 0.5, 1, 2.3, or 4.5 g/L of glucose. **I** Western blot analysis of the c-Myc level in FD-LSC-1 and TU-177 cells cultured in media containing 0, 0.5, 1, 2.3, and 4.5 g/L of glucose. **J** qPCR analysis of *SKA3* in FD-LSC-1 and TU-177 cells cultured in media containing 0, 0.5, 1, 2.3, or 4.5 g/L of glucose. **K** Western blot analysis of the SKA3 and PLK1 levels in cells cultured in media containing 0, 0.5, 1, 2.3, and 4.5 g/L of glucose. **L** Schematic representation of a model for the major molecular mechanisms of “SKA3-PLK1-AKT” axis-promoted glycolysis, malignant progression, and chemoresistance in LSCC. The data are represented as means ± SD of three independent experiments. *P < 0.05, **P < 0.001.

To investigate the mechanism(s) responsible for the upregulation of SKA3 in LSCC, transcription factor-binding sites within the SKA3 promoter were predicted using bioinformatics (https://epd.vital-it.ch). This analysis identified consensus-binding sites of transcription factors c-Myc, YY1, EST1, and SP1 (Fig. 8A). However, knockdown of c-Myc but not the other three transcription factors significantly reduced SKA3 mRNA and protein levels in FD-LSC-1 cells (Fig. 8B). In contrast, overexpression of c-Myc increased the levels of SKA3, indicating the role of c-Myc in regulating SKA3 expression (Fig. 8C).

Luciferase reporter assays showed that knockdown of MYC reduced the transcriptional activity of the *SKA3* promoter in FD-LSC-1 and TU-177 cells (Fig. 8D), and chromatin immunoprecipitation (ChIP) assays confirmed that c-Myc binds to the *SKA3*-promoter region (Fig. 8E). Of note, the positive correlation between the levels of c-Myc and SKA3 was identified in our LSCC clinical samples along with data from the TCGA database (Fig. 8F, G). Since SKA3 and PLK1 were involved in glycolytic metabolism, we wondered whether c-Myc is responsive to changes in glucose levels. Thus, we examined the mRNA and protein expression of c-Myc in LSCC cells cultured with different concentrations of glucose. The data showed that c-Myc levels were upregulated in a glucose dose-dependent manner (Fig. 8H, I). Besides, SKA3 mRNA and protein levels along with PLK1 protein levels were also upregulated according to the increase in glucose concentration (Fig. 8J, K). These data suggest that glucose stimulates transcription factor c-Myc expression, thus transcriptionally upregulating SKA3 and maintaining high levels of PLK1 in LSCC.

## Discussion

As an important component of the spindle and kinetochore-related complexes^29^, SKA3 is essential for regulating mitosis with NDC80 complex and thus controlling cell proliferation and apoptosis^26,30^. Aberrant expression of SKA3 is closely associated with tumorigenesis and development^14,30^. For instance, SKA3 binds EGFR and consequently activates PI3K–AKT, thus promoting lung adenocarcinoma metastasis^14^. However, although EGFR mutation is rare in LSCC, SKA3-mediated AKT activation mainly depends on the serine/threonine-protein kinase PLK1 as silencing of PLK1 significantly attenuated the increase in p-AKT caused by SKA3 overexpression (Fig. 4F), indicating the context-dependent regulation of AKT.

Accordingly, as a key regulator of the cell cycle, PLK1 is also overexpressed in various cancer types and functions in tumor initiation, progression, and chemoresistance^31^. Blocking PLK1 seems a promising cancer-therapeutic strategy via arresting cells in mitosis^31^; however, PLK1 inhibitors do not obtain satisfactory results in clinical trials, which is probably associated with complex regulatory mechanisms of PLK1 in various types of cancer^20,32,33^. Here, we showed that SKA3, as a novel PLK1 regulator in LSCC, bound and protected PLK1 protein from ubiquitination-mediated degradation. Moreover, PLK1 protein stabilized by SKA3 overexpression significantly promoted glycolytic flux as evidenced by the increase in the extracellular acidification rate. Collectively, the interdependence between SKA3 and PLK1 determined the activation of AKT signaling and consequent glycolysis in LSCC cells.

The glycolytic pathway, which comprises glucose transporters and a series of kinases, such as hexokinase (HK), phosphofructokinase (PFK), and pyruvate kinase (PK), consumes glucose and produces ATP and lactate, providing energy for tumor cells and creating a microenvironment that facilitates the expression of malignant phenotypes, including proliferation, invasion, metastasis, and chemoresistance^34–36^. Also, high levels of glycolysis in laryngeal cancer significantly correlate with poor prognosis and chemoresistance^37^. Notably, our data demonstrated that the SKA3–PLK1–AKT axis selectively upregulated the expression of glycolytic enzymes HK2, PFKFB3, and PDK1, which in turn enhanced glycolytic levels of LSCC cells. Of note, the essentiality of PLK1–AKT–HK2/PFKFB3/PDK1 signaling in SKA3-mediated glycolysis was also confirmed in Ska3-knockout mouse embryos. Chemotherapy resistance considerably restricts the success of clinical treatments for LSCC^38^. In vivo and in vitro studies implied that inhibition of glycolysis enhanced the sensitivity of LSCC cells to the first-line chemotherapeutic drug cisplatin^39–41^. Of note, our experiments on cells and preclinical models showed that knockdown of SKA3 sensitized LSCC to cisplatin, indicating that SKA3 promotes chemoresistance through regulating glycolysis.

The SKA3 protein consists of the N- and C-terminal domain^42^. We found that deletion of the C-terminal domain abolished the interaction between SKA3 and PLK1, and subsequently decreased PLK1-expression levels, AKT phosphorylation, glycolytic activities, and cell proliferation, indicating that the C-terminal domain is essential for the function of SKA3 in LSCC cells. A recent study showed that phosphorylation of Thr360 at SKA3 protein promoted its binding to Ndc80 to maintain normal mitotic processes^26^. Our data show that phosphorylation at Thr360 is also important for the binding of SKA3 to PLK1. It also strongly supports the conclusion that phosphorylation of SKA3 at Thr360 is the main contributor to the high glycolytic activities that are associated with the malignant phenotype of LSCC cells.

The pro-oncoprotein c-Myc is upregulated in 50–60% of tumors and functioned in both initiation and progression of the tumor^43,44^. However, the functional roles of c-Myc in LSCC are not fully understood. Our study indicated that c-Myc may promote LSCC cell glycolysis via transcriptional regulation of SKA3 expression. Furthermore, we found that glucose stimulates c-Myc, SKA3, and PLK1 expression in a dose-dependent manner. Taken together, we speculate that c-Myc transcriptionally upregulates SKA3, which binds to PLK1 protein and further activates AKT signaling. Consequently, activation of the SKA3–PLK1–AKT axis enhances glycolytic activity via upregulating the expression of three glycolytic enzymes. Functionally, the activated glycolysis facilitates malignant progression and chemoresistance of LSCC (Fig. 8L). However, in normal cells, lowly expressed c-Myc accompanies with low levels of SKA3 and PLK1, indicating that the SKA3–PLK1–AKT axis may represent tumor-specific characteristics. Notably, to better understand whether SKA3 protein expression is a potential prognostic marker for platinum-based chemotherapy, the exploration of the relationship between SKA3 protein levels and clinical outcomes of LSCC patients receiving chemotherapy is required in future.

In summary, our data support a model that *SKA3* expression is transactivated by c-Myc in LSCC cells, and dysregulated overexpression of SKA3 interacts with PLK1 to activate the AKT signaling pathway to upregulate glycolysis level, providing an energy source to drive malignant proliferation and chemoresistance. Importantly, high SKA3 expression is associated with malignant progression and poor prognosis of patients with LSCC. Therefore, these findings indicate that SKA3 will serve as a novel potential biomarker for the diagnosis of LSCC, and the combination of SKA3 knockdown and PLK1 inhibition may be a novel strategy for LSCC therapy.

## Supplementary information

Supplementary Materials and Methods

Supplementary Figure Legends

Figure S1

Figure S2

Figure S3

Figure S4

Figure S5

Figure S6

Figure S7

Supplementary Table S1-S9

## Data Availability

GEPIA2 is an interactive web server for analyzing the RNA-sequencing expression data of tumors and normal samples from the TCGA and the GTEx projects (http://gepia2.cancer-pku.cn)^21^. ImageJ software is a public domain Java image-processing program inspired by NIH Image for the Macintosh (https://imagej.nih.gov/ij/). EPD is a collection of eukaryotic promoters derived from published articles (https://epd.vital-it.ch/)^22^. The transcriptome sequencing of 53 pairs of LSCC and ANM tissues are deposited at the Gene Expression Omnibus database with the accession number GSE142083. RNA-sequencing data of SKA3-knockdown LSCC cells are deposited at the Gene Expression Omnibus database with the accession number GSE128133. The authors declare that all data supporting the findings of this study are available within the paper and its supplementary information files.
