# Supplementary material for: Targeting SKA3 suppresses the proliferation and chemoresistance of laryngeal squamous cell carcinoma via impairing PLK1–AKT axis-mediated glycolysis
Source: Cell Death Dis. 2020 Oct 26;11(10):919. doi: 10.1038/s41419-020-03104-6 (PMC7589524; doi:10.1038/s41419-020-03104-6)
Supplement: Supplementary file 1 — Supplementary Materials and Methods [file 41419_2020_3104_MOESM1_ESM.docx]

**Targeting SKA3 suppresses the proliferation and chemoresistance of laryngeal squamous cell carcinoma via impairing PLK1-AKT axis-mediated glycolysis**

Wei Gao^1,2,3,4,5^, Yuliang Zhang^1,2^, Hongjie Luo^1^, Min Niu^1,2^, Xiwang Zheng^1,2^, Wanglai Hu^6^, Jiajia Cui^1,2^, Xuting Xue^1,2^, Yunfeng Bo^7^, Fengsheng Dai^1,3^, Yan Lu^8^, Dongli Yang^1,3^, Yujia Guo^1,2^, Huina Guo^1,2^, Huizheng Li^9^, Yu Zhang^10,4^, Tao Yang^11^, Li Li^5^, Linshi Zhang^12^, Rui Hou^13^, Shuxin Wen^14,2,🖂^, Changming An^15,🖂^, Teng Ma^16,🖂^, Lei Jin^17,🖂^, Wei Xu^18,19,20,🖂^ and Yongyan Wu^1,2,3,4,11,🖂^

**🖂 Correspondence should be addressed to:**

**Yongyan Wu**, wuyongyan@sxent.org, ORCID: 0000-0003-1669-3860

**Wei Xu**, xuwhns@126.com, ORCID: 0000-0002-9977-7535

**Lei Jin**, lei.jin@newcastle.edu.au, ORCID: 0000-0001-7187-9671

**Teng Ma**, mateng82913@163.com, ORCID: 0000-0002-8360-1543

**Changming An**, anchangming@cicams.ac.cn, ORCID: 0000-0002-8353-4547

**Shuxin Wen,** wensxsx@163.com, ORCID: 0000-0002-8377-2481

**Supplementary Materials and Methods:**

**Transcriptome sequencing analysis**

Total RNA was extracted from 53 pairs LSCC/matched ANM tissues, and LSCC cells using Trizol reagent (ThermoFisher Scientific). RNA integrity was examined with the Bioanalyzer 2100 (Agilent). High-quality RNA (RIN >7) samples were subjected to libraries construction, and then each library was sequenced on an Illumina HiSeq 4000 platform following the standard procedures by Novogene (Beijing, China).

**Cell culture**

HEK293T (CCTCC, Wuhan, China), human normal lung fibroblast cell line MRC-5 (CCTCC, Wuhan, China), and human LSCC cell line TU-177 (Bioleaf Biotech Co., Shanghai, China) were maintained in Dulbecco’s Modified Eagle Medium supplemented with 10% FBS (BI, Cromwell, CT, USA). Human LSCC cell line FD-LSC-1 (a gift from Professor Liang Zhou^1^) was cultured in BEGM™ Bronchial Epithelial Cell Growth Medium (Lonza, Walkersville, MD, USA) supplemented with 10% FBS (BI, Cromwell, CT, USA). The complete medium containing 1% penicillin/streptomycin (Solarbio, Beijing, China). All cells were maintained in a humidified atmosphere of 5% CO_2_ and 95% air at 37°C. Cell lines were authenticated by short tandem repeat analysis and tested for mycoplasma contamination using TransDetect PCR Mycoplasma Detection Kit (TransGen Biotech, Beijing, China).

**Generation of stably overexpressing and knockdown cells**

To generate FD-LSC-1 and TU-177 cells stably overexpressing wild type SKA3, mutant SKA3 and shRNA targeting specific gene, lentiviruses were produced in HEK293T cells by cotransfection with lentiviral plasmid and packaging plasmids GAG and VSVG. Virus supernatant was harvested 48 h after transfection and mixed with polybrene (8 μg/ml) to increase the infection efficiency, and then added into FD-LSC-1 or TU-177 cells. After 48 h incubation, 2 μg/ml puromycin (Santa Cruz) was added for 1 week to screen stable cells. To obtain FD-LSC-1 and TU-177 cells stably knockdown of SKA3, plasmid carrying sgRNA and Cas9, and donor plasmid containing homologous arm sequence flanking the target site were delivered into cells using Lipofectamine 3000 reagent (ThermoFisher Scientific), and stable cells were selected with 2 μg/ml puromycin (Santa Cruz) for 10 days.

**Immunofluorescence staining**

FD-LSC-1 and TU-177 cells cultured on cover slides were cotransfected with Flag-tagged SKA3 and HA-tagged PLK1 plasmids for 48 h. Cells were washed twice with PBS, then fixation and permeabilization with immunostaining fixation buffer (Beyotime, Shanghai, China) for 20 min, and the cells were blocked with blocking buffer (Beyotime) for 2 h. Cells were incubated with primary antibodies against Flag and HA overnight at 4 °C. After 3 washes with washing buffer, the cells were incubated with Alexa Fluor 488/555-conjugated secondary antibodies (Beyotime) for 2 h at room temperature. Nuclei were counterstained with DAPI followed by 3 washes with washing buffer. Results were observed and captured under the Leica TCS SP8 confocal laser scanning microscope (Leica Microsystems Inc., Buffalo Grove, IL).

**Antibodies and reagents**

SKA3 antibody (rabbit, polyclonal, A304-215A) was purchased from Bethyl Laboratories, Inc. (Montgomery, TX). Antibodies against PFKFB3 (rabbit, monoclonal, 13123s), PDK1 (rabbit, polyclonal, 3062S), c-Myc (rabbit, polyclonal, 9402S), PLK1 (rabbit, monoclonal, 4513S), phospho-PLK1 (rabbit, monoclonal, Thr210, 9062s), PTEN (rabbit, monoclonal, 9188S), phospho-PTEN (rabbit, monoclonal, Ser380/Thr382/383, 9549S), AKT (rabbit, polyclonal, 9272S), phospho-AKT (rabbit, monoclonal, Thr308, 2965S), phospho-AKT (rabbit, monoclonal, Ser473, 4060S) and HK2 (rabbit, monoclonal, 2867S) were purchased from Cell Signaling Technology (Danvers, MA). Antibody against Tubulin (mouse, monoclonal, HC101) was purchased from TransGen Biotech (Beijing, China). SKA3 rabbit polyclonal for immunochemical staining was purchased from Merck (HPA039272, Darmstadt, Germany). HA antibody (rabbit, monoclonal, AF2305) was purchased from Beyotime (Nanjing, China). Flag antibody (mouse, monoclonal, F1804) was purchased from Sigma (St. Louis, MO). Cycloheximide (CHX) (Cat# sc-3508B) was purchased from Santa Cruz Biotechnology, Inc. (Dallas, TX). MG132 (Cat# M7449) was purchased from Sigma (St. Louis, MO). Cisplatin (Cat# S1166), BI2536 (Cat# S1109), and MK-2206 (Cat# S1078) were purchased from Selleck (Houston, TX).

**Cell proliferation analysis**

Cell proliferation was measured using the iCelligence system (ACEA Biosciences Inc., San Diego, USA). Briefly, 24 h after transfection, cells were seeded in E-plate 16 PET (5x10^3^ cells/well), and cellular index (value derived from electrical impedance that reflects the number of living cells) was measured every 1 hour for 48 hours. Moreover, EdU staining was performed to assess cell proliferation ability by using the Cell-Light EdU imaging kit (RiboBio Co., Guangzhou, China) following the manufacturer’s instructions.

**Colony formation assay**

For colony formation assay, cells were seeded in a 6-well plate (800 cells/well) and cultured for 10 days. Cells were rinsed with PBS, fixed with 4% paraformaldehyde for 15 min and stained with 0.1% crystal violet for 10 min, wash 3 times with PBS, then air-dried.

**RNA extraction, reverse transcription, and real-time quantitative PCR (qPCR)**

Total RNA was extracted from frozen tissues or cells using TRIzol reagent (ThermoFisher Scientific). Reverse transcription was performed using the HiScript II 1st Strand cDNA Synthesis Kit (Vazyme, Nanjing, China) with 1 μg total RNA. qPCR amplification was performed using ChamQ SYBR qPCR Master Mix (Vazyme) on a 7500 FAST real-time PCR system (Applied Biosystems, Foster City, CA). The reaction conditions for qPCR was 95°C for 30 sec, followed by 40 cycles of 95°C for 10 sec and 60°C for 30 sec. The specificity of the primer was tested by melting curve analysis. The comparative Ct method was used for quantifying target mRNA expression normalized to that of 18S rRNA and relative to the calibrator. The qPCR primers were listed in **Table S8**.

**Western blotting**

Tissues or cells were lysed with RIPA buffer containing Protease and Phosphatase Inhibitor Cocktail (Pierce, Rockford, IL). Protein concentrations were determined using BCA Protein Assay Reagent (Pierce). Cell lysates or CoIP elutes was separated by SDS-PAGE, and transferred to Immobilon-P Transfer Membranes (Millipore, Billerica, MA) by wet transfer. The membranes were blocked with 5% nonfat milk for 2 h at room temperature, incubated with primary antibody overnight at 4°C. After 3 washes with TBST, membranes were incubated with horseradish peroxidase (HRP)-conjugated secondary antibody (Beyotime, Nanjing, China) at room temperature for 2 h, and then washed 3 times with TBST. Membranes were detected using the Western Bright ECL HRP substrate (Advansta Inc., San Jose, CA). The relative protein amount was quantified by densitometry using ImageJ software.

**Immunohistochemical staining and analysis**

The immunohistochemical staining (IHC) was performed on tissue microarrays (TMAs), which were fixed in formalin, paraffin-embedded and cut into 5 μm and 1.5 mm in diameter sheets and contained 165 cases of LSCC and ANM arrays stored in the biobank of First Hospital of Shanxi Medical University (**Table S9**). TMA sections were dewaxed and re-hydrated in descending concentrations of ethanol (100%, 90%, 80%, 70%). Samples were processed in an autoclave with sodium citrate for antigen retrieval. Endogenous peroxidase activity was blocked by immersing tissue sections in 3% H_2_O_2_ in methanol (v/v) at room temperature for 10 min. Nonspecific staining was reduced by incubating sections with normal nonimmune serum (Boster Co., Hubei, China) for 15 min at room temperature. The sections were incubated with primary antibody overnight at 4°C in a moist chamber, and washed 3 times with PBST (containing PBS and 1‰ Tween20). Sections were incubated with HRP-conjugated secondary antibody (MaxinBio, Fuzhou, China) for 15 min at room temperature, then washed 3 times for with PBST. Diaminobenzidine (Dako, Glostrup, Denmark) was used as a chromogen and hematoxylin for counterstaining. Then, sections were dehydrated and mounted with coverslips. The entire TMA sections were scanned on Panoramic slide scanner II (3D HISTECH, Budapest, Hungary) and quantified with Case Viewer soft version 2.3 (3D HISTECH, Budapest, Hungary) to obtain the IHC score of each dot by two independent pathologists with agreements, but adjuster when argued. The slides were coded and pathologists were blinded for the information of LSCC patients.in order to avoid the observer bias. The section with IHC score above the median value was defined as high expression, and vice versa.

**Chromatin Immunoprecipitation (ChIP)**

ChIP was performed using the EZ-ChIP Kit (Millipore, Darmstadt, Germany) according to the manufacturer’s instructions. Briefly, cells were cross-linked with 37% formaldehyde (final concentration 1% in culture media), pelleted, and resuspended in SDS lysis buffer containing 1X protease inhibitor cocktail. The cells were sonicated and centrifuged to remove the insoluble material. The supernatants were collected and incubated with indicated antibody and Protein G agarose beads overnight at 4°C with rotation. The beads were washed, and the precipitated chromatin complexes were collected, purified, and de-crosslinked at 65°C for 4 h, followed by RNase and proteinase treatment, and then DNA was purified using spin columns. The resulting DNA fragments were quantified.

**References**

1. Wu, C. P. et al. Establishment and characterization of a novel HPV-negative laryngeal squamous cell carcinoma cell line, FD-LSC-1, with missense and nonsense mutations of TP53 in the DNA-binding domain. *Cancer Lett.* **342**, 92-103 (2014).
