# Supplementary material for: Targeting SKA3 suppresses the proliferation and chemoresistance of laryngeal squamous cell carcinoma via impairing PLK1–AKT axis-mediated glycolysis
Source: Cell Death Dis. 2020 Oct 26;11(10):919. doi: 10.1038/s41419-020-03104-6 (PMC7589524; doi:10.1038/s41419-020-03104-6)
Supplement: Supplementary file 2 — Supplementary Figure Legends [file 41419_2020_3104_MOESM2_ESM.docx]

**Targeting SKA3 suppresses the proliferation and chemoresistance of laryngeal squamous cell carcinoma via impairing PLK1-AKT axis-mediated glycolysis**

Wei Gao^1,2,3,4,5^, Yuliang Zhang^1,2^, Hongjie Luo^1^, Min Niu^1,2^, Xiwang Zheng^1,2^, Wanglai Hu^6^, Jiajia Cui^1,2^, Xuting Xue^1,2^, Yunfeng Bo^7^, Fengsheng Dai^1,3^, Yan Lu^8^, Dongli Yang^1,3^, Yujia Guo^1,2^, Huina Guo^1,2^, Huizheng Li^9^, Yu Zhang^10,4^, Tao Yang^11^, Li Li^5^, Linshi Zhang^12^, Rui Hou^13^, Shuxin Wen^14,2,🖂^, Changming An^15,🖂^, Teng Ma^16,🖂^, Lei Jin^17,🖂^, Wei Xu^18,19,20,🖂^ and Yongyan Wu^1,2,3,4,11,🖂^

**🖂 Correspondence should be addressed to:**

**Yongyan Wu**, wuyongyan@sxent.org, ORCID: 0000-0003-1669-3860

**Wei Xu**, xuwhns@126.com, ORCID: 0000-0002-9977-7535

**Lei Jin**, lei.jin@newcastle.edu.au, ORCID: 0000-0001-7187-9671

**Teng Ma**, mateng82913@163.com, ORCID: 0000-0002-8360-1543

**Changming An**, anchangming@cicams.ac.cn, ORCID: 0000-0002-8353-4547

**Shuxin Wen,** wensxsx@163.com, ORCID: 0000-0002-8377-2481

**Supplementary Figure Legends**

**Figure S1. Effects of SKA3 on glycolysis in LSCC cells.** FD-LSC-1 and TU-177 cells were transfected with SKA3-overexpression plasmid (SKA3-OE), empty vector (Vector), siRNAs targeting SKA3 (SKA3-KD), or negative control siRNAs (NC) for 36 hr. Acidification of the culture medium was evaluated by visually inspecting the color of the medium.

**Figure S2. Effects of SKA3 on glycolytic genes expression in LSCC cells.** Knockdown or overexpression of SKA3 was performed in FD-LSC-1 and TU-177 LSCC cells for 48 h. The expression levels of GLUT2, HK1, GPI, PFKFB2, PFKP, ALDOA, PGK1, PGAM1, ENO1, PKM2, and LDHA were determined using western blot analysis.

**Figure S3. Generation of Ska3-knockout mice using CRISPR/Cas9 technology.** (A) Diagram of paired gRNA and validation primer for generation of Ska3-knockout mouse. (B) PCR-genotyping of Ska3-knockout transgenic mice during development. Male and female Ska3-knockout heterozygous mice were cohabitated, and then embryos were collected after 5.5, 7.5, 9.5, 12.5, 13.5, 14.5, and 16.5 days after cohabitation (dpc) (top). Results: Heterozygote, 1040 bp and 574 bp amplicons; wild-type, 574 bp amplicon (bottom). Representative images are shown.

**Figure S4. Enrichment of SKA3 binding proteins by Co-immunoprecipitation.** Cell lysates of TU-177 cells stably expressing Flag-SKA3 were immunoprecipitated using an anti-Flag antibody. The eluates were resolved using SDS-PAGE, and the gels were stained with Coomassie Brilliant Blue. The unique protein bands were excised from the gels and subjected to mass spectrometry. Representative gel image was showed.

**Figure S5. Knockdown of HK2, PFKFB3 or PDK1 reversed SKA3 stimulated aerobic glycolysis.** (A-B) SKA3-overexpressing FD-LSC-1 (A) and TU-177 (B) cells were transfected with siRNAs targeting HK2, PFKFB3, or PDK1 or with a negative control. After 48 h, glycolysis was measured using a Seahorse energy metabolism instrument (Seahorse XFP). Data represent the mean ± SD of three experiments.

**Figure S6. PLK1 expression level is positive correlated with SKA3 level in HNSCC single cell dataset.** (A) The plots show expression levels of SKA3 and PLK1 (log2TPM) in individual cells of four non-immune cell types. (B) The correlation between SKA3 and PLK1 expression (log2TPM) in same single cell. The data were derived from single-cell transcriptomic analysis of head and neck cancer by Puram et al. (Cell, 171(7), pp.1611-1624.).

**Figure S7. Expression verification of wild-type and domain deleted SKA3 expression plasmids.** FD-LSC-1 and TU-177 cells were transfected with SKA3-WT, SKA3-ΔN, SKA3-ΔC expression plasmid or an empty vector for 48 h, and the expression of SKA3 was detected by western blotting using antibody against Flag.
