# Supplementary figures and images for: Targeting SKA3 suppresses the proliferation and chemoresistance of laryngeal squamous cell carcinoma via impairing PLK1–AKT axis-mediated glycolysis
Source: Cell Death Dis. 2020 Oct 26;11(10):919. doi: 10.1038/s41419-020-03104-6 (PMC7589524; doi:10.1038/s41419-020-03104-6)

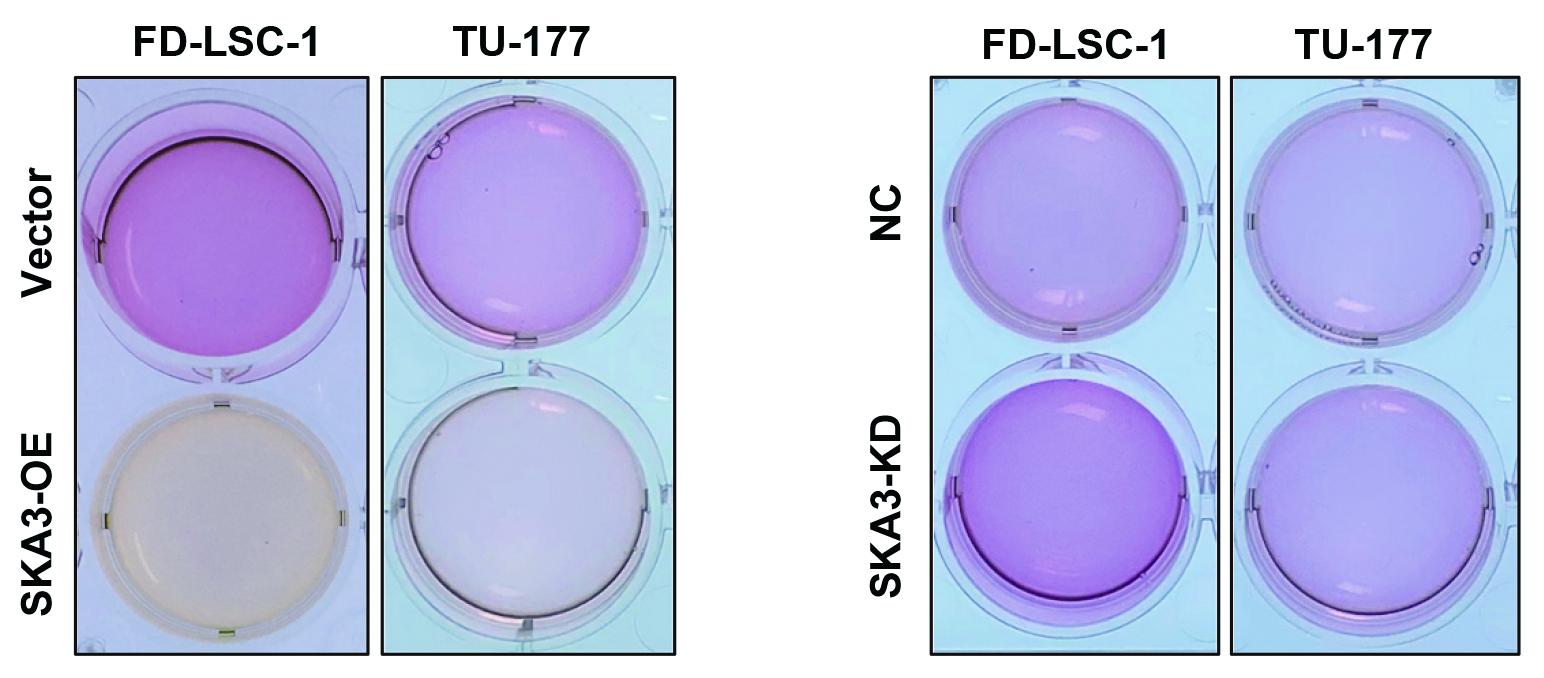

Supplement: Supplementary file 3 — Figure S1 [file 41419_2020_3104_MOESM3_ESM.tif]

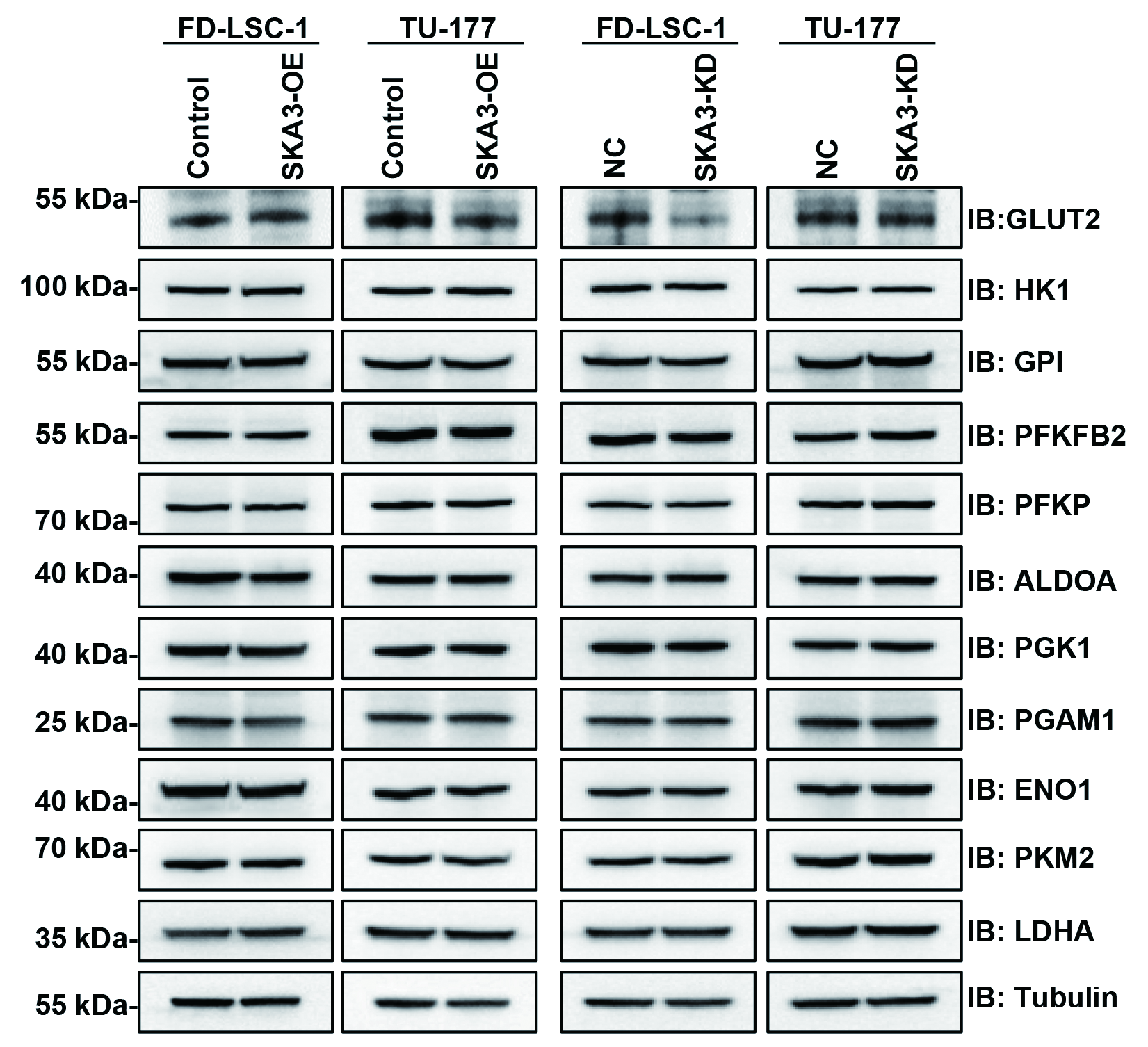

Supplement: Supplementary file 4 — Figure S2 [file 41419_2020_3104_MOESM4_ESM.tif]

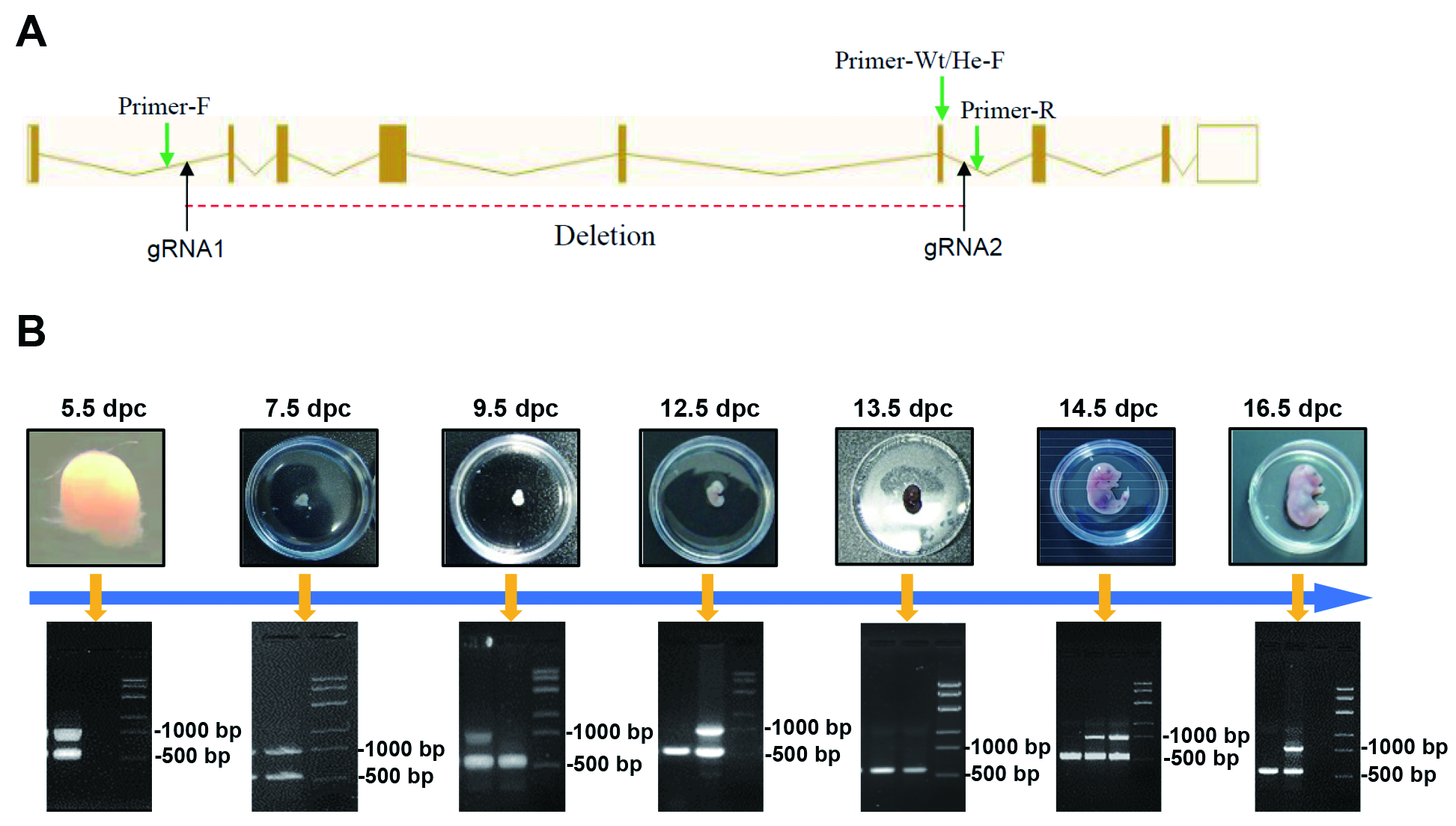

Supplement: Supplementary file 5 — Figure S3 [file 41419_2020_3104_MOESM5_ESM.tif]

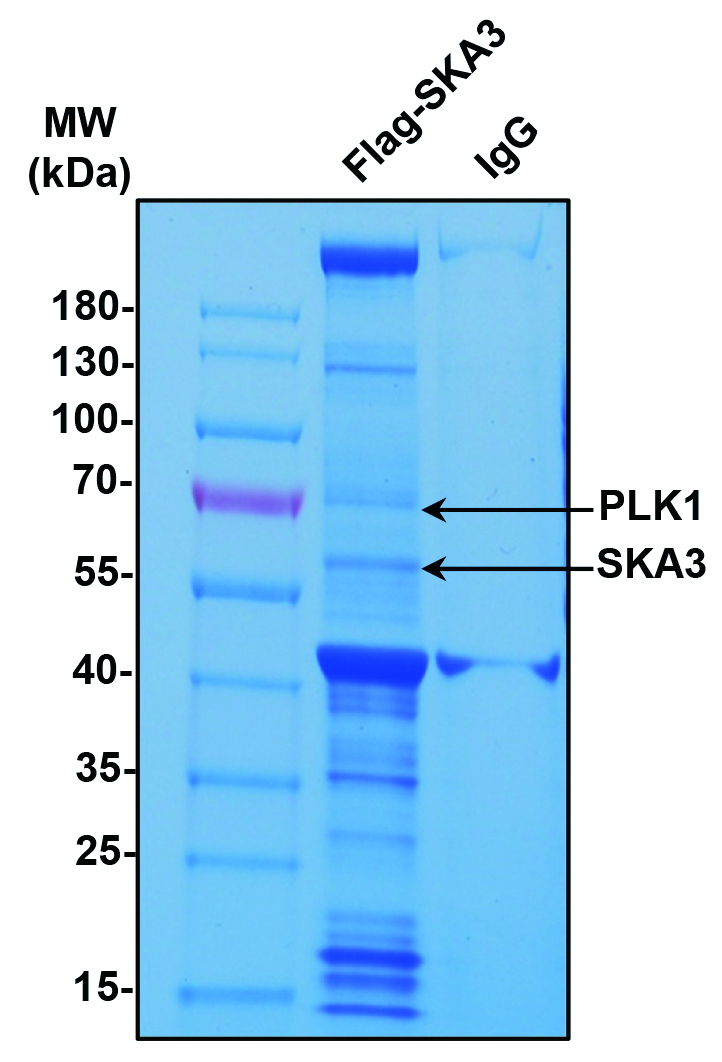

Supplement: Supplementary file 6 — Figure S4 [file 41419_2020_3104_MOESM6_ESM.tif]

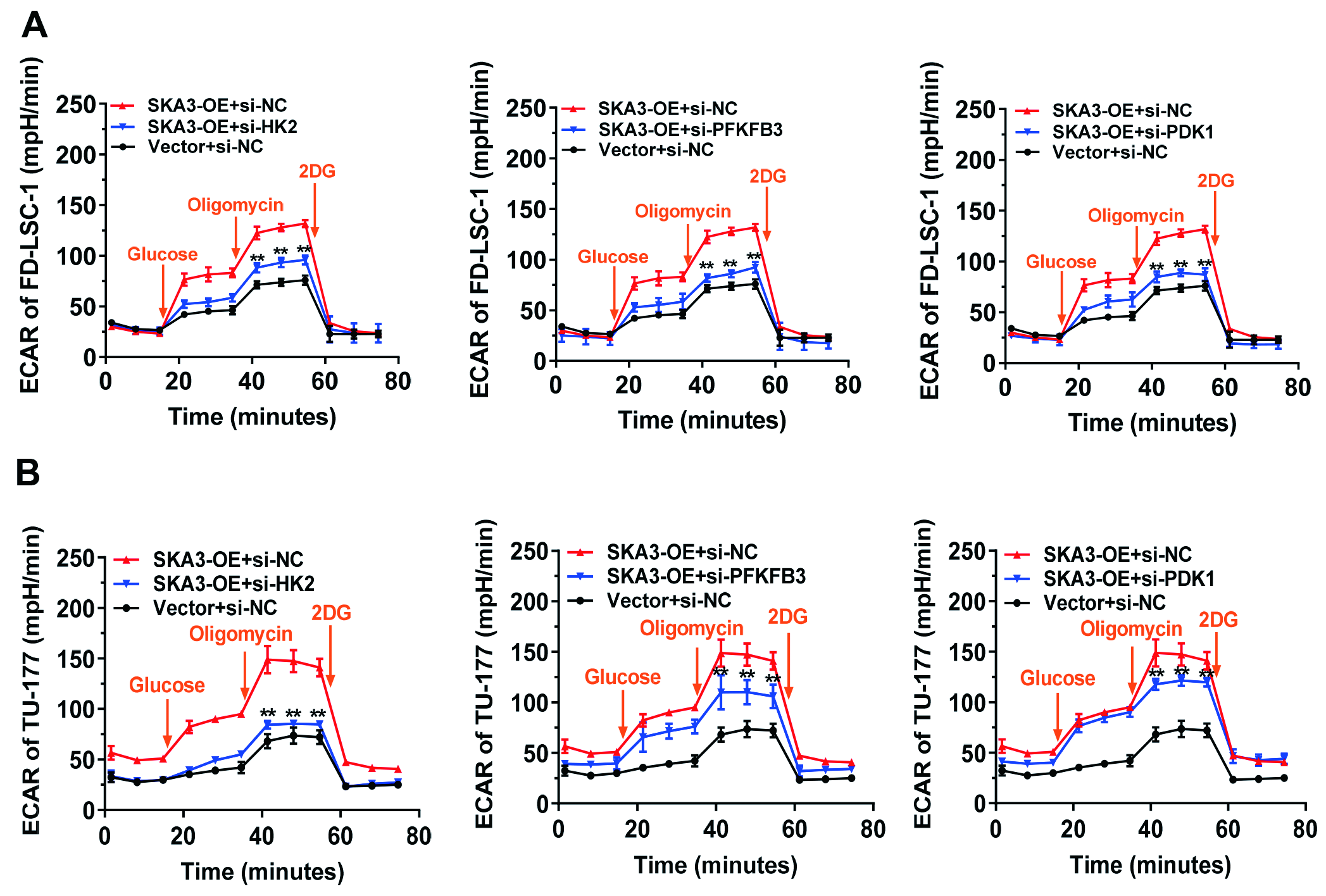

Supplement: Supplementary file 7 — Figure S5 [file 41419_2020_3104_MOESM7_ESM.tif]

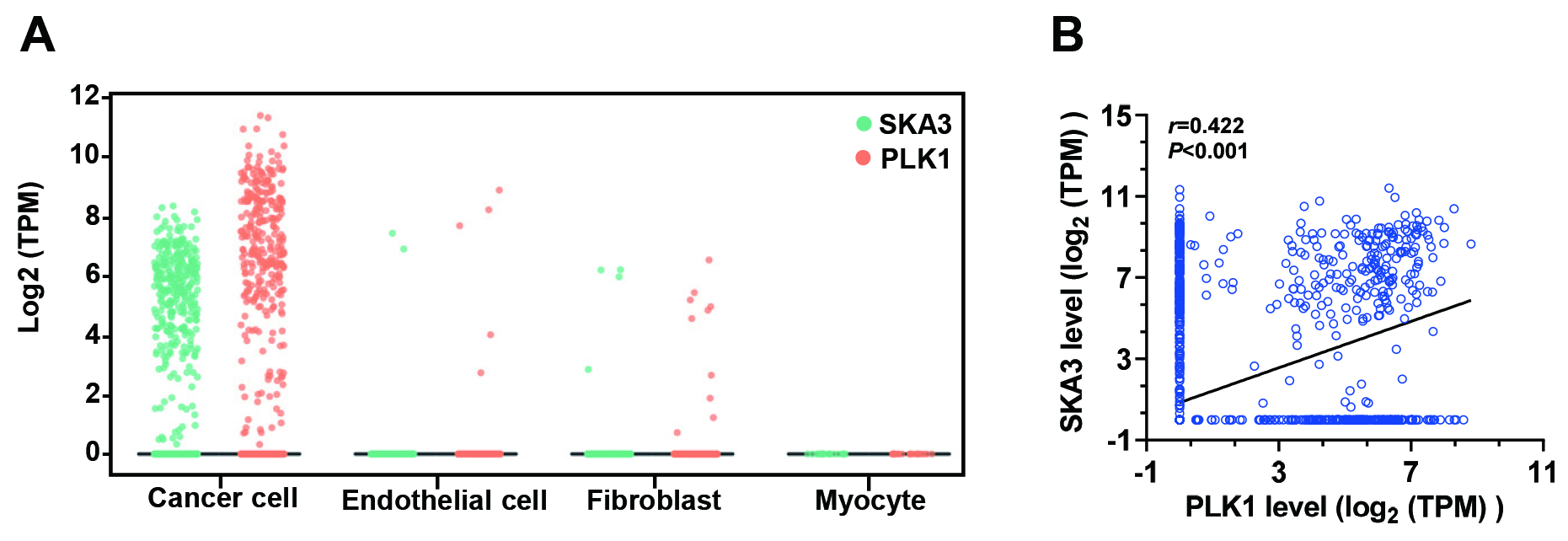

Supplement: Supplementary file 8 — Figure S6 [file 41419_2020_3104_MOESM8_ESM.tif]

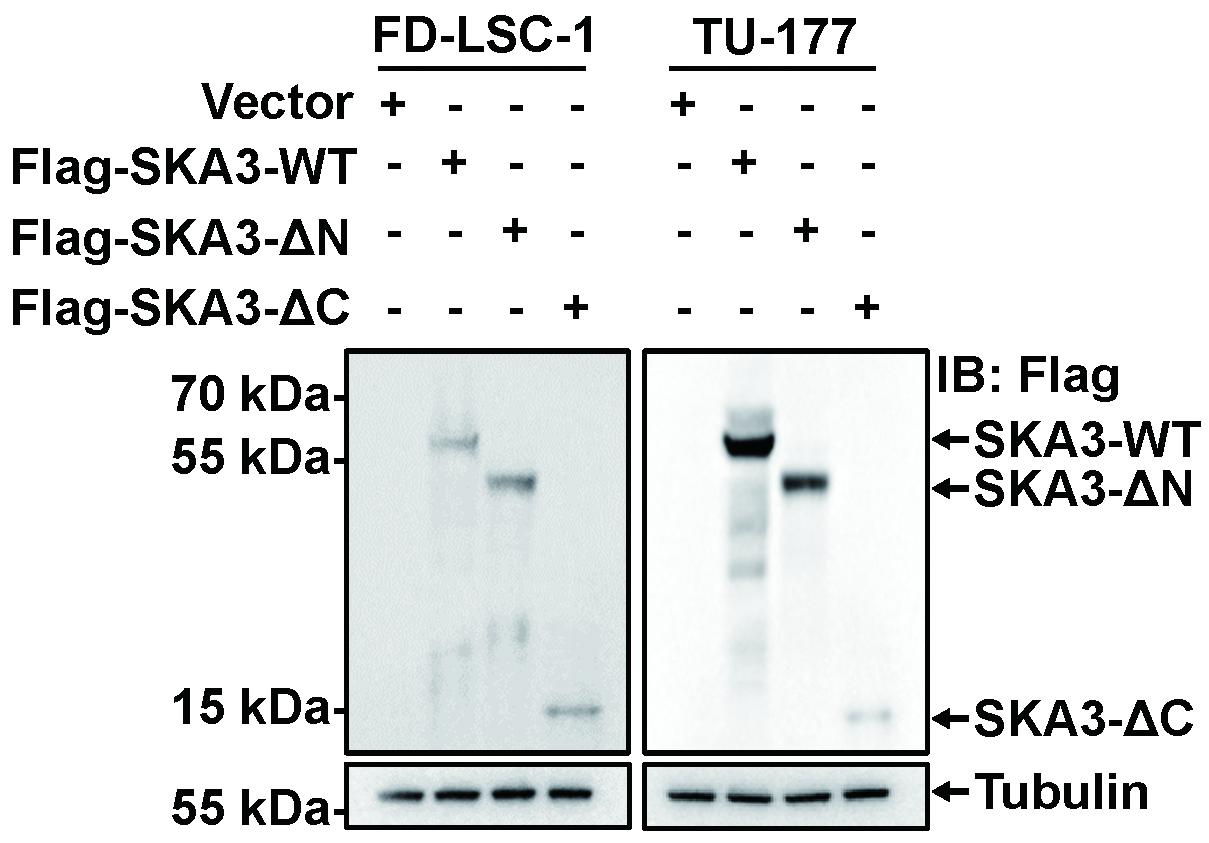

Supplement: Supplementary file 9 — Figure S7 [file 41419_2020_3104_MOESM9_ESM.tif]
