# Supplementary material for: Targeting SKA3 suppresses the proliferation and chemoresistance of laryngeal squamous cell carcinoma via impairing PLK1–AKT axis-mediated glycolysis
Source: Cell Death Dis. 2020 Oct 26;11(10):919. doi: 10.1038/s41419-020-03104-6 (PMC7589524; doi:10.1038/s41419-020-03104-6)
Supplement: Supplementary file 10 — Supplementary Table S1-S9 [file 41419_2020_3104_MOESM10_ESM.docx]

**Targeting SKA3 suppresses the proliferation and chemoresistance of laryngeal squamous cell carcinoma via impairing PLK1-AKT axis-mediated glycolysis**

Wei Gao^1,2,3,4,5^, Yuliang Zhang^1,2^, Hongjie Luo^1^, Min Niu^1,2^, Xiwang Zheng^1,2^, Wanglai Hu^6^, Jiajia Cui^1,2^, Xuting Xue^1,2^, Yunfeng Bo^7^, Fengsheng Dai^1,3^, Yan Lu^8^, Dongli Yang^1,3^, Yujia Guo^1,2^, Huina Guo^1,2^, Huizheng Li^9^, Yu Zhang^10,4^, Tao Yang^11^, Li Li^5^, Linshi Zhang^12^, Rui Hou^13^, Shuxin Wen^14,2,🖂^, Changming An^15,🖂^, Teng Ma^16,🖂^, Lei Jin^17,🖂^, Wei Xu^18,19,20,🖂^ and Yongyan Wu^1,2,3,4,11,🖂^

**🖂 Correspondence should be addressed to:**

**Yongyan Wu**, wuyongyan@sxent.org, ORCID: 0000-0003-1669-3860

**Wei Xu**, xuwhns@126.com, ORCID: 0000-0002-9977-7535

**Lei Jin**, lei.jin@newcastle.edu.au, ORCID: 0000-0001-7187-9671

**Teng Ma**, mateng82913@163.com, ORCID: 0000-0002-8360-1543

**Changming An**, anchangming@cicams.ac.cn, ORCID: 0000-0002-8353-4547

**Shuxin Wen,** wensxsx@163.com, ORCID: 0000-0002-8377-2481

**Supplementary Tables:**

**Table S1. Top 50 upregulated genes in LSCC tissues screened by transcriptome sequencing.**

| **Gene name** | **Gene type** | **Log2(fold change)** | **Absolute fold change** | **P value** | **P adj** |
| --- | --- | --- | --- | --- | --- |
| KRT17 | protein_coding | 3.27 | 9.67 | 1.01E-51 | 2.62E-49 |
| LAMC2 | protein_coding | 2.96 | 7.77 | 3.75E-55 | 1.34E-52 |
| COL1A1 | protein_coding | 2.86 | 7.28 | 6.42E-50 | 1.37E-47 |
| POSTN | protein_coding | 2.57 | 5.94 | 1.27E-35 | 7.01E-34 |
| GJA1 | protein_coding | 2.37 | 5.17 | 5.18E-35 | 2.73E-33 |
| PLAU | protein_coding | 2.36 | 5.14 | 1.02E-50 | 2.35E-48 |
| SLC16A1 | protein_coding | 2.23 | 4.69 | 1.24E-48 | 2.40E-46 |
| COL5A2 | protein_coding | 2.20 | 4.58 | 8.17E-44 | 1.01E-41 |
| COL4A1 | protein_coding | 2.15 | 4.45 | 6.93E-59 | 4.13E-56 |
| COL12A1 | protein_coding | 2.08 | 4.22 | 1.52E-48 | 2.89E-46 |
| FSCN1 | protein_coding | 2.01 | 4.02 | 8.41E-45 | 1.11E-42 |
| IFI27 | protein_coding | 1.95 | 3.87 | 2.23E-22 | 3.50E-21 |
| LUM | protein_coding | 1.94 | 3.85 | 9.51E-44 | 1.17E-41 |
| COL3A1 | protein_coding | 1.93 | 3.81 | 5.43E-25 | 1.12E-23 |
| FN1 | protein_coding | 1.92 | 3.79 | 4.00E-25 | 8.35E-24 |
| CDH3 | protein_coding | 1.90 | 3.72 | 3.31E-40 | 2.90E-38 |
| LAMA3 | protein_coding | 1.89 | 3.71 | 1.90E-39 | 1.53E-37 |
| CENPF | protein_coding | 1.89 | 3.71 | 3.20E-28 | 9.12E-27 |
| TOP2A | protein_coding | 1.89 | 3.70 | 1.79E-27 | 4.74E-26 |
| COL4A2 | protein_coding | 1.87 | 3.66 | 8.78E-48 | 1.50E-45 |
| ECT2 | protein_coding | 1.83 | 3.54 | 4.10E-52 | 1.13E-49 |
| COL5A1 | protein_coding | 1.82 | 3.53 | 1.74E-24 | 3.40E-23 |
| ODC1 | protein_coding | 1.82 | 3.52 | 5.68E-21 | 7.69E-20 |
| SLC2A1 | protein_coding | 1.80 | 3.47 | 9.29E-26 | 2.08E-24 |
| COL6A3 | protein_coding | 1.76 | 3.39 | 9.45E-27 | 2.34E-25 |
| SLC7A5 | protein_coding | 1.76 | 3.38 | 4.56E-24 | 8.56E-23 |
| SERPINH1 | protein_coding | 1.75 | 3.37 | 2.50E-47 | 4.08E-45 |
| GPNMB | protein_coding | 1.75 | 3.37 | 5.76E-23 | 9.60E-22 |
| COL7A1 | protein_coding | 1.74 | 3.34 | 6.13E-36 | 3.52E-34 |
| MYO1B | protein_coding | 1.73 | 3.32 | 1.22E-42 | 1.36E-40 |
| MCM2 | protein_coding | 1.73 | 3.31 | 3.95E-42 | 4.16E-40 |
| FOXM1 | protein_coding | 1.71 | 3.27 | 1.04E-43 | 1.27E-41 |
| COL17A1 | protein_coding | 1.68 | 3.21 | 1.33E-15 | 1.07E-14 |
| KPNA2 | protein_coding | 1.68 | 3.20 | 1.70E-40 | 1.56E-38 |
| MKI67 | protein_coding | 1.68 | 3.20 | 2.44E-19 | 2.85E-18 |
| PRC1 | protein_coding | 1.67 | 3.19 | 1.11E-32 | 4.73E-31 |
| COL1A2 | protein_coding | 1.65 | 3.14 | 5.34E-20 | 6.58E-19 |
| TGFBI | protein_coding | 1.60 | 3.03 | 1.38E-22 | 2.22E-21 |
| SPAG5 | protein_coding | 1.58 | 2.99 | 2.31E-36 | 1.37E-34 |
| PHLDB2 | protein_coding | 1.57 | 2.97 | 7.33E-30 | 2.42E-28 |
| TP63 | protein_coding | 1.57 | 2.96 | 3.19E-21 | 4.43E-20 |
| TNC | protein_coding | 1.56 | 2.95 | 4.04E-17 | 3.80E-16 |
| ARHGAP11A | protein_coding | 1.52 | 2.86 | 6.23E-24 | 1.15E-22 |
| SULF1 | protein_coding | 1.50 | 2.83 | 1.74E-17 | 1.70E-16 |
| FANCI | protein_coding | 1.49 | 2.82 | 5.84E-32 | 2.32E-30 |
| HIST1H2AL | protein_coding | 1.49 | 2.81 | 9.83E-21 | 1.30E-19 |
| SKA3 | protein_coding | 1.49 | 2.80 | 5.47E-23 | 9.16E-22 |
| ITGA6 | protein_coding | 1.48 | 2.79 | 2.89E-30 | 9.85E-29 |
| SPARC | protein_coding | 1.48 | 2.79 | 3.72E-21 | 5.14E-20 |
| CD276 | protein_coding | 1.47 | 2.78 | 2.49E-43 | 2.97E-41 |

**Table S2. Correlation between SKA3 expression and clinicopathological features in 165 LSCC patients.**

| **Characteristics** | **Number of Cases**  **(n)** | **SKA3 IHC Score**  **(Mean ± Std.)** | ***P* value** |
| --- | --- | --- | --- |
| **Age** |  |  |  |
| <60 | 80 | 78.32 ± 21.05 | 0.885 |
| ≥60 | 85 | 78.81 ± 22.15 |  |
| **Sex** |  |  |  |
| Female | 7 | 91.55 ± 13.39 | 0.104 |
| Male | 158 | 78.00 ± 21.70 |  |
| **Primary Site** |  |  |  |
| Glottic | 72 | 75.26 ± 22.00 | 0.189* |
| Supraglottic | 87 | 81.48 ± 21.42 |  |
| Subglottic | 6 | 76.27 ± 13.14 |  |
| **Differentiation** |  |  |  |
| Well | 44 | 73.08 ± 20.52 | 0.052* |
| Moderately | 88 | 78.88 ± 22.12 |  |
| Poorly | 33 | 85.08 ± 20.03 |  |
| **T Staging^1^** |  |  |  |
| T1 + T2 | 74 | 71.11 ± 23.31 | ***<0.001*** |
| T3 + T4 | 91 | 84.65 ± 17.98 |  |
| **Cervical lymph node metastasis** |  |  |  |
| N0 | 119 | 76.54 ± 22.33 | 0.051 |
| N+ | 46 | 83.83 ± 18.65 |  |
| **Distant metastasis** |  |  |  |
| M0 | 152 | 77.64 ± 21.25 | 0.056 |
| M1 | 13 | 89.52 ± 23.04 |  |
| **Clinical stage** |  |  |  |
| I + II | 64 | 68.00 ± 21.76 | ***<0.001*** |
| III + IV | 101 | 85.28 ± 18.63 |  |

^1^TNM Staging is referring to the 8th AJCC TNM Staging Criteria.

^*^The data were analyzed by using one-way ANOVA.

**Table S3. Differentially expressed gene in SKA3 knockdown LSCC cells.**

| **ID** | **Gene name** | **Log2 Fold Change** | **Fold change** | **p value** | **Regulation trend** |
| --- | --- | --- | --- | --- | --- |
| ENSG00000072422 | RHOBTB1 | -1.70 | 0.31 | 7.46E-29 | down |
| ENSG00000004799 | PDK4 | -1.64 | 0.32 | 1.28E-30 | down |
| ENSG00000112378 | PERP | -1.63 | 0.32 | 3.63E-41 | down |
| ENSG00000147027 | TMEM47 | -1.58 | 0.34 | 3.41E-14 | down |
| ENSG00000147955 | SIGMAR1 | -1.54 | 0.34 | 1.61E-32 | down |
| ENSG00000166595 | FAM96B | -1.45 | 0.37 | 9.94E-13 | down |
| ENSG00000011465 | DCN | -1.43 | 0.37 | 1.11E-15 | down |
| ENSG00000074410 | CA12 | -1.35 | 0.39 | 3.7E-13 | down |
| ENSG00000157570 | TSPAN18 | -1.33 | 0.40 | 1.44E-10 | down |
| ENSG00000115884 | SDC1 | -1.32 | 0.40 | 2.4E-22 | down |
| ENSG00000107159 | CA9 | -1.29 | 0.41 | 8.3E-09 | down |
| ENSG00000166025 | AMOTL1 | -1.27 | 0.41 | 9.06E-16 | down |
| ENSG00000107295 | SH3GL2 | -1.24 | 0.42 | 4.66E-08 | down |
| ENSG00000114023 | FAM162A | -1.24 | 0.42 | 4.53E-15 | down |
| ENSG00000112149 | CD83 | -1.23 | 0.43 | 2.91E-17 | down |
| ENSG00000145545 | SRD5A1 | -1.21 | 0.43 | 2.39E-15 | down |
| ENSG00000163527 | STT3B | -1.21 | 0.43 | 3.11E-22 | down |
| ENSG00000006611 | USH1C | -1.20 | 0.43 | 1.03E-19 | down |
| ENSG00000111885 | MAN1A1 | -1.19 | 0.44 | 1.72E-08 | down |
| ENSG00000117984 | CTSD | -1.18 | 0.44 | 7.21E-09 | down |
| ENSG00000165476 | REEP3 | -1.18 | 0.44 | 3.57E-13 | down |
| ENSG00000122884 | P4HA1 | -1.18 | 0.44 | 3.16E-19 | down |
| ENSG00000175183 | CSRP2 | -1.17 | 0.44 | 4.51E-08 | down |
| ENSG00000149380 | P4HA3 | -1.15 | 0.45 | 2.62E-09 | down |
| ENSG00000125730 | C3 | -1.14 | 0.45 | 6.22E-21 | down |
| ENSG00000177189 | RPS6KA3 | -1.10 | 0.47 | 1.25E-17 | down |
| ENSG00000188060 | RAB42 | -1.10 | 0.47 | 3.01E-08 | down |
| ENSG00000181418 | DDN | -1.08 | 0.47 | 4.2E-07 | down |
| ENSG00000100292 | HMOX1 | -1.07 | 0.48 | 2.66E-06 | down |
| ENSG00000204174 | NPY4R | -1.06 | 0.48 | 3.15E-06 | down |
| ENSG00000078114 | NEBL | -1.06 | 0.48 | 1.07E-06 | down |
| ENSG00000011201 | KAL1 | -1.03 | 0.49 | 3.96E-07 | down |
| ENSG00000151632 | AKR1C2 | -1.03 | 0.49 | 6.31E-14 | down |
| ENSG00000022267 | FHL1 | -1.03 | 0.49 | 1.48E-06 | down |
| ENSG00000101654 | RNMT | -1.03 | 0.49 | 8.78E-09 | down |
| ENSG00000073756 | PTGS2 | -1.03 | 0.49 | 8.59E-08 | down |
| ENSG00000162006 | MSLNL | -1.02 | 0.49 | 0.0000078 | down |
| ENSG00000069275 | NUCKS1 | -1.00 | 0.50 | 1.31E-23 | down |
| ENSG00000145623 | OSMR | -1.00 | 0.50 | 1.15E-13 | down |
| ENSG00000158985 | CDC42SE2 | -0.99 | 0.50 | 1.43E-08 | down |
| ENSG00000166250 | CLMP | -0.99 | 0.50 | 8.11E-10 | down |
| ENSG00000109819 | PPARGC1A | -0.99 | 0.50 | 3.22E-06 | down |
| ENSG00000251095 | RP11-115D19.1 | -0.97 | 0.51 | 2.31E-06 | down |
| ENSG00000182158 | CREB3L2 | -0.97 | 0.51 | 9.31E-19 | down |
| ENSG00000126821 | SGPP1 | -0.96 | 0.51 | 3.93E-07 | down |
| ENSG00000151414 | NEK7 | -0.96 | 0.52 | 6.5E-08 | down |
| ENSG00000101076 | HNF4A | -0.96 | 0.52 | 0.0000293 | down |
| ENSG00000175040 | CHST2 | -0.95 | 0.52 | 1.5E-08 | down |
| ENSG00000170265 | ZNF282 | -0.95 | 0.52 | 2.78E-08 | down |
| ENSG00000163295 | ALPI | -0.94 | 0.52 | 1.32E-20 | down |
| ENSG00000082438 | COBLL1 | -0.93 | 0.52 | 8.16E-06 | down |
| ENSG00000164638 | SLC29A4 | -0.93 | 0.53 | 4.84E-06 | down |
| ENSG00000187134 | AKR1C1 | -0.92 | 0.53 | 0.0000432 | down |
| ENSG00000188761 | BCL2L15 | -0.92 | 0.53 | 0.0000548 | down |
| ENSG00000181467 | RAP2B | -0.92 | 0.53 | 8.65E-08 | down |
| ENSG00000143320 | CRABP2 | -0.92 | 0.53 | 7.55E-06 | down |
| ENSG00000165480 | SKA3 | -0.91 | 0.53 | 0.0000173 | down |
| ENSG00000177432 | NAP1L5 | -0.91 | 0.53 | 0.0000512 | down |
| ENSG00000186951 | PPARA | -0.90 | 0.54 | 0.00002 | down |
| ENSG00000121769 | FABP3 | -0.90 | 0.54 | 1.46E-06 | down |
| ENSG00000119138 | KLF9 | -0.88 | 0.54 | 0.0000293 | down |
| ENSG00000144802 | NFKBIZ | -0.88 | 0.54 | 3.34E-06 | down |
| ENSG00000143365 | RORC | -0.87 | 0.55 | 0.000132 | down |
| ENSG00000171631 | P2RY6 | -0.87 | 0.55 | 0.0000194 | down |
| ENSG00000070214 | SLC44A1 | -0.86 | 0.55 | 3.01E-07 | down |
| ENSG00000213064 | SFT2D2 | -0.85 | 0.55 | 3.16E-11 | down |
| ENSG00000137073 | UBAP2 | -0.84 | 0.56 | 0.0000102 | down |
| ENSG00000145365 | TIFA | -0.84 | 0.56 | 0.000229 | down |
| ENSG00000109107 | ALDOC | -0.84 | 0.56 | 8.61E-07 | down |
| ENSG00000135750 | KCNK1 | -0.84 | 0.56 | 2.12E-07 | down |
| ENSG00000164379 | FOXQ1 | -0.84 | 0.56 | 1.27E-09 | down |
| ENSG00000165895 | ARHGAP42 | -0.83 | 0.56 | 0.0000063 | down |
| ENSG00000117143 | UAP1 | -0.83 | 0.56 | 2.12E-09 | down |
| ENSG00000126709 | IFI6 | -0.83 | 0.56 | 0.0002507 | down |
| ENSG00000090266 | NDUFB2 | -0.83 | 0.56 | 0.0000112 | down |
| ENSG00000123836 | PFKFB2 | -0.82 | 0.56 | 3.38E-10 | down |
| ENSG00000143612 | C1orf43 | -0.82 | 0.57 | 2.33E-13 | down |
| ENSG00000056972 | TRAF3IP2 | -0.82 | 0.57 | 0.0002445 | down |
| ENSG00000196730 | DAPK1 | -0.82 | 0.57 | 4.81E-06 | down |
| ENSG00000102572 | STK24 | -0.82 | 0.57 | 2.02E-08 | down |
| ENSG00000198517 | MAFK | -0.82 | 0.57 | 5.19E-07 | down |
| ENSG00000198380 | GFPT1 | -0.82 | 0.57 | 1.69E-09 | down |
| ENSG00000240849 | TMEM189 | -0.82 | 0.57 | 0.0000651 | down |
| ENSG00000107249 | GLIS3 | -0.82 | 0.57 | 0.00012 | down |
| ENSG00000138413 | IDH1 | -0.82 | 0.57 | 1.61E-08 | down |
| ENSG00000151229 | SLC2A13 | -0.81 | 0.57 | 8.15E-06 | down |
| ENSG00000131089 | ARHGEF9 | -0.81 | 0.57 | 0.0002347 | down |
| ENSG00000148180 | GSN | -0.80 | 0.57 | 1.15E-07 | down |
| ENSG00000204713 | TRIM27 | -0.80 | 0.58 | 2.53E-07 | down |
| ENSG00000013619 | MAMLD1 | -0.80 | 0.58 | 0.000066 | down |
| ENSG00000159658 | EFCAB14 | -0.79 | 0.58 | 5.35E-07 | down |
| ENSG00000085788 | DDHD2 | -0.78 | 0.58 | 2.22E-06 | down |
| ENSG00000196743 | GM2A | -0.78 | 0.58 | 0.0001212 | down |
| ENSG00000135535 | CD164 | -0.78 | 0.58 | 1.38E-08 | down |
| ENSG00000165983 | PTER | -0.77 | 0.59 | 0.0003932 | down |
| ENSG00000173218 | VANGL1 | -0.77 | 0.59 | 0.0000104 | down |
| ENSG00000008517 | IL32 | -0.77 | 0.59 | 1.05E-07 | down |
| ENSG00000159399 | HK2 | -0.77 | 0.59 | 2.08E-09 | down |
| ENSG00000165272 | AQP3 | -0.76 | 0.59 | 2.38E-09 | down |
| ENSG00000076706 | MCAM | -0.76 | 0.59 | 3.93E-14 | down |
| ENSG00000106771 | TMEM245 | -0.75 | 0.59 | 2.03E-09 | down |
| ENSG00000118496 | FBXO30 | -0.75 | 0.59 | 4.79E-06 | down |
| ENSG00000112697 | TMEM30A | -0.75 | 0.60 | 1.98E-06 | down |
| ENSG00000214078 | CPNE1 | -0.75 | 0.60 | 6.73E-08 | down |
| ENSG00000163286 | ALPPL2 | -0.74 | 0.60 | 0.0004097 | down |
| ENSG00000129422 | MTUS1 | -0.74 | 0.60 | 0.0004043 | down |
| ENSG00000112773 | FAM46A | -0.73 | 0.60 | 0.0000108 | down |
| ENSG00000162695 | SLC30A7 | -0.73 | 0.60 | 0.0001016 | down |
| ENSG00000165655 | ZNF503 | -0.73 | 0.60 | 9.27E-07 | down |
| ENSG00000053254 | FOXN3 | -0.73 | 0.60 | 0.0004096 | down |
| ENSG00000152256 | PDK1 | -0.73 | 0.60 | 0.0000866 | down |
| ENSG00000250337 | LINC01021 | -0.73 | 0.60 | 0.0000505 | down |
| ENSG00000111540 | RAB5B | -0.73 | 0.60 | 0.0010795 | down |
| ENSG00000019549 | SNAI2 | -0.72 | 0.61 | 0.0012399 | down |
| ENSG00000165434 | PGM2L1 | -0.72 | 0.61 | 0.0000355 | down |
| ENSG00000168404 | MLKL | -0.72 | 0.61 | 0.0001801 | down |
| ENSG00000166676 | TVP23A | -0.72 | 0.61 | 0.0000292 | down |
| ENSG00000125845 | BMP2 | -0.72 | 0.61 | 1.17E-07 | down |
| ENSG00000158104 | HPD | -0.72 | 0.61 | 0.0014419 | down |
| ENSG00000148429 | USP6NL | -0.71 | 0.61 | 0.0000429 | down |
| ENSG00000185088 | RPS27L | -0.71 | 0.61 | 2.06E-07 | down |
| ENSG00000163378 | EOGT | -0.71 | 0.61 | 0.0017807 | down |
| ENSG00000188994 | ZNF292 | -0.71 | 0.61 | 0.0000556 | down |
| ENSG00000114268 | PFKFB4 | -0.71 | 0.61 | 0.0002954 | down |
| ENSG00000169242 | EFNA1 | -0.70 | 0.61 | 3.2E-09 | down |
| ENSG00000176171 | BNIP3 | -0.70 | 0.62 | 7.48E-07 | down |
| ENSG00000093000 | NUP50 | -0.70 | 0.62 | 0.0000118 | down |
| ENSG00000172795 | DCP2 | -0.69 | 0.62 | 0.0004227 | down |
| ENSG00000163993 | S100P | -0.69 | 0.62 | 0.0000465 | down |
| ENSG00000171603 | CLSTN1 | -0.69 | 0.62 | 3.84E-11 | down |
| ENSG00000095303 | PTGS1 | -0.69 | 0.62 | 2.98E-06 | down |
| ENSG00000118495 | PLAGL1 | -0.69 | 0.62 | 0.000017 | down |
| ENSG00000165609 | NUDT5 | -0.67 | 0.63 | 0.0000556 | down |
| ENSG00000115365 | LANCL1 | -0.67 | 0.63 | 3.69E-06 | down |
| ENSG00000130844 | ZNF331 | -0.67 | 0.63 | 0.0000414 | down |
| ENSG00000162772 | ATF3 | -0.67 | 0.63 | 0.0010712 | down |
| ENSG00000196139 | AKR1C3 | -0.67 | 0.63 | 3.16E-08 | down |
| ENSG00000105698 | USF2 | -0.67 | 0.63 | 2.03E-06 | down |
| ENSG00000135678 | CPM | -0.67 | 0.63 | 6.62E-07 | down |
| ENSG00000112245 | PTP4A1 | -0.66 | 0.63 | 2.15E-11 | down |
| ENSG00000260231 | JHDM1D-AS1 | -0.66 | 0.63 | 0.0018649 | down |
| ENSG00000138434 | SSFA2 | -0.66 | 0.63 | 2.28E-09 | down |
| ENSG00000113739 | STC2 | -0.66 | 0.63 | 0.0001566 | down |
| ENSG00000006459 | KDM7A | -0.66 | 0.63 | 0.0001749 | down |
| ENSG00000165792 | METTL17 | -0.66 | 0.63 | 0.0032002 | down |
| ENSG00000103269 | RHBDL1 | -0.66 | 0.63 | 0.0014982 | down |
| ENSG00000114480 | GBE1 | -0.66 | 0.63 | 0.0001114 | down |
| ENSG00000235123 | DSCAM-AS1 | -0.66 | 0.63 | 0.0010418 | down |
| ENSG00000069849 | ATP1B3 | -0.65 | 0.64 | 2.2E-13 | down |
| ENSG00000204291 | COL15A1 | -0.65 | 0.64 | 1.41E-07 | down |
| ENSG00000204217 | BMPR2 | -0.65 | 0.64 | 0.0007517 | down |
| ENSG00000168646 | AXIN2 | -0.65 | 0.64 | 0.0027614 | down |
| ENSG00000100196 | KDELR3 | -0.64 | 0.64 | 0.0033646 | down |
| ENSG00000112763 | BTN2A1 | -0.64 | 0.64 | 0.0020928 | down |
| ENSG00000121057 | AKAP1 | -0.64 | 0.64 | 0.0003906 | down |
| ENSG00000158711 | ELK4 | -0.64 | 0.64 | 0.0000311 | down |
| ENSG00000165806 | CASP7 | -0.64 | 0.64 | 0.0022807 | down |
| ENSG00000103018 | CYB5B | -0.64 | 0.64 | 1.41E-10 | down |
| ENSG00000139842 | CUL4A | -0.63 | 0.64 | 8.57E-10 | down |
| ENSG00000178172 | SPINK6 | -0.63 | 0.64 | 0.0004059 | down |
| ENSG00000051382 | PIK3CB | -0.63 | 0.64 | 0.0002728 | down |
| ENSG00000134755 | DSC2 | -0.63 | 0.64 | 0.001885 | down |
| ENSG00000157778 | PSMG3 | -0.63 | 0.65 | 0.000135 | down |
| ENSG00000100906 | NFKBIA | -0.62 | 0.65 | 0.0019859 | down |
| ENSG00000071243 | ING3 | -0.62 | 0.65 | 0.0031684 | down |
| ENSG00000139874 | SSTR1 | -0.62 | 0.65 | 0.0022308 | down |
| ENSG00000226958 | CTD-2328D6.1 | -0.62 | 0.65 | 0.0007492 | down |
| ENSG00000198758 | EPS8L3 | -0.62 | 0.65 | 0.0030966 | down |
| ENSG00000149418 | ST14 | -0.62 | 0.65 | 0.0008032 | down |
| ENSG00000013364 | MVP | -0.62 | 0.65 | 0.0000263 | down |
| ENSG00000110195 | FOLR1 | -0.62 | 0.65 | 1.59E-11 | down |
| ENSG00000128422 | KRT17 | -0.61 | 0.65 | 0.0000185 | down |
| ENSG00000001084 | GCLC | -0.61 | 0.65 | 0.00015 | down |
| ENSG00000243335 | KCTD7 | -0.61 | 0.66 | 0.0024159 | down |
| ENSG00000115468 | EFHD1 | -0.61 | 0.66 | 0.0025843 | down |
| ENSG00000169884 | WNT10B | -0.61 | 0.66 | 2.74E-09 | down |
| ENSG00000182378 | PLCXD1 | -0.61 | 0.66 | 0.0021201 | down |
| ENSG00000157216 | SSBP3 | -0.61 | 0.66 | 0.0000302 | down |
| ENSG00000106415 | GLCCI1 | -0.61 | 0.66 | 0.002525 | down |
| ENSG00000150961 | SEC24D | -0.60 | 0.66 | 0.0007689 | down |
| ENSG00000131473 | ACLY | -0.60 | 0.66 | 9.34E-10 | down |
| ENSG00000198626 | RYR2 | -0.59 | 0.66 | 0.0024634 | down |
| ENSG00000111275 | ALDH2 | -0.59 | 0.66 | 0.0031705 | down |
| ENSG00000057252 | SOAT1 | -0.59 | 0.66 | 1.13E-08 | down |
| ENSG00000006576 | PHTF2 | -0.59 | 0.66 | 0.0007718 | down |
| ENSG00000182326 | C1S | -0.59 | 0.66 | 0.0019816 | down |
| ENSG00000133935 | C14orf1 | -0.59 | 0.66 | 0.0003229 | down |
| ENSG00000102034 | ELF4 | -0.59 | 0.66 | 0.0007555 | down |
| ENSG00000143575 | HAX1 | -0.59 | 0.66 | 0.0000353 | down |
| ENSG00000168743 | NPNT | -0.59 | 0.67 | 2.06E-07 | down |
| ENSG00000196352 | CD55 | -0.59 | 0.67 | 1.44E-11 | down |
| ENSG00000164024 | METAP1 | -0.59 | 0.67 | 0.0004628 | down |
| ENSG00000113648 | H2AFY | -0.59 | 0.67 | 7.12E-07 | down |
| ENSG00000152952 | PLOD2 | -0.58 | 0.67 | 0.00006 | down |
| ENSG00000155463 | OXA1L | -0.58 | 0.67 | 0.0000785 | down |
| ENSG00000097007 | ABL1 | -0.58 | 0.67 | 1.17E-06 | down |
| ENSG00000188483 | IER5L | -0.58 | 0.67 | 0.0008776 | down |
| ENSG00000175224 | ATG13 | -0.58 | 0.67 | 0.0001628 | down |
| ENSG00000134748 | PRPF38A | -0.58 | 0.67 | 0.0000638 | down |
| ENSG00000162769 | FLVCR1 | -0.58 | 0.67 | 0.0002874 | down |
| ENSG00000052802 | MSMO1 | -0.58 | 0.67 | 0.0000663 | down |
| ENSG00000130600 | H19 | -0.58 | 0.67 | 2.58E-12 | down |
| ENSG00000174013 | FBXO45 | -0.58 | 0.67 | 0.0028807 | down |
| ENSG00000077232 | DNAJC10 | -0.57 | 0.67 | 4.36E-06 | down |
| ENSG00000164211 | STARD4 | -0.57 | 0.67 | 0.0008518 | down |
| ENSG00000118454 | ANKRD13C | -0.57 | 0.67 | 0.001837 | down |
| ENSG00000082153 | BZW1 | -0.57 | 0.67 | 3.15E-08 | down |
| ENSG00000094916 | CBX5 | -0.57 | 0.67 | 1.19E-08 | down |
| ENSG00000142168 | SOD1 | -0.57 | 0.67 | 2.01E-07 | down |
| ENSG00000135269 | TES | -0.57 | 0.68 | 0.0000435 | down |
| ENSG00000196154 | S100A4 | -0.56 | 0.68 | 6.09E-08 | down |
| ENSG00000166681 | NGFRAP1 | -0.56 | 0.68 | 2.19E-06 | down |
| ENSG00000175265 | GOLGA8A | -0.56 | 0.68 | 0.0001902 | down |
| ENSG00000021826 | CPS1 | -0.56 | 0.68 | 1.42E-11 | down |
| ENSG00000119616 | FCF1 | -0.55 | 0.68 | 0.0004019 | down |
| ENSG00000113273 | ARSB | -0.55 | 0.68 | 0.002101 | down |
| ENSG00000165389 | SPTSSA | -0.55 | 0.68 | 0.0005473 | down |
| ENSG00000196517 | SLC6A9 | -0.54 | 0.69 | 0.0013315 | down |
| ENSG00000069535 | MAOB | -0.54 | 0.69 | 0.0000207 | down |
| ENSG00000117139 | KDM5B | -0.54 | 0.69 | 0.000029 | down |
| ENSG00000023839 | ABCC2 | -0.54 | 0.69 | 0.0001844 | down |
| ENSG00000184992 | BRI3BP | -0.54 | 0.69 | 0.0001389 | down |
| ENSG00000101846 | STS | -0.54 | 0.69 | 0.0011147 | down |
| ENSG00000177425 | PAWR | -0.53 | 0.69 | 0.0001778 | down |
| ENSG00000163435 | ELF3 | -0.53 | 0.69 | 0.0015622 | down |
| ENSG00000141232 | TOB1 | -0.53 | 0.69 | 0.0000193 | down |
| ENSG00000170955 | PRKCDBP | -0.53 | 0.69 | 0.0004206 | down |
| ENSG00000100335 | MIEF1 | -0.53 | 0.69 | 0.0015603 | down |
| ENSG00000164096 | C4orf3 | -0.53 | 0.69 | 0.001704 | down |
| ENSG00000159873 | CCDC117 | -0.53 | 0.69 | 0.0009943 | down |
| ENSG00000168291 | PDHB | -0.53 | 0.69 | 0.0020496 | down |
| ENSG00000143416 | SELENBP1 | -0.52 | 0.70 | 0.0012742 | down |
| ENSG00000062716 | VMP1 | -0.52 | 0.70 | 5.25E-06 | down |
| ENSG00000162804 | SNED1 | -0.52 | 0.70 | 0.0012578 | down |
| ENSG00000114302 | PRKAR2A | -0.52 | 0.70 | 0.0002338 | down |
| ENSG00000072682 | P4HA2 | -0.52 | 0.70 | 0.001213 | down |
| ENSG00000061794 | MRPS35 | -0.52 | 0.70 | 0.0023478 | down |
| ENSG00000175029 | CTBP2 | -0.52 | 0.70 | 0.0003589 | down |
| ENSG00000173546 | CSPG4 | -0.52 | 0.70 | 0.0001072 | down |
| ENSG00000168209 | DDIT4 | -0.52 | 0.70 | 0.0000689 | down |
| ENSG00000152102 | FAM168B | -0.52 | 0.70 | 0.000136 | down |
| ENSG00000187634 | SAMD11 | -0.52 | 0.70 | 0.0011564 | down |
| ENSG00000172380 | GNG12 | -0.51 | 0.70 | 0.0000234 | down |
| ENSG00000172572 | PDE3A | -0.51 | 0.70 | 0.0000326 | down |
| ENSG00000060749 | QSER1 | -0.51 | 0.70 | 0.0000634 | down |
| ENSG00000161642 | ZNF385A | -0.51 | 0.70 | 0.0028225 | down |
| ENSG00000116711 | PLA2G4A | -0.51 | 0.70 | 0.0012573 | down |
| ENSG00000213931 | HBE1 | -0.50 | 0.70 | 0.002462 | down |
| ENSG00000150995 | ITPR1 | -0.50 | 0.71 | 0.0000174 | down |
| ENSG00000159346 | ADIPOR1 | -0.50 | 0.71 | 0.0003534 | down |
| ENSG00000189057 | FAM111B | -0.49 | 0.71 | 0.0028756 | down |
| ENSG00000186480 | INSIG1 | -0.49 | 0.71 | 9.14E-06 | down |
| ENSG00000155254 | MARVELD1 | -0.49 | 0.71 | 0.0028238 | down |
| ENSG00000205765 | C5orf51 | -0.48 | 0.71 | 0.0008575 | down |
| ENSG00000182240 | BACE2 | -0.48 | 0.72 | 2.74E-07 | down |
| ENSG00000178695 | KCTD12 | -0.48 | 0.72 | 0.0011441 | down |
| ENSG00000132432 | SEC61G | -0.48 | 0.72 | 0.0001573 | down |
| ENSG00000104549 | SQLE | -0.48 | 0.72 | 0.0011148 | down |
| ENSG00000004700 | RECQL | -0.48 | 0.72 | 0.0029295 | down |
| ENSG00000132155 | RAF1 | -0.48 | 0.72 | 0.0002703 | down |
| ENSG00000162368 | CMPK1 | -0.48 | 0.72 | 0.0003004 | down |
| ENSG00000148730 | EIF4EBP2 | -0.47 | 0.72 | 0.0006923 | down |
| ENSG00000266714 | MYO15B | -0.47 | 0.72 | 0.0020943 | down |
| ENSG00000131174 | COX7B | -0.47 | 0.72 | 0.0011147 | down |
| ENSG00000125107 | CNOT1 | -0.47 | 0.72 | 0.0000104 | down |
| ENSG00000255717 | SNHG1 | -0.47 | 0.72 | 0.0011926 | down |
| ENSG00000135486 | HNRNPA1 | -0.46 | 0.73 | 1.51E-07 | down |
| ENSG00000038219 | BOD1L1 | -0.46 | 0.73 | 0.0013074 | down |
| ENSG00000136997 | MYC | -0.46 | 0.73 | 0.000113 | down |
| ENSG00000013374 | NUB1 | -0.46 | 0.73 | 0.001186 | down |
| ENSG00000121690 | DEPDC7 | -0.45 | 0.73 | 0.0004185 | down |
| ENSG00000185499 | MUC1 | -0.45 | 0.73 | 0.0025694 | down |
| ENSG00000134107 | BHLHE40 | -0.45 | 0.73 | 0.0004214 | down |
| ENSG00000117394 | SLC2A1 | -0.44 | 0.74 | 0.0000246 | down |
| ENSG00000090013 | BLVRB | -0.44 | 0.74 | 0.0025303 | down |
| ENSG00000134287 | ARF3 | -0.44 | 0.74 | 0.0000229 | down |
| ENSG00000104388 | RAB2A | -0.44 | 0.74 | 0.0008775 | down |
| ENSG00000164466 | SFXN1 | -0.44 | 0.74 | 0.0002924 | down |
| ENSG00000143198 | MGST3 | -0.43 | 0.74 | 0.0021339 | down |
| ENSG00000137504 | CREBZF | -0.43 | 0.74 | 0.0002567 | down |
| ENSG00000185551 | NR2F2 | -0.43 | 0.74 | 0.0023927 | down |
| ENSG00000144381 | HSPD1 | -0.43 | 0.74 | 6.31E-06 | down |
| ENSG00000196683 | TOMM7 | -0.43 | 0.74 | 0.0008486 | down |
| ENSG00000162613 | FUBP1 | -0.43 | 0.74 | 0.000239 | down |
| ENSG00000146247 | PHIP | -0.43 | 0.74 | 0.0026388 | down |
| ENSG00000112667 | DNPH1 | -0.43 | 0.74 | 0.002804 | down |
| ENSG00000079332 | SAR1A | -0.43 | 0.74 | 0.0005685 | down |
| ENSG00000176915 | ANKLE2 | -0.43 | 0.74 | 0.0001454 | down |
| ENSG00000163479 | SSR2 | -0.42 | 0.75 | 0.0002777 | down |
| ENSG00000075426 | FOSL2 | -0.42 | 0.75 | 0.0001936 | down |
| ENSG00000168374 | ARF4 | -0.42 | 0.75 | 0.0015212 | down |
| ENSG00000008513 | ST3GAL1 | -0.42 | 0.75 | 0.003381 | down |
| ENSG00000171992 | SYNPO | -0.42 | 0.75 | 0.0008316 | down |
| ENSG00000177954 | RPS27 | -0.42 | 0.75 | 0.0000196 | down |
| ENSG00000135940 | COX5B | -0.42 | 0.75 | 0.0004454 | down |
| ENSG00000148175 | STOM | -0.42 | 0.75 | 0.0012669 | down |
| ENSG00000134884 | ARGLU1 | -0.41 | 0.75 | 0.0006811 | down |
| ENSG00000070087 | PFN2 | -0.41 | 0.75 | 0.0009258 | down |
| ENSG00000126432 | PRDX5 | -0.41 | 0.75 | 0.0001177 | down |
| ENSG00000196262 | PPIA | -0.41 | 0.75 | 1.18E-06 | down |
| ENSG00000185585 | OLFML2A | -0.41 | 0.76 | 0.0007611 | down |
| ENSG00000104765 | BNIP3L | -0.40 | 0.76 | 0.000666 | down |
| ENSG00000204054 | LINC00963 | -0.40 | 0.76 | 0.0003145 | down |
| ENSG00000185215 | TNFAIP2 | -0.40 | 0.76 | 0.000493 | down |
| ENSG00000155368 | DBI | -0.40 | 0.76 | 0.0012359 | down |
| ENSG00000189067 | LITAF | -0.40 | 0.76 | 0.0008691 | down |
| ENSG00000042493 | CAPG | -0.40 | 0.76 | 0.0012567 | down |
| ENSG00000174915 | PTDSS2 | -0.40 | 0.76 | 0.0000945 | down |
| ENSG00000159202 | UBE2Z | -0.40 | 0.76 | 0.0019148 | down |
| ENSG00000092621 | PHGDH | -0.39 | 0.76 | 0.0009015 | down |
| ENSG00000184990 | SIVA1 | -0.39 | 0.76 | 0.0032741 | down |
| ENSG00000163430 | FSTL1 | -0.39 | 0.76 | 0.0003892 | down |
| ENSG00000141905 | NFIC | -0.39 | 0.76 | 0.0011543 | down |
| ENSG00000184743 | ATL3 | -0.39 | 0.76 | 0.0024094 | down |
| ENSG00000130707 | ASS1 | -0.39 | 0.76 | 1.32E-06 | down |
| ENSG00000138668 | HNRNPD | -0.38 | 0.77 | 0.0001284 | down |
| ENSG00000173575 | CHD2 | -0.38 | 0.77 | 0.0027518 | down |
| ENSG00000183283 | DAZAP2 | -0.38 | 0.77 | 0.0014837 | down |
| ENSG00000184007 | PTP4A2 | -0.38 | 0.77 | 0.0005038 | down |
| ENSG00000075785 | RAB7A | -0.37 | 0.77 | 0.0005423 | down |
| ENSG00000105373 | GLTSCR2 | -0.37 | 0.77 | 0.0021268 | down |
| ENSG00000152234 | ATP5A1 | -0.37 | 0.77 | 0.0004048 | down |
| ENSG00000091009 | RBM27 | -0.37 | 0.78 | 0.0014271 | down |
| ENSG00000075239 | ACAT1 | -0.36 | 0.78 | 0.0014889 | down |
| ENSG00000077942 | FBLN1 | -0.36 | 0.78 | 0.0003907 | down |
| ENSG00000145907 | G3BP1 | -0.36 | 0.78 | 0.0011206 | down |
| ENSG00000107262 | BAG1 | -0.36 | 0.78 | 0.0019047 | down |
| ENSG00000161638 | ITGA5 | -0.36 | 0.78 | 0.0022917 | down |
| ENSG00000102144 | PGK1 | -0.36 | 0.78 | 0.0000212 | down |
| ENSG00000125691 | RPL23 | -0.36 | 0.78 | 0.0002793 | down |
| ENSG00000170017 | ALCAM | -0.36 | 0.78 | 0.002989 | down |
| ENSG00000063046 | EIF4B | -0.36 | 0.78 | 0.0000348 | down |
| ENSG00000109475 | RPL34 | -0.36 | 0.78 | 0.0013596 | down |
| ENSG00000176986 | SEC24C | -0.35 | 0.78 | 0.0013579 | down |
| ENSG00000163468 | CCT3 | -0.35 | 0.78 | 0.0001979 | down |
| ENSG00000245532 | NEAT1 | -0.35 | 0.78 | 0.0000554 | down |
| ENSG00000147162 | OGT | -0.35 | 0.79 | 0.0013585 | down |
| ENSG00000197956 | S100A6 | -0.35 | 0.79 | 0.0021786 | down |
| ENSG00000110700 | RPS13 | -0.34 | 0.79 | 0.00061 | down |
| ENSG00000175061 | FAM211A-AS1 | -0.34 | 0.79 | 0.0006347 | down |
| ENSG00000051523 | CYBA | -0.34 | 0.79 | 0.0031107 | down |
| ENSG00000123843 | C4BPB | -0.34 | 0.79 | 0.0019445 | down |
| ENSG00000180537 | RNF182 | -0.34 | 0.79 | 0.0030255 | down |
| ENSG00000148248 | SURF4 | -0.33 | 0.80 | 0.0007866 | down |
| ENSG00000124783 | SSR1 | -0.32 | 0.80 | 0.0034127 | down |
| ENSG00000086062 | B4GALT1 | -0.32 | 0.80 | 0.0026479 | down |
| ENSG00000149925 | ALDOA | -0.32 | 0.80 | 0.0007498 | down |
| ENSG00000143878 | RHOB | -0.32 | 0.80 | 0.0030136 | down |
| ENSG00000229117 | RPL41 | -0.31 | 0.80 | 0.000655 | down |
| ENSG00000105220 | GPI | -0.31 | 0.80 | 0.0010193 | down |
| ENSG00000083845 | RPS5 | -0.31 | 0.81 | 0.0010594 | down |
| ENSG00000171858 | RPS21 | -0.31 | 0.81 | 0.0018105 | down |
| ENSG00000171314 | PGAM1 | -0.31 | 0.81 | 0.001027 | down |
| ENSG00000111669 | TPI1 | -0.31 | 0.81 | 0.0004087 | down |
| ENSG00000172270 | BSG | -0.30 | 0.81 | 0.0017128 | down |
| ENSG00000166710 | B2M | -0.30 | 0.81 | 0.0009433 | down |
| ENSG00000137699 | TRIM29 | -0.29 | 0.82 | 0.0033561 | down |
| ENSG00000142178 | SIK1 | -0.29 | 0.82 | 0.0022453 | down |
| ENSG00000139644 | TMBIM6 | -0.28 | 0.82 | 0.0021322 | down |
| ENSG00000105193 | RPS16 | -0.28 | 0.82 | 0.0011661 | down |
| ENSG00000143153 | ATP1B1 | -0.28 | 0.83 | 0.001944 | down |
| ENSG00000067225 | PKM | -0.28 | 0.83 | 0.0007462 | down |
| ENSG00000134333 | LDHA | -0.27 | 0.83 | 0.0009445 | down |
| ENSG00000174748 | RPL15 | -0.27 | 0.83 | 0.0021977 | down |
| ENSG00000156508 | EEF1A1 | -0.26 | 0.83 | 0.0008266 | down |
| ENSG00000142541 | RPL13A | -0.26 | 0.83 | 0.0023115 | down |
| ENSG00000173391 | OLR1 | 1.52 | 2.87 | 2.93E-20 | up |
| ENSG00000135318 | NT5E | 1.49 | 2.80 | 4.847E-11 | up |
| ENSG00000167601 | AXL | 1.31 | 2.48 | 9.798E-22 | up |
| ENSG00000112902 | SEMA5A | 1.29 | 2.45 | 7.359E-12 | up |
| ENSG00000204899 | MZT1 | 1.26 | 2.40 | 3.103E-12 | up |
| ENSG00000115380 | EFEMP1 | 1.22 | 2.33 | 5.338E-23 | up |
| ENSG00000196526 | AFAP1 | 1.11 | 2.16 | 1.958E-08 | up |
| ENSG00000134531 | EMP1 | 1.11 | 2.16 | 4.588E-10 | up |
| ENSG00000175832 | ETV4 | 1.08 | 2.12 | 5.703E-08 | up |
| ENSG00000133816 | MICAL2 | 1.04 | 2.05 | 5.234E-07 | up |
| ENSG00000146072 | TNFRSF21 | 1.00 | 1.99 | 1.053E-05 | up |
| ENSG00000180447 | GAS1 | 0.95 | 1.93 | 2.166E-05 | up |
| ENSG00000105810 | CDK6 | 0.94 | 1.92 | 1.966E-21 | up |
| ENSG00000139645 | ANKRD52 | 0.92 | 1.89 | 9.794E-15 | up |
| ENSG00000112078 | KCTD20 | 0.91 | 1.88 | 2.633E-12 | up |
| ENSG00000132470 | ITGB4 | 0.90 | 1.87 | 4.151E-10 | up |
| ENSG00000074527 | NTN4 | 0.90 | 1.87 | 1.143E-05 | up |
| ENSG00000196937 | FAM3C | 0.90 | 1.86 | 2.951E-06 | up |
| ENSG00000100697 | DICER1 | 0.90 | 1.86 | 8.698E-10 | up |
| ENSG00000144959 | NCEH1 | 0.89 | 1.86 | 7.391E-08 | up |
| ENSG00000142871 | CYR61 | 0.89 | 1.86 | 9.585E-12 | up |
| ENSG00000150782 | IL18 | 0.89 | 1.86 | 1.214E-08 | up |
| ENSG00000259758 | CASC7 | 0.89 | 1.85 | 3.689E-10 | up |
| ENSG00000125148 | MT2A | 0.88 | 1.84 | 1.075E-08 | up |
| ENSG00000179869 | ABCA13 | 0.87 | 1.83 | 7.338E-05 | up |
| ENSG00000049656 | CLPTM1L | 0.87 | 1.82 | 1.102E-23 | up |
| ENSG00000122870 | BICC1 | 0.84 | 1.80 | 5.621E-09 | up |
| ENSG00000135052 | GOLM1 | 0.84 | 1.79 | 1.632E-12 | up |
| ENSG00000134717 | BTF3L4 | 0.83 | 1.78 | 3.28E-07 | up |
| ENSG00000159263 | SIM2 | 0.83 | 1.77 | 1.025E-05 | up |
| ENSG00000175592 | FOSL1 | 0.82 | 1.77 | 0.0002306 | up |
| ENSG00000131018 | SYNE1 | 0.82 | 1.76 | 8.066E-08 | up |
| ENSG00000115414 | FN1 | 0.78 | 1.72 | 0.000491 | up |
| ENSG00000113594 | LIFR | 0.78 | 1.72 | 3.015E-11 | up |
| ENSG00000185697 | MYBL1 | 0.78 | 1.72 | 0.0001501 | up |
| ENSG00000172239 | PAIP1 | 0.77 | 1.71 | 7.294E-12 | up |
| ENSG00000150712 | MTMR12 | 0.77 | 1.70 | 1.394E-07 | up |
| ENSG00000115762 | PLEKHB2 | 0.76 | 1.70 | 6.675E-06 | up |
| ENSG00000111110 | PPM1H | 0.76 | 1.70 | 0.0003094 | up |
| ENSG00000166825 | ANPEP | 0.76 | 1.69 | 2.394E-05 | up |
| ENSG00000164134 | NAA15 | 0.75 | 1.69 | 1.125E-06 | up |
| ENSG00000154127 | UBASH3B | 0.75 | 1.68 | 2.001E-05 | up |
| ENSG00000198223 | CSF2RA | 0.74 | 1.67 | 0.0006826 | up |
| ENSG00000160179 | ABCG1 | 0.74 | 1.67 | 0.0003226 | up |
| ENSG00000076864 | RAP1GAP | 0.74 | 1.67 | 5.489E-05 | up |
| ENSG00000168077 | SCARA3 | 0.74 | 1.67 | 2.354E-10 | up |
| ENSG00000129925 | TMEM8A | 0.74 | 1.67 | 1.083E-05 | up |
| ENSG00000130202 | PVRL2 | 0.73 | 1.66 | 1.917E-05 | up |
| ENSG00000188610 | FAM72B | 0.71 | 1.64 | 0.000909 | up |
| ENSG00000130956 | HABP4 | 0.71 | 1.64 | 0.0017117 | up |
| ENSG00000183696 | UPP1 | 0.71 | 1.63 | 0.0006962 | up |
| ENSG00000166851 | PLK1 | 0.70 | 1.62 | 7.319E-08 | up |
| ENSG00000140564 | FURIN | 0.70 | 1.62 | 4.473E-09 | up |
| ENSG00000102931 | ARL2BP | 0.70 | 1.62 | 0.002096 | up |
| ENSG00000204388 | HSPA1B | 0.70 | 1.62 | 7.358E-08 | up |
| ENSG00000110092 | CCND1 | 0.68 | 1.60 | 8.912E-07 | up |
| ENSG00000112576 | CCND3 | 0.68 | 1.60 | 2.346E-09 | up |
| ENSG00000174977 | AC026271.5 | 0.67 | 1.60 | 0.0022435 | up |
| ENSG00000144118 | RALB | 0.67 | 1.59 | 9.28E-06 | up |
| ENSG00000106070 | GRB10 | 0.67 | 1.59 | 0.0004569 | up |
| ENSG00000123104 | ITPR2 | 0.67 | 1.59 | 0.000153 | up |
| ENSG00000080824 | HSP90AA1 | 0.67 | 1.59 | 1.542E-14 | up |
| ENSG00000115109 | EPB41L5 | 0.66 | 1.58 | 0.0010362 | up |
| ENSG00000143322 | ABL2 | 0.66 | 1.58 | 2.635E-06 | up |
| ENSG00000163513 | TGFBR2 | 0.66 | 1.58 | 3.327E-05 | up |
| ENSG00000123908 | AGO2 | 0.66 | 1.58 | 4.699E-05 | up |
| ENSG00000169604 | ANTXR1 | 0.65 | 1.57 | 5.424E-08 | up |
| ENSG00000119397 | CNTRL | 0.65 | 1.57 | 0.0006331 | up |
| ENSG00000197535 | MYO5A | 0.65 | 1.57 | 0.0002731 | up |
| ENSG00000089597 | GANAB | 0.65 | 1.57 | 4.56E-12 | up |
| ENSG00000170485 | NPAS2 | 0.65 | 1.57 | 0.0005489 | up |
| ENSG00000067141 | NEO1 | 0.64 | 1.56 | 0.0004193 | up |
| ENSG00000100605 | ITPK1 | 0.64 | 1.56 | 0.0003171 | up |
| ENSG00000203485 | INF2 | 0.64 | 1.56 | 4.884E-08 | up |
| ENSG00000137831 | UACA | 0.64 | 1.56 | 0.000211 | up |
| ENSG00000121753 | BAI2 | 0.63 | 1.55 | 0.0014109 | up |
| ENSG00000155850 | SLC26A2 | 0.63 | 1.55 | 0.000449 | up |
| ENSG00000152818 | UTRN | 0.63 | 1.55 | 0.0004569 | up |
| ENSG00000108262 | GIT1 | 0.63 | 1.55 | 0.0001496 | up |
| ENSG00000134057 | CCNB1 | 0.62 | 1.54 | 6.967E-08 | up |
| ENSG00000006327 | TNFRSF12A | 0.62 | 1.54 | 3.372E-06 | up |
| ENSG00000162745 | OLFML2B | 0.62 | 1.54 | 0.0031736 | up |
| ENSG00000094631 | HDAC6 | 0.62 | 1.54 | 0.0005034 | up |
| ENSG00000138138 | ATAD1 | 0.62 | 1.54 | 8.411E-05 | up |
| ENSG00000166483 | WEE1 | 0.61 | 1.53 | 0.0001732 | up |
| ENSG00000184216 | IRAK1 | 0.61 | 1.53 | 5.108E-09 | up |
| ENSG00000141449 | GREB1L | 0.61 | 1.53 | 0.000997 | up |
| ENSG00000135476 | ESPL1 | 0.60 | 1.52 | 7.29E-06 | up |
| ENSG00000170558 | CDH2 | 0.60 | 1.52 | 0.0021593 | up |
| ENSG00000123143 | PKN1 | 0.60 | 1.51 | 1.955E-05 | up |
| ENSG00000105971 | CAV2 | 0.60 | 1.51 | 3.114E-05 | up |
| ENSG00000120885 | CLU | 0.60 | 1.51 | 1.073E-10 | up |
| ENSG00000169439 | SDC2 | 0.59 | 1.51 | 0.0024469 | up |
| ENSG00000187079 | TEAD1 | 0.59 | 1.51 | 8.353E-10 | up |
| ENSG00000054793 | ATP9A | 0.59 | 1.50 | 1.42E-06 | up |
| ENSG00000008256 | CYTH3 | 0.58 | 1.50 | 2.828E-05 | up |
| ENSG00000064666 | CNN2 | 0.58 | 1.50 | 1.656E-05 | up |
| ENSG00000140945 | CDH13 | 0.58 | 1.50 | 0.0007891 | up |
| ENSG00000184792 | OSBP2 | 0.58 | 1.50 | 0.00013 | up |
| ENSG00000114019 | AMOTL2 | 0.58 | 1.50 | 3.604E-05 | up |
| ENSG00000198793 | MTOR | 0.58 | 1.49 | 7.901E-06 | up |
| ENSG00000198947 | DMD | 0.58 | 1.49 | 3.797E-06 | up |
| ENSG00000154864 | PIEZO2 | 0.58 | 1.49 | 0.002423 | up |
| ENSG00000124225 | PMEPA1 | 0.58 | 1.49 | 4.741E-05 | up |
| ENSG00000198682 | PAPSS2 | 0.57 | 1.49 | 0.0022753 | up |
| ENSG00000101057 | MYBL2 | 0.57 | 1.49 | 1.877E-07 | up |
| ENSG00000157191 | NECAP2 | 0.57 | 1.48 | 0.0023039 | up |
| ENSG00000145779 | TNFAIP8 | 0.57 | 1.48 | 0.0017373 | up |
| ENSG00000110917 | MLEC | 0.57 | 1.48 | 4.848E-05 | up |
| ENSG00000171793 | CTPS1 | 0.56 | 1.48 | 5.764E-06 | up |
| ENSG00000169715 | MT1E | 0.56 | 1.48 | 0.0024846 | up |
| ENSG00000116679 | IVNS1ABP | 0.56 | 1.48 | 4.823E-06 | up |
| ENSG00000072571 | HMMR | 0.56 | 1.48 | 0.0004654 | up |
| ENSG00000100345 | MYH9 | 0.56 | 1.47 | 3.442E-09 | up |
| ENSG00000110047 | EHD1 | 0.56 | 1.47 | 0.0003205 | up |
| ENSG00000049323 | LTBP1 | 0.56 | 1.47 | 3.893E-07 | up |
| ENSG00000132002 | DNAJB1 | 0.56 | 1.47 | 3.656E-06 | up |
| ENSG00000151012 | SLC7A11 | 0.56 | 1.47 | 0.0001551 | up |
| ENSG00000142945 | KIF2C | 0.55 | 1.47 | 4.275E-05 | up |
| ENSG00000077782 | FGFR1 | 0.55 | 1.47 | 0.0015013 | up |
| ENSG00000065060 | UHRF1BP1 | 0.55 | 1.47 | 0.0006398 | up |
| ENSG00000071054 | MAP4K4 | 0.55 | 1.47 | 0.0007699 | up |
| ENSG00000119139 | TJP2 | 0.55 | 1.46 | 0.0003542 | up |
| ENSG00000198752 | CDC42BPB | 0.55 | 1.46 | 1.141E-05 | up |
| ENSG00000142627 | EPHA2 | 0.55 | 1.46 | 4.848E-05 | up |
| ENSG00000110218 | PANX1 | 0.54 | 1.46 | 0.0028743 | up |
| ENSG00000097021 | ACOT7 | 0.54 | 1.46 | 0.0010045 | up |
| ENSG00000137807 | KIF23 | 0.54 | 1.45 | 3.531E-05 | up |
| ENSG00000171848 | RRM2 | 0.54 | 1.45 | 3.05E-06 | up |
| ENSG00000092445 | TYRO3 | 0.54 | 1.45 | 0.0002969 | up |
| ENSG00000167767 | KRT80 | 0.53 | 1.45 | 1.121E-05 | up |
| ENSG00000121621 | KIF18A | 0.53 | 1.45 | 0.0022813 | up |
| ENSG00000101224 | CDC25B | 0.53 | 1.45 | 5.446E-05 | up |
| ENSG00000138119 | MYOF | 0.53 | 1.44 | 1.195E-08 | up |
| ENSG00000100401 | RANGAP1 | 0.53 | 1.44 | 1.463E-05 | up |
| ENSG00000091527 | CDV3 | 0.53 | 1.44 | 2.333E-06 | up |
| ENSG00000040275 | SPDL1 | 0.53 | 1.44 | 2.558E-05 | up |
| ENSG00000072110 | ACTN1 | 0.52 | 1.44 | 1.692E-06 | up |
| ENSG00000146648 | EGFR | 0.52 | 1.44 | 2.063E-08 | up |
| ENSG00000109971 | HSPA8 | 0.52 | 1.44 | 8.51E-10 | up |
| ENSG00000164733 | CTSB | 0.52 | 1.43 | 0.0001861 | up |
| ENSG00000087586 | AURKA | 0.52 | 1.43 | 2.243E-05 | up |
| ENSG00000076003 | MCM6 | 0.51 | 1.43 | 0.0001558 | up |
| ENSG00000179837 | RBM15B | 0.51 | 1.43 | 0.0006861 | up |
| ENSG00000105974 | CAV1 | 0.51 | 1.43 | 0.0001751 | up |
| ENSG00000134243 | SORT1 | 0.51 | 1.42 | 0.0019659 | up |
| ENSG00000162734 | PEA15 | 0.51 | 1.42 | 0.0003817 | up |
| ENSG00000106086 | PLEKHA8 | 0.51 | 1.42 | 0.0028593 | up |
| ENSG00000088325 | TPX2 | 0.51 | 1.42 | 9.037E-07 | up |
| ENSG00000136717 | BIN1 | 0.51 | 1.42 | 2.288E-05 | up |
| ENSG00000128944 | KNSTRN | 0.50 | 1.42 | 0.0012741 | up |
| ENSG00000118193 | KIF14 | 0.50 | 1.42 | 0.0002977 | up |
| ENSG00000156273 | BACH1 | 0.50 | 1.42 | 0.000677 | up |
| ENSG00000074696 | PTPLAD1 | 0.50 | 1.42 | 0.0002534 | up |
| ENSG00000108106 | UBE2S | 0.50 | 1.42 | 0.0001596 | up |
| ENSG00000198113 | TOR4A | 0.50 | 1.41 | 0.0002266 | up |
| ENSG00000145555 | MYO10 | 0.50 | 1.41 | 1.147E-08 | up |
| ENSG00000116016 | EPAS1 | 0.49 | 1.41 | 0.0001155 | up |
| ENSG00000096384 | HSP90AB1 | 0.49 | 1.41 | 1.486E-09 | up |
| ENSG00000122863 | CHST3 | 0.49 | 1.40 | 0.0001025 | up |
| ENSG00000152767 | FARP1 | 0.49 | 1.40 | 4.022E-05 | up |
| ENSG00000136205 | TNS3 | 0.49 | 1.40 | 0.0001713 | up |
| ENSG00000071539 | TRIP13 | 0.49 | 1.40 | 1.825E-05 | up |
| ENSG00000003989 | SLC7A2 | 0.49 | 1.40 | 0.0003854 | up |
| ENSG00000117399 | CDC20 | 0.48 | 1.40 | 0.0001376 | up |
| ENSG00000101150 | TPD52L2 | 0.48 | 1.40 | 2.755E-05 | up |
| ENSG00000104881 | PPP1R13L | 0.48 | 1.39 | 0.0007249 | up |
| ENSG00000198142 | SOWAHC | 0.48 | 1.39 | 0.0003239 | up |
| ENSG00000084774 | CAD | 0.47 | 1.39 | 5.792E-05 | up |
| ENSG00000146376 | ARHGAP18 | 0.47 | 1.39 | 0.0005062 | up |
| ENSG00000114999 | TTL | 0.47 | 1.39 | 0.0001802 | up |
| ENSG00000171148 | TADA3 | 0.47 | 1.39 | 0.000689 | up |
| ENSG00000123975 | CKS2 | 0.47 | 1.39 | 0.0002309 | up |
| ENSG00000144283 | PKP4 | 0.47 | 1.39 | 0.00305 | up |
| ENSG00000104889 | RNASEH2A | 0.47 | 1.38 | 0.0009998 | up |
| ENSG00000138772 | ANXA3 | 0.47 | 1.38 | 0.0032106 | up |
| ENSG00000075624 | ACTB | 0.47 | 1.38 | 3.303E-09 | up |
| ENSG00000120948 | TARDBP | 0.47 | 1.38 | 7.351E-05 | up |
| ENSG00000110756 | HPS5 | 0.47 | 1.38 | 0.0015604 | up |
| ENSG00000160014 | CALM3 | 0.47 | 1.38 | 0.0001933 | up |
| ENSG00000157456 | CCNB2 | 0.46 | 1.38 | 0.0011892 | up |
| ENSG00000139734 | DIAPH3 | 0.46 | 1.38 | 0.0018708 | up |
| ENSG00000196924 | FLNA | 0.46 | 1.38 | 2.479E-07 | up |
| ENSG00000127920 | GNG11 | 0.46 | 1.37 | 0.0019591 | up |
| ENSG00000180900 | SCRIB | 0.46 | 1.37 | 0.0005173 | up |
| ENSG00000107263 | RAPGEF1 | 0.46 | 1.37 | 0.0004503 | up |
| ENSG00000140859 | KIFC3 | 0.45 | 1.37 | 0.0002446 | up |
| ENSG00000184009 | ACTG1 | 0.45 | 1.37 | 2.07E-07 | up |
| ENSG00000186063 | AIDA | 0.45 | 1.37 | 0.0013062 | up |
| ENSG00000112787 | FBRSL1 | 0.45 | 1.37 | 0.00292 | up |
| ENSG00000173456 | RNF26 | 0.45 | 1.37 | 0.0021041 | up |
| ENSG00000112759 | SLC29A1 | 0.45 | 1.37 | 0.0028382 | up |
| ENSG00000187957 | DNER | 0.45 | 1.37 | 0.0016393 | up |
| ENSG00000149564 | ESAM | 0.45 | 1.36 | 0.0011363 | up |
| ENSG00000110713 | NUP98 | 0.45 | 1.36 | 4.984E-05 | up |
| ENSG00000186432 | KPNA4 | 0.45 | 1.36 | 0.0007781 | up |
| ENSG00000124942 | AHNAK | 0.45 | 1.36 | 2.559E-07 | up |
| ENSG00000068489 | PRR11 | 0.44 | 1.36 | 0.0017853 | up |
| ENSG00000013588 | GPRC5A | 0.44 | 1.36 | 2.437E-05 | up |
| ENSG00000145860 | RNF145 | 0.44 | 1.36 | 0.001036 | up |
| ENSG00000107404 | DVL1 | 0.44 | 1.36 | 0.000276 | up |
| ENSG00000116199 | FAM20B | 0.44 | 1.35 | 0.0019772 | up |
| ENSG00000167693 | NXN | 0.43 | 1.35 | 0.0019923 | up |
| ENSG00000071127 | WDR1 | 0.43 | 1.35 | 0.0002035 | up |
| ENSG00000127603 | MACF1 | 0.43 | 1.35 | 0.0001902 | up |
| ENSG00000082781 | ITGB5 | 0.43 | 1.35 | 0.0003552 | up |
| ENSG00000159176 | CSRP1 | 0.43 | 1.35 | 1.267E-05 | up |
| ENSG00000176788 | BASP1 | 0.43 | 1.35 | 6.203E-07 | up |
| ENSG00000130559 | CAMSAP1 | 0.43 | 1.35 | 0.0020984 | up |
| ENSG00000206527 | PTPLB | 0.43 | 1.34 | 0.0004251 | up |
| ENSG00000024422 | EHD2 | 0.42 | 1.34 | 0.0019063 | up |
| ENSG00000130402 | ACTN4 | 0.42 | 1.34 | 2.795E-06 | up |
| ENSG00000198826 | ARHGAP11A | 0.42 | 1.34 | 0.0011961 | up |
| ENSG00000004897 | CDC27 | 0.42 | 1.34 | 0.0022454 | up |
| ENSG00000092820 | EZR | 0.42 | 1.34 | 4.212E-05 | up |
| ENSG00000071564 | TCF3 | 0.42 | 1.34 | 0.0011129 | up |
| ENSG00000135387 | CAPRIN1 | 0.42 | 1.34 | 2.848E-05 | up |
| ENSG00000111331 | OAS3 | 0.42 | 1.34 | 0.0005359 | up |
| ENSG00000160007 | ARHGAP35 | 0.42 | 1.33 | 0.0012055 | up |
| ENSG00000103769 | RAB11A | 0.42 | 1.33 | 0.0030749 | up |
| ENSG00000120694 | HSPH1 | 0.41 | 1.33 | 0.0008147 | up |
| ENSG00000198901 | PRC1 | 0.41 | 1.33 | 0.0003233 | up |
| ENSG00000204389 | HSPA1A | 0.41 | 1.33 | 0.002818 | up |
| ENSG00000198363 | ASPH | 0.41 | 1.33 | 5.47E-05 | up |
| ENSG00000007202 | KIAA0100 | 0.41 | 1.33 | 0.0003568 | up |
| ENSG00000167972 | ABCA3 | 0.41 | 1.32 | 0.0018415 | up |
| ENSG00000038382 | TRIO | 0.41 | 1.32 | 0.0001156 | up |
| ENSG00000149639 | SOGA1 | 0.40 | 1.32 | 0.0010969 | up |
| ENSG00000110090 | CPT1A | 0.40 | 1.32 | 0.0002181 | up |
| ENSG00000112984 | KIF20A | 0.40 | 1.32 | 0.0023564 | up |
| ENSG00000106244 | PDAP1 | 0.40 | 1.32 | 0.0001207 | up |
| ENSG00000120742 | SERP1 | 0.40 | 1.31 | 0.0017849 | up |
| ENSG00000247556 | OIP5-AS1 | 0.39 | 1.31 | 0.0015906 | up |
| ENSG00000169679 | BUB1 | 0.39 | 1.31 | 0.0018526 | up |
| ENSG00000175216 | CKAP5 | 0.39 | 1.31 | 0.0002258 | up |
| ENSG00000162909 | CAPN2 | 0.39 | 1.31 | 9.678E-05 | up |
| ENSG00000115484 | CCT4 | 0.38 | 1.30 | 0.0004206 | up |
| ENSG00000105993 | DNAJB6 | 0.38 | 1.30 | 0.0020734 | up |
| ENSG00000164828 | SUN1 | 0.38 | 1.30 | 0.0008742 | up |
| ENSG00000173473 | SMARCC1 | 0.38 | 1.30 | 0.0003827 | up |
| ENSG00000102172 | SMS | 0.38 | 1.30 | 0.0003234 | up |
| ENSG00000132142 | ACACA | 0.38 | 1.30 | 0.0003798 | up |
| ENSG00000160208 | RRP1B | 0.37 | 1.29 | 0.0031089 | up |
| ENSG00000162402 | USP24 | 0.37 | 1.29 | 0.0029743 | up |
| ENSG00000188976 | NOC2L | 0.37 | 1.29 | 0.0009788 | up |
| ENSG00000140575 | IQGAP1 | 0.37 | 1.29 | 4.868E-05 | up |
| ENSG00000009335 | UBE3C | 0.37 | 1.29 | 0.0011747 | up |
| ENSG00000125970 | RALY | 0.37 | 1.29 | 0.0011375 | up |
| ENSG00000133030 | MPRIP | 0.36 | 1.29 | 0.0005154 | up |
| ENSG00000175115 | PACS1 | 0.36 | 1.29 | 0.0032997 | up |
| ENSG00000099901 | RANBP1 | 0.36 | 1.29 | 0.0011999 | up |
| ENSG00000128567 | PODXL | 0.36 | 1.28 | 0.0001556 | up |
| ENSG00000168439 | STIP1 | 0.36 | 1.28 | 0.000286 | up |
| ENSG00000109685 | WHSC1 | 0.35 | 1.28 | 0.0017462 | up |
| ENSG00000101444 | AHCY | 0.34 | 1.27 | 0.0029678 | up |
| ENSG00000047410 | TPR | 0.34 | 1.27 | 0.0029202 | up |
| ENSG00000197081 | IGF2R | 0.34 | 1.27 | 0.0017063 | up |
| ENSG00000129250 | KIF1C | 0.34 | 1.27 | 0.0010791 | up |
| ENSG00000142949 | PTPRF | 0.34 | 1.26 | 0.001142 | up |
| ENSG00000090621 | PABPC4 | 0.33 | 1.26 | 0.0031936 | up |
| ENSG00000115306 | SPTBN1 | 0.33 | 1.26 | 0.000484 | up |
| ENSG00000117724 | CENPF | 0.33 | 1.25 | 0.0013391 | up |
| ENSG00000117335 | CD46 | 0.32 | 1.25 | 0.0009723 | up |
| ENSG00000170606 | HSPA4 | 0.32 | 1.25 | 0.0025858 | up |
| ENSG00000064042 | LIMCH1 | 0.32 | 1.25 | 0.0029796 | up |
| ENSG00000135624 | CCT7 | 0.31 | 1.24 | 0.0010692 | up |
| ENSG00000127481 | UBR4 | 0.30 | 1.24 | 0.0032109 | up |
| ENSG00000088247 | KHSRP | 0.30 | 1.23 | 0.0027459 | up |
| ENSG00000044574 | HSPA5 | 0.30 | 1.23 | 0.0005441 | up |
| ENSG00000164924 | YWHAZ | 0.29 | 1.22 | 0.0016654 | up |
| ENSG00000198886 | MT-ND4 | 0.28 | 1.21 | 0.0005874 | up |
| ENSG00000158710 | TAGLN2 | 0.27 | 1.20 | 0.0023448 | up |
| ENSG00000182718 | ANXA2 | 0.27 | 1.20 | 0.002274 | up |

**Table S4. Identification of SKA3 binding proteins in LSCC cells by CoIP and mass spectrometry.**

| **No.** | **Protein accession and name** |
| --- | --- |
| 1 | sp\|Q96BD8\|SKA1_HUMAN |
| 2 | sp\|Q8WVK7\|SKA2_HUMAN |
| 3 | sp\|Q71U36\|TBA1A_HUMAN |
| 4 | sp\|Q9UKK9\|NUDT5_HUMAN |
| 5 | sp\|Q9BVJ6\|UT14A_HUMAN |
| 6 | sp\|P30876\|RPB2_HUMAN |
| 7 | sp\|Q8WUM0\|NU133_HUMAN |
| 8 | sp\|O00425\|IF2B3_HUMAN |
| 9 | sp\|Q9NY93\|DDX56_HUMAN |
| 10 | sp\|Q9NZI7\|UBIP1_HUMAN |
| 11 | sp\|Q9NW13\|RBM28_HUMAN |
| 12 | sp\|Q9Y2W1\|TR150_HUMAN |
| 13 | sp\|Q1KMD3\|HNRL2_HUMAN |
| 14 | sp\|P55735\|SEC13_HUMAN |
| 15 | sp\|A6NKT7\|RGPD3_HUMAN |
| 16 | sp\|Q96KR1\|ZFR_HUMAN |
| 17 | sp\|Q12874\|SF3A3_HUMAN |
| 18 | sp\|P42285\|SK2L2_HUMAN |
| 19 | sp\|Q9BSC4\|NOL10_HUMAN |
| 20 | sp\|O75400\|PR40A_HUMAN |
| 21 | sp\|Q14157\|UBP2L_HUMAN |
| 22 | sp\|Q8N3U4\|STAG2_HUMAN |
| 23 | sp\|O95232\|LC7L3_HUMAN |
| 24 | sp\|P52294\|IMA5_HUMAN |
| 25 | sp\|Q9P2J5\|SYLC_HUMAN |
| 26 | sp\|Q9UIG0\|BAZ1B_HUMAN |
| 27 | sp\|Q9NRG9\|AAAS_HUMAN |
| 28 | sp\|O00139\|KIF2A_HUMAN |
| 29 | sp\|P53350\|PLK1_HUMAN |
| 30 | sp\|O15042\|SR140_HUMAN |
| 31 | sp\|Q8NC56\|LEMD2_HUMAN |
| 32 | sp\|Q96SB4\|SRPK1_HUMAN |
| 33 | sp\|Q9BTE3\|MCMBP_HUMAN |
| 34 | sp\|Q9H6F5\|CCD86_HUMAN |
| 35 | sp\|Q9HAV0\|GBB4_HUMAN |
| 36 | sp\|Q9UG63\|ABCF2_HUMAN |
| 37 | sp\|Q9UNE7\|CHIP_HUMAN |
| 38 | sp\|Q13347\|EIF3I_HUMAN |
| 39 | sp\|Q15208\|STK38_HUMAN |
| 40 | sp\|P26196\|DDX6_HUMAN |
| 41 | sp\|P30049\|ATPD_HUMAN |
| 42 | sp\|P35251\|RFC1_HUMAN |
| 43 | sp\|P48729\|KC1A_HUMAN |
| 44 | sp\|P49959\|MRE11_HUMAN |
| 45 | sp\|P61158\|ARP3_HUMAN |
| 46 | sp\|P82930\|RT34_HUMAN |
| 47 | sp\|Q9H583\|HEAT1_HUMAN |
| 48 | sp\|Q8N684\|CPSF7_HUMAN |
| 49 | sp\|O60762\|DPM1_HUMAN |
| 50 | sp\|P08621\|RU17_HUMAN |
| 51 | sp\|P49756\|RBM25_HUMAN |
| 52 | sp\|Q9BQ75\|CMS1_HUMAN |
| 53 | sp\|Q9NWH9\|SLTM_HUMAN |
| 54 | sp\|Q8WYA6\|CTBL1_HUMAN |
| 55 | sp\|Q9NRH3\|TBG2_HUMAN |
| 56 | sp\|P61218\|RPAB2_HUMAN |
| 57 | sp\|Q14978\|NOLC1_HUMAN |
| 58 | sp\|Q7Z6I8\|CE024_HUMAN |
| 59 | sp\|Q9NSD9\|SYFB_HUMAN |
| 60 | sp\|Q66PJ3\|AR6P4_HUMAN |
| 61 | sp\|Q969Q0\|RL36L_HUMAN |
| 62 | sp\|Q12888\|TP53B_HUMAN |
| 63 | sp\|O43447\|PPIH_HUMAN |
| 64 | sp\|Q9GZR7\|DDX24_HUMAN |
| 65 | sp\|Q9H0U3\|MAGT1_HUMAN |
| 66 | sp\|Q9NZB2\|F120A_HUMAN |
| 67 | sp\|Q9Y2U8\|MAN1_HUMAN |
| 68 | sp\|Q9Y305\|ACOT9_HUMAN |

**Table S5. Clinical features of 53 LSCC samples for transcriptome sequencing.**

| **Parameters** | **Number of Cases（%）** |
| --- | --- |
| Age |  |
| <60 | 26（49.1） |
| ≥60 | 27（50.9） |
| Sex |  |
| Female | 3（5.7） |
| Male | 50（94.3） |
| Primary Site |  |
| Glottic | 27（50.9） |
| Supraglottic | 21（39.6） |
| Subglottic | 2（3.8） |
| Transglottic | 3 (5.7) |
| Differentiation |  |
| High | 7（13.2） |
| Medium | 36（67.9） |
| Low | 10（18.9） |
| T Staging**^1^** |  |
| T1 | 19（35.9） |
| T2 | 13（24.5） |
| T3 | 16（30.2） |
| T4 | 5（9.4） |
| Cervical lymph node metastasis |  |
| N0 | 39（73.6） |
| N+ | 14（26.4） |
| Distant metastasis |  |
| M0 | 53（100.0） |
| M1 | 0（0.0） |
| Clinical stage |  |
| I | 18（33.9） |
| II | 10（18.9） |
| III | 14（26.4） |
| IV | 11（20.8） |

^1^TNM Staging is referring to the 8th UICC/AJCC TNM Staging Criteria

**Table S6**. **siRNA sequences for knockdown experiments.**

| **Target gene name** | **Sense sequence** | **Antisense sequence** |
| --- | --- | --- |
| SKA3 | CCACCUACCAAACAAUCACUA | UAGUGAUUGUUUGGUAGGUGG |
| MYC | GGAAACGACGAGAACAGUU | AACUGUUCUCGUCGUUUCC |
| YY1 | CCUCCUGAUUAUUCAGAAU | AUUCUGAAUAAUCAGGAGG |
| ETS1 | CUGGAAUUACUCACUGAUA | UAUCAGUGAGUAAUUCCAG |
| SP1 | CCCAAGUUUAUUUCUCUCU | AGAGAGAAAUAAACUUGGG |
| PLK1 | CCAACCAUUAACGAGCUGCUU | AAGCAGCUCGUUAAUGGUUGG |
| PTEN | CCACAGCUAGAACUUAUCAAA | UUUGAUAAGUUCUAGCUGUGG |
| HK2 | GUGGACAGGAUACGAGAAA | UUUCUCGUAUCCUGUCCAC |
| PFKFB3 | AGCUGCCUGGACAAAACAUG | CAUGUUUUGUCCAGGCAGCU |
| PDK1 | GCCUGCAAGAUGAUCUUUA | UAAAGAUCAUCUUGCAGGC |
| Negative control (NC) | UUCUCCGAACGUGUCACGU | ACGUGACACGUUCGGAGAA |

**Table S7. Sequences for shRNA plasmid construction for HCS.**

| **Gene symbol** | **Sense (5’- 3’)** | **Antisense (5’- 3’)** |
| --- | --- | --- |
| NC | CACCGATAGTAGGTGCCGGAATGTGGCTTCCTGTCAGACCACATTCCGGCACCTACTA | AAAATAGTAGGTGCCGGAATGTGGTCTGACAGGAAGCCACATTCCGGCACCTACTATC |
| KRT17 | CACCGGTGCGTACCATTGTGGAAGACTTCCTGTCAGATCTTCCACAATGGTACGCACC | AAAAGGTGCGTACCATTGTGGAAGATCTGACAGGAAGTCTTCCACAATGGTACGCACC |
| LAMC2 | CACCGCACCTGTATTCCTTGTAACTCTTCCTGTCAGAAGTTACAAGGAATACAGGTGC | AAAAGCACCTGTATTCCTTGTAACTTCTGACAGGAAGAGTTACAAGGAATACAGGTGC |
| COL1A1 | CACCGGGCAAGACAGTGATTGAATACTTCCTGTCAGATATTCAATCACTGTCTTGCCC | AAAAGGGCAAGACAGTGATTGAATATCTGACAGGAAGTATTCAATCACTGTCTTGCCC |
| POSTN | CACCGCAAACAGCTCAGAGTCTTCGCTTCCTGTCAGACGAAGACTCTGAGCTGTTTGC | AAAAGCAAACAGCTCAGAGTCTTCGTCTGACAGGAAGCGAAGACTCTGAGCTGTTTGC |
| GJA1 | CACCGGGCGTTAAGGATCGGGTTAACTTCCTGTCAGATTAACCCGATCCTTAACGCCC | AAAAGGGCGTTAAGGATCGGGTTAATCTGACAGGAAGTTAACCCGATCCTTAACGCCC |
| PLAU | CACCGCTGATATTCCATGAATGTATCAGGACTTCCTGTCAGATCCTGATACATTCATGGAATATCAG | AAAACTGATATTCCATGAATGTATCAGGATCTGACAGGAAGTCCTGATACATTCATGGAATATCAGC |
| SLC16A1 | CACCAGTTAAACTGTGAAGCTATTGGAATCTTCCTGTCAGAATTCCAATAGCTTCACAGTTTAAC | AAAAGTTAAACTGTGAAGCTATTGGAATTCTGACAGGAAGATTCCAATAGCTTCACAGTTTAACT |
| COL5A2 | CACCGGGTTCATGCTACCCTGAAGTCTTCCTGTCAGAACTTCAGGGTAGCATGAACCC | AAAAGGGTTCATGCTACCCTGAAGTTCTGACAGGAAGACTTCAGGGTAGCATGAACCC |
| COL4A1 | CACCGCCAAGGGCGACAGAGGTTTGCTTCCTGTCAGACAAACCTCTGTCGCCCTTGGC | AAAAGCCAAGGGCGACAGAGGTTTGTCTGACAGGAAGCAAACCTCTGTCGCCCTTGGC |
| COL12A1 | CACCGGAGAACACTGGAGAACTTGACTTCCTGTCAGATCAAGTTCTCCAGTGTTCTCC | AAAAGGAGAACACTGGAGAACTTGATCTGACAGGAAGTCAAGTTCTCCAGTGTTCTCC |
| FSCN1 | CACCGCAAGTTTGTGACCTCCAAGACTTCCTGTCAGATCTTGGAGGTCACAAACTTGC | AAAAGCAAGTTTGTGACCTCCAAGATCTGACAGGAAGTCTTGGAGGTCACAAACTTGC |
| IFI27 | CACCGCGGCTGTCATTGCGAGGTTCCTTCCTGTCAGAGAACCTCGCAATGACAGCCGC | AAAAGCGGCTGTCATTGCGAGGTTCTCTGACAGGAAGGAACCTCGCAATGACAGCCGC |
| LUM | CACCGCTAACGAAGTCACTCTTAATCTTCCTGTCAGAATTAAGAGTGACTTCGTTAGC | AAAAGCTAACGAAGTCACTCTTAATTCTGACAGGAAGATTAAGAGTGACTTCGTTAGC |
| COL3A1 | CACCGCCCTACTGGTCCTCAGAACTCTTCCTGTCAGAAGTTCTGAGGACCAGTAGGGC | AAAAGCCCTACTGGTCCTCAGAACTTCTGACAGGAAGAGTTCTGAGGACCAGTAGGGC |
| FN1 | CACCGCTACCATCAGCGGCCTTAAACTTCCTGTCAGATTTAAGGCCGCTGATGGTAGC | AAAAGCTACCATCAGCGGCCTTAAATCTGACAGGAAGTTTAAGGCCGCTGATGGTAGC |
| CDH3 | CACCGGAATCCATTGAAGATCTTCCCTTCCTGTCAGAGGAAGATCTTCAATGGATTCC | AAAAGGAATCCATTGAAGATCTTCCTCTGACAGGAAGGGAAGATCTTCAATGGATTCC |
| LAMA3 | CACCGCTAACTTTGGATCAGTTAATCTTCCTGTCAGAATTAACTGATCCAAAGTTAGC | AAAAGCTAACTTTGGATCAGTTAATTCTGACAGGAAGATTAACTGATCCAAAGTTAGC |
| CENPF | CACCGCTACAAGGTCTGGACTTAAGCTTCCTGTCAGACTTAAGTCCAGACCTTGTAGC | AAAAGCTACAAGGTCTGGACTTAAGTCTGACAGGAAGCTTAAGTCCAGACCTTGTAGC |
| TOP2A | CACCGCACATCAAAGGAAGCTAAAGCTTCCTGTCAGACTTTAGCTTCCTTTGATGTGC | AAAAGCACATCAAAGGAAGCTAAAGTCTGACAGGAAGCTTTAGCTTCCTTTGATGTGC |
| COL4A2 | CACCGCAACAGAGGACTTGGTTTCTCTTCCTGTCAGAAGAAACCAAGTCCTCTGTTGC | AAAAGCAACAGAGGACTTGGTTTCTTCTGACAGGAAGAGAAACCAAGTCCTCTGTTGC |
| ECT2 | CACCGGAAGTCCCTGTTATAAAGATCTTCCTGTCAGAATCTTTATAACAGGGACTTCC | AAAAGGAAGTCCCTGTTATAAAGATTCTGACAGGAAGATCTTTATAACAGGGACTTCC |
| COL5A1 | CACCGCGAGGGTGAGACCTATTACTCTTCCTGTCAGAAGTAATAGGTCTCACCCTCGC | AAAAGCGAGGGTGAGACCTATTACTTCTGACAGGAAGAGTAATAGGTCTCACCCTCGC |
| ODC1 | CACCGCTTTCACGCTTGCAGTTAATCTTCCTGTCAGAATTAACTGCAAGCGTGAAAGC | AAAAGCTTTCACGCTTGCAGTTAATTCTGACAGGAAGATTAACTGCAAGCGTGAAAGC |
| SLC2A1 | CACCGCATCAACGCTGTCTTCTATTCTTCCTGTCAGAAATAGAAGACAGCGTTGATGC | AAAAGCATCAACGCTGTCTTCTATTTCTGACAGGAAGAATAGAAGACAGCGTTGATGC |
| COL6A3 | CACCGCTGCAGACCATCACCAATGACTTCCTGTCAGATCATTGGTGATGGTCTGCAGC | AAAAGCTGCAGACCATCACCAATGATCTGACAGGAAGTCATTGGTGATGGTCTGCAGC |
| SLC7A5 | CACCGGGAACATTGTGCTGGCATTACTTCCTGTCAGATAATGCCAGCACAATGTTCCC | AAAAGGGAACATTGTGCTGGCATTATCTGACAGGAAGTAATGCCAGCACAATGTTCCC |
| SEPINH1 | CACCG^*^CTATCAATCCAAGAACTTATTTGTACTTCCTGTCAGATACAAATAAGTTCTTGGATTGATAG | AAAACTATCAATCCAAGAACTTATTTGTATCTGACAGGAAGTACAAATAAGTTCTTGGATTGATAGC |
| GPNMB | CACCGGGATAATACTGGCCTGTTTGCTTCCTGTCAGACAAACAGGCCAGTATTATCCC | AAAAGGGATAATACTGGCCTGTTTGTCTGACAGGAAGCAAACAGGCCAGTATTATCCC |
| COL7A1 | CACCGCATCCAGCTACATCCTATCCCTTCCTGTCAGAGGATAGGATGTAGCTGGATGC | AAAAGCATCCAGCTACATCCTATCCTCTGACAGGAAGGGATAGGATGTAGCTGGATGC |
| MYO1B | CACCGATATTGAATTTGACTTTAAAGGCGCTTCCTGTCAGACGCCTTTAAAGTCAAATTCAATATC | AAAAGATATTGAATTTGACTTTAAAGGCGTCTGACAGGAAGCGCCTTTAAAGTCAAATTCAATATC |
| MCM2 | CACCGCACAAGGTACGTGGTGATATCAACCTTCCTGTCAGAGTTGATATCACCACGTACCTTGTGC | AAAAGCACAAGGTACGTGGTGATATCAACTCTGACAGGAAGGTTGATATCACCACGTACCTTGTGC |
| FOXM1 | CACCGCCAACCGCTACTTGACATTGCTTCCTGTCAGACAATGTCAAGTAGCGGTTGGC | AAAAGCCAACCGCTACTTGACATTGTCTGACAGGAAGCAATGTCAAGTAGCGGTTGGC |
| COL17A1 | CACCGCCAGGAGATTCAGCAGTACACTTCCTGTCAGATGTACTGCTGAATCTCCTGGC | AAAAGCCAGGAGATTCAGCAGTACATCTGACAGGAAGTGTACTGCTGAATCTCCTGGC |
| KPNA2 | CACCGGACATCAGAACAAACCAAGGCTTCCTGTCAGACCTTGGTTTGTTCTGATGTCC | AAAAGGACATCAGAACAAACCAAGGTCTGACAGGAAGCCTTGGTTTGTTCTGATGTCC |
| MKI67 | CACCGCACAAAGCAATGGCCTAAGACTTCCTGTCAGATCTTAGGCCATTGCTTTGTGC | AAAAGCACAAAGCAATGGCCTAAGATCTGACAGGAAGTCTTAGGCCATTGCTTTGTGC |
| PC1 | CACCGCTCCACGATGCTGAGATTGTCTTCCTGTCAGAACAATCTCAGCATCGTGGAGC | AAAAGCTCCACGATGCTGAGATTGTTCTGACAGGAAGACAATCTCAGCATCGTGGAGC |
| COL1A2 | CACCGCTGGCAGCCAGTTTGAATATCTTCCTGTCAGAATATTCAAACTGGCTGCCAGC | AAAAGCTGGCAGCCAGTTTGAATATTCTGACAGGAAGATATTCAAACTGGCTGCCAGC |
| TGFBI | CACCGGGACATGCTCACTATCAACGCTTCCTGTCAGACGTTGATAGTGAGCATGTCCC | AAAAGGGACATGCTCACTATCAACGTCTGACAGGAAGCGTTGATAGTGAGCATGTCCC |
| SPAG5 | CACCGGACCTGGCTATGAAGGATGACTTCCTGTCAGATCATCCTTCATAGCCAGGTCC | AAAAGGACCTGGCTATGAAGGATGATCTGACAGGAAGTCATCCTTCATAGCCAGGTCC |
| PHLDB2 | CACCGGCATTGACATTATTGGTAATCAACCTTCCTGTCAGAGTTGATTACCAATAATGTCAATGCC | AAAAGGCATTGACATTATTGGTAATCAACTCTGACAGGAAGGTTGATTACCAATAATGTCAATGCC |
| TP63 | CACCGGAACTCATGCAGTACCTTCCCTTCCTGTCAGAGGAAGGTACTGCATGAGTTCC | AAAAGGAACTCATGCAGTACCTTCCTCTGACAGGAAGGGAAGGTACTGCATGAGTTCC |
| TNC | CACCGGAAGACACCGTTGGCCAAATCTTCCTGTCAGAATTTGGCCAACGGTGTCTTCC | AAAAGGAAGACACCGTTGGCCAAATTCTGACAGGAAGATTTGGCCAACGGTGTCTTCC |
| AHGAP11A | CACCGGAAGTAATCTTCACGCATTGCTTCCTGTCAGACAATGCGTGAAGATTACTTCC | AAAAGGAAGTAATCTTCACGCATTGTCTGACAGGAAGCAATGCGTGAAGATTACTTCC |
| SULF1 | CACCGGCATGACTAAGAAGCTTAAATGTTCTTCCTGTCAGAAACATTTAAGCTTCTTAGTCATGCC | AAAAGGCATGACTAAGAAGCTTAAATGTTTCTGACAGGAAGAACATTTAAGCTTCTTAGTCATGCC |
| FANCI | CACCGGCTAATTCAGTCATGCAAACTCTGCTTCCTGTCAGACAGAGTTTGCATGACTGAATTAGCC | AAAAGGCTAATTCAGTCATGCAAACTCTGTCTGACAGGAAGCAGAGTTTGCATGACTGAATTAGCC |
| HIST1H2AL | CACCGCTCCGCAAGGGCAACTATGCCTTCCTGTCAGAGCATAGTTGCCCTTGCGGAGC | AAAAGCTCCGCAAGGGCAACTATGCTCTGACAGGAAGGCATAGTTGCCCTTGCGGAGC |
| SKA3 | CACCGCGGTACATCGTATCCCAAGTCTTCCTGTCAGAACTTGGGATACGATGTACCGC | AAAAGCGGTACATCGTATCCCAAGTTCTGACAGGAAGACTTGGGATACGATGTACCGC |
| ITGA6 | CACCGCACAGCAACCTTGAACATTCCTTCCTGTCAGAGAATGTTCAAGGTTGCTGTGC | AAAAGCACAGCAACCTTGAACATTCTCTGACAGGAAGGAATGTTCAAGGTTGCTGTGC |
| SPAC | CACCGGATGAGGACAACAACCTTCTCTTCCTGTCAGAAGAAGGTTGTTGTCCTCATCC | AAAAGGATGAGGACAACAACCTTCTTCTGACAGGAAGAGAAGGTTGTTGTCCTCATCC |
| CD276 | CACCGGGTGGTGCTGGGTGCGAATGCTTCCTGTCAGACATTCGCACCCAGCACCACCC | AAAAGGGTGGTGCTGGGTGCGAATGTCTGACAGGAAGCATTCGCACCCAGCACCACCC |

**Table S8**. **Primer sequences for qPCR experiments.**

| **Gene symbol** | **Forward primer** | **Reverse primer** |
| --- | --- | --- |
| SKA3 | GCTCAGCATGGACCCTATCC | TGGATAATCTTCAAAGTCGCTTTCC |
| MYC | GGAGGCTATTCTGCCCATTT | GAGTCGTAGTCGAGGTCATAGT |
| YY1 | ATTGACCTCTCAGATCCCAAAC | TTTCTCATGGCCGAGTTATCC |
| ETS1 | GACCGTGCTGACCTCAATAA | CATCTCCTGTCCAGCTGATAAA |
| SP1 | CCACTCCTTCAGCCCTTATTAC | GCCTGATCTCAGAAGCCATT |
| PLK1 | CAACACGCCTCATCCTCTAC | CAAGTGCTCGCTCATGTAATTG |
| HK2 | AGGTCCTGATGCGGTTGG | TCGCCTTTGTTCTCCTTGAT |
| PFKFB3 | CGAGAACGAGCACAACCT | TCAGAGCACTGGCAAACT |
| PDK1 | AATCACCAGGACAGCCAATA | CTCGGTCACTCATCTTCACA |
| 18S rRNA | CCTGGATACCGCAGCTAGGA | GCGGCGCAATACGAATGCCCC |
| MYC-ChIP-1 | GTGCTTGTCAGTAAAATACCA | TACTGCAACTGTGGACCACGT |

**Table S9. Clinical features of 165 LSCC samples for IHC staining.**

| **Parameters** | **Number of Cases（n, %）** |
| --- | --- |
| **Age** |  |
| <60 | 80（48.5） |
| ≥60 | 85（51.5） |
| **Sex** |  |
| Female | 7（4.2） |
| Male | 158（95.8） |
| **Primary Site** |  |
| Glottic | 72（43.6） |
| Supraglottic | 87（52.7） |
| Subglottic | 6（3.7） |
| **Differentiation** |  |
| Well | 44（26.7） |
| Moderately | 88（53.3） |
| Poorly | 33（20.0） |
| **T Staging^1^** |  |
| T1 | 37（22.4） |
| T2 | 37（22.4） |
| T3 | 43（26.1） |
| T4 | 48（29.1） |
| **Cervical lymph node metastasis** |  |
| N0 | 119（72.1） |
| N+ | 46（27.9） |
| **Distant metastasis** |  |
| M0 | 152（92.1） |
| M1 | 13（7.9） |
| **Clinical stage** |  |
| I | 33（20.0） |
| II | 31（18.8） |
| III | 35（21.2） |
| IV | 66（40.0） |

^1^TNM Staging is referring to the 8th UICC/AJCC TNM Staging Criteria
